# Supplementary material for: ‘Our project, your problem?’ A case study of the WHO’s mRNA technology transfer programme in South Africa
Source: PLOS Glob Public Health. 2024 Sep 23;4(9):e0003173. doi: 10.1371/journal.pgph.0003173 (PMC11419367; doi:10.1371/journal.pgph.0003173)
Supplement: S1 Letter — (PDF) [file pgph.0003173.s001.pdf]

Global Affairs Canada
Affaires mondiales Canada

125 Sussex Drive
Ottawa ON K1A 0G2

Our File
A-2023-00188 / ES2

Matthew Herder
6061 University Avenue
Halifax, Nova Scotia
B3H 4R2

Matthew.Herder@dal.ca

Matthew Herder,

This is in reply to your request under the *Access to Information Act* which was received on April 25, 2023, for the following:

> **"All records pertaining to the Government of Canada's financial support for the World Health Organization's mRNA technology transfer hub that is based in South Africa, between the dates of March 16, 2023 and April 24, 2023. Specifically, I request all agreements, contracts, correspondence, emails, reports, meeting minutes, briefing notes, and presentations about: 1) the amount of funding to be provided by the Government of Canada; 2) how the funding would be transferred to the hub; and, 3) what, if any, conditions were placed upon how the funding that was made available could be used by actors involved in the mRNA hub. For greater clarity, I am seeking records that specify whether the funding provided could be used for some purposes (e.g. supporting the involvement of certain companies in the hub) but not others (e.g., specific types of research related to mRNA vaccines). Also, these records should include all correspondence, agreements, etc. between the Government of Canada, the World Health Organization, the not-for-profit foundation the Medicines Patent Pool, and any other actors involved in the hub."**

Enclosed are the documents that respond to your request. Please note that some of the information contained in the documents has been exempted pursuant to subsection 19(1) and paragraph 20(1)(b) of the Act. You may find details of the exemptions invoked at the following website: http://laws-lois.justice.gc.ca/eng/acts/A-1/index.html

You are entitled to complain to the Information Commissioner concerning the processing of your request within 60 days of the receipt of this notice. In the event you decide to avail yourself of this right, your notice of complaint should be addressed to the Office of the Information Commissioner of Canada, 30 Victoria Street, Gatineau, QC, K1A 1H3

Should you have any questions, please do not hesitate to contact Erin Scheel at 343-203-1362 or by email at erin.scheel@international.gc.ca.

Yours sincerely,

Alexandre Drago
Director
Access to Information and Privacy Protection Division

Enclosure: Pages 1- 7

Canada
