# Supplementary material for: ‘Our project, your problem?’ A case study of the WHO’s mRNA technology transfer programme in South Africa
Source: PLOS Glob Public Health. 2024 Sep 23;4(9):e0003173. doi: 10.1371/journal.pgph.0003173 (PMC11419367; doi:10.1371/journal.pgph.0003173)
Supplement: S1 Document — (PDF) [file pgph.0003173.s002.pdf]

GAC - Released under the Access to Informations Act/
AMC - Divulgué en vertu de la loi sur l'accès à l'information

PROJECT MEMORANDUM

Global Affairs Canada | Affaires mondiales Canada

UNCLASSIFIED

BPTS: 00573-2022

The Minister of International Development

CC: The Minister of Foreign Affairs
The Minister of International Trade, Export Promotion, Small Business and Economic Development

COVID-19 mRNA Technology Transfer and Manufacturing Hub

Project Number and Selection Mechanism: P011103, Department-initiated
Funding Recipient: Medicines Patent Pool
Country/Region: South Africa
Gender Equality Policy Marker: GE-01, Partially integrated initiative
Environmental Integration Coding: Level 0, Not integrated
Funding and Duration: $15,000,000, Project duration up to 24 months
Action Areas: Human dignity (100%)

☒ New project ☐ Changes to an existing project ☐ Initiate a call

Time Sensitive – ☒ YES ☐ NO

A timely response by February 28, 2022, is critical for the department to disburse this funding in 2021/22 and deliver on prime ministerial commitments.

Summary

Recommendation

The purpose of this memorandum is to seek your approval to provide up to $15 million to the Medicines Patent Pool (MPP), a COVAX Facility Manufacturing Taskforce partner, to support the establishment of a technology transfer hub for mRNA COVID-19 vaccines in South Africa and facilitate production by a South African-based manufacturing facility. It also seeks your approval to enter into a grant agreement with the MPP for project implementation over 2 fiscal years (2021/22 to 2022/23).

Key commitments

The proposed program responds directly to commitments made by the Prime Minister at the G20 Summit on October 30, 2021, to invest $15 million to COVAX Manufacturing Taskforce partners, in support of the establishment of the South Africa technology transfer hub. This announcement builds on Canada's ongoing commitment within the Access to COVID-19 Tools (ACT)-Accelerator, G7 and G20 platforms to address barriers to equitable access to vaccines by improving global capacity for manufacture. The program is grounded in the principles of the Feminist International Assistance Policy.

Rationale

The impact of the failure to contain the spread of COVID-19 has been devastating worldwide. Canada has provided global leadership to support the ACT-Accelerator, G7 and G20 efforts to increase access to vaccines and improve bottlenecks in global supply chains to end the current pandemic. However, the unequal distribution of vaccine manufacturing continues to perpetuate inequalities in access to vaccines, not only prolonging the current crisis, but hampering efforts to equitably prevent the next pandemic.

Expected results

The overall aim of this project is to increase regional manufacturing capacity in southern Africa through the establishment of a COVID-19 mRNA Technology Transfer and Manufacturing Hub. The initiative would work with COVAX Facility partners through the MPP to establish a technology transfer hub for mRNA COVID-19 vaccines in South Africa, facilitate technology transfer to a South African-based recipient manufacturing facility, and establish vaccine production by the recipient facility. The project would support increased regional availability of mRNA vaccines for COVID-19 and explore the broader application of mRNA technologies toward additional infectious diseases.

GAC - Released under the Access to Informations Act/
AMC - Divulgué en vertu de la loi sur l'accès à l'information

Rationale

COVID-19 related deaths are now over 5 million, costing the global economy at least US$22 trillion by 2025, with a disproportionate impact on vulnerable populations in developing countries. The acute phase of the COVID-19 pandemic persists globally, with insufficient vaccination coverage driving high levels of infection and allowing for continued emergence of variants, which threaten the gains achieved in response efforts to date (including domestically).

Global leaders have affirmed the need to increase regional vaccine production capacity, including for lower-middle income countries to develop and produce vaccines best suited to their contexts. This COVID-19 mRNA Technology Transfer and Manufacturing Hub (the Hub) is the premier multilateral effort to establish technology transfer and production on the African continent, with life-saving implications for increased vaccine production and use of mRNA technologies beyond COVID-19. Funding for this program is drawn from the Prime Minister's Strategic Priorities Fund and would contribute to Canada's global and ministerial mandate commitments to finish the fight against the pandemic and ensure access to COVID-19 health interventions for people around the world.

The unequal distribution of biomanufacturing continues to perpetuate inequalities in access to vaccines over the medium and long term. Manufacturing of vaccines is largely concentrated in high income and select emerging economies, while Africa imports 99% of its vaccines and Latin America imports 96% of medical products. Currently, approximately 55% of capacity is located in East Asia (largely India and China), 40% in Europe and North America and less than 5% in Africa and South America. It is widely recognized that, sustainably oriented, the transfer of medical technologies, know-how and production capacity would have far-reaching implications to speed the end of the acute phase of the pandemic, strengthen health systems, improve access to primary healthcare, and work toward achieving universal health coverage in developing countries contexts.

The Access to COVID-19 Tools-Accelerator's COVAX Manufacturing Taskforce, created in early 2021, is the premier multilateral effort focused on establishing new and expanded sustainable vaccine manufacturing capacity in low and lower-middle income countries. COVAX has a critical role in coordinating such efforts, ultimately empowering countries to enhance their capability to respond to COVID-19 and potential future pandemics.

The WHO Secretariat, via the Multilateral Technology Transfer Initiative for Biologicals, the Medicines Patent Pool (MPP), and a steering committee of global experts and local consortium partners have joined in partnership to establish the first-ever COVID-19 mRNA Technology Transfer and Manufacturing Hub and recipient facility network on the African continent. The full scope of the initiative over 5 years (total budget €92 million, or roughly Can$130 million) is to establish voluntary technology transfer to several recipient facilities in Africa and Latin America. This initial phase of the project, supported by Canada's investment of $15 million, would establish the technology transfer hub itself (Afrigen Biologics), transfer technology of a locally-developed mRNA vaccine to a first recipient facility (Biovac Institute), and explore application of mRNA technologies beyond COVID-19 for other infectious diseases such as malaria, HIV and tuberculosis in partnership with the South African Medical Research Council.

The MPP would oversee the coordination, negotiation and implementation of all agreements, licensing and intellectual property transfer. The MPP has established a proven approach in negotiating agreements with patent holders, including, for example, for HIV antivirals, hepatitis C direct-acting antivirals, a tuberculosis treatment and, most recently, 2 COVID-19 antiviral drugs and a COVID-19 diagnostic technology. The resulting agreements have amply demonstrated that voluntary approaches to increasing access to health products are effective. Coordination of technology transfer, regulatory strengthening and technical assistance would also draw from the expertise of WHO's Headquarters science and access to medicines and health products divisions and WHO regional offices.

Gender Equality and the Empowerment of Women and Girls

The COVID-19 pandemic is disproportionally affecting poor and marginalized communities due to increased transmission in areas of dense population with weak sanitation and limited mitigation capacity. Women and children—who already constitute the majority of the world's poorest and most disadvantaged citizens—are

GAC - Released under the Access to Informations Act/
AMC - Divulgué en vertu de la loi sur l'accès à l'information

feeling the secondary impacts of COVID-19 most acutely. Since the outbreak of the virus, a number of gendered impacts have emerged, including increased incidences of gender-based violence and additional care burdens. Given that 70% of frontline healthcare workers are women, and women and girls dominate the social and service sectors globally, the risk of potential exposure to COVID-19 is heightened.

Notably, the COVID-19 pandemic has also exposed existing gaps in access to health services and health commodity supply chains that are inherently gendered and intersectional. These gaps extend into the healthcare sector, where women make up 70% of the global health and care workforce, but occupy just 25% of decision-making roles. In the biotech and manufacturing sector, preliminary research shows that underrepresentation of women increases as roles become more senior, from 45% representation at the organizational level, to 30% at the executive level and 16% at CEO level—numbers that are more stark with small and medium-sized operations. In South Africa, women make up less than 35% of the manufacturing workforce. Significant misperceptions continue to exist regarding women's abilities to execute manufacturing-related roles. Further, in South Africa, women have been more affected by unemployment as a result of the pandemic, with unemployment rates highest among black African women.

The Hub would contribute to an inclusive manufacturing sector by ensuring adequate gender responsiveness of selected hubs and spokes. Gender equality would be specifically considered as a criterion in the selection of new hubs and spokes. Recruitment and human resource approaches would result in women being empowered as key players in all operational areas, including the promotion of women in decision-making and leadership positions. As an example, the top 3 leadership positions at the first hub, Afrigen, are occupied by women. Additionally, the Hub would provide opportunities for women living in marginalized communities to participate in the manufacturing sector and compete in the marketplace, through proactively targeting those countries in the establishment of the hubs/spokes network which currently have lacking or limited (biologicals) manufacturing capacity.

Expected Results and Implementation

The ultimate aim of this project is the establishment of an mRNA technology transfer hub and recipient manufacturing facility network in South Africa that would facilitate the transfer of mRNA vaccine technology to locally develop and produce a COVID-19 mRNA vaccine and related mRNA technologies.

This project would, as a first objective, establish the technology transfer hub facility, which includes hub facilities prepared, vaccine technology received and/or developed, intellectual property agreements and regulatory processes reviewed, preclinical and clinical trials conducted, technology transfer program designed and biomanufacturing training center maintained. The second objective would result in recipient manufacturing facilities prepared, staff trained in mRNA technology, technology transferred from the Hub, phase 3 trials conducted and vaccines manufactured, with ultimate production at a first facility reaching 100 million doses per year for the southern African region.

Risk

☒ Project implementation risks

The impact of the global COVID-19 pandemic is unprecedented. The COVID-19 mRNA Technology Transfer and Manufacturing Hub is the first of its kind. It is designed to establish sustainable biomanufacturing capacity in the region and build human capital for regulations and biomanufacturing in surrounding lower-middle income countries. There are unknown variables that may affect the successful start-up of this technology transfer hub—including the trajectory of the pandemic and the emergence of new variants which could impact the efficacy of the mRNA vaccines the Hub would seek to reproduce. The department is mitigating this risk by working with experienced global health partners with a demonstrated ability in biomanufacturing.

☒ Funding risks

The total cost of the COVID-19 mRNA Technology Transfer and Manufacturing Hub is approximately €92 million over 5 years. At present, the MPP have raised approximately €52 million of the total needed budget. There is a risk that the full cost to operationalizing the Hub would not be met. To mitigate this risk, COVAX Manufacturing Task Force partners have developed a workplan centred around scalable objectives

GAC - Released under the Access to Informations Act/
AMC - Divulgué en vertu de la loi sur l'accès à l'information

that focus first on the development and transfer of mRNA technology, and should additional funding be secured, the development and establishment of a pipeline of other vaccine candidates (tuberculosis, HIV, etc.). The development of other vaccine candidates, while important to the Hub's overall sustainability, is not critical to Canada's objective of increasing COVID-19 vaccine equity in the region. Note that other donors have confirmed support for the Hub (Belgium, the European Union, Germany, Norway) though amounts have not been communicated publicly and additional agreements are imminently pending with additional donors.

Other Considerations

Canada's investment in the Hub at the G20 signaled early support alongside other confirmed and interested donors such as France, Belgium, the European Commission, Germany, the United Kingdom and the United States. The policy on cost-share has been waived for this project.

Communications

The $15-million commitment to COVAX Manufacturing Task Force partners was announced by the Prime Minister at the G20 Summit on October 30, 2021, and has been highlighted at every opportunity, including on the department's website, in media responses, and in a video that aired on Global Affairs Canada social media channels in December 2021. Should the allocation to the MPP be approved, this investment could be highlighted in remarks by you at high-level international events; for instance, at the (to be confirmed) hub consortium funder's launch (tentatively slated for the first quarter or early second quarter of this year).

Any content by partners about significant milestones and results could be amplified on Global Affairs Canada social media accounts and in other communications (for example, web pages), as appropriate. Canada's contribution would be publicly recognized by partners through their public communications and project activities, including events, website, social media and publications. A description of the project, key results and stories from the field (if available) would be posted on the department's website and amplified on mission social media channels, as appropriate.

Due Diligence

The department has assessed the past performance, technical, managerial, administrative and financial capacity of the fund recipients and is satisfied that they are competent and would be able to deliver the intended development results for their projects without impeding upon their capacity to deliver on existing commitments.

☑ Completed: No concern was raised

As part of the due diligence process, these projects have been assessed against contracting and financial management requirements, and gender equality and environmental sustainability policies. These projects meet departmental due diligence requirements.

These projects have been assessed against the requirements of subsection 4(1) of the *Official Development Assistance Accountability Act* to ensure that they contribute to poverty reduction, take into account the perspectives of the poor, and are consistent with international human rights standards. Equitable access to life-saving COVID-19 countermeasures, undertaken for, and in collaboration with low income countries and lower-middle income countries, would help reduce poverty and uphold international human rights. Because it complies with the criteria set out in the legislation, it shall be included in the annual report to Parliament as official development assistance.

GAC - Released under the Access to Informations Act/
AMC - Divulgué en vertu de la loi sur l'accès à l'information

Recommendations

It is recommended that you:

1) Approve up to $15 million to the MPP to support the establishment of a technology transfer hub for mRNA COVID-19 vaccines in South Africa and facilitate production by a South African-based manufacturing facility; and

2) Authorize the department to enter into a grant agreement with the MPP for project implementation.

for Christopher MacLennan
Deputy Minister of International Development

February 28, 2022
Date

☒ I AGREE with the recommendation.
☐ I DO NOT AGREE with the recommendation.

Harjit S. Sajjan
Minister of International Development

March 4, 2022
Date

GAC - Released under the Access to Informations Act/
AMC - Divulgué en vertu de la loi sur l'accès à l'information

Annex A

Project Funding and Notional Disbursement Schedule

(Figures in millions of Canadian dollars)

	2022/23	2023/24	2024/25	2025/26	Total
**Total project budget (A+B)**					**130.00**[1]
**Total Global Affairs Canada contribution (A)**	**15.00**	**0**	**0**	**0**	**15.00**
Monitoring and evaluation[2]	*TBD*	*TBD*	*TBD*	*TBD*	*TBD*
**Other contributions (B)**[3]					
France					
Belgium					
The European Union					
Germany					
Norway					
The African Union					

[1] €92 million is the project total. The exchange rate used is 1.62 Canadian dollars.
[2] The Medicines Patent Pool, in consultation with donors, would identify the specific monitoring and evaluation needs associated with this project.
[3] France has publically announced its investment in the Hub. Germany, Belgium, the European Union, Norway and the African Union are confirmed but not yet announced. Agreements are pending with additional donors.

GAC - Released under the Access to Informations Act/
AMC - Divulgué en vertu de la loi sur l'accès à l'information

Global Affairs Canada | Affaires mondiales Canada

UNCLASSIFIED

BPTS: 04071-2022

PROJECT MEMORANDUM

The Minister of International Development

CC: The Minister of Foreign Affairs
The Minister of International Trade, Export Promotion, Small Business and Economic Development

Canada's additional support to the Access to COVID-19 Tools (ACT)—Accelerator to advance COVID-19 vaccination

Project Number and Selection Mechanism: Multiple. Department initiated
Funding Recipient: Multiple
Country/Region: Multiple
Gender Equality Policy Marker: For individual projects ratings, see Annex B.
Environmental Integration Coding: For individual projects ratings, see Annex B.
Original Total Funding and Duration: $200,000,000. Project durations up to 18 months.
Revised Funding and Duration: $317,000,000. Project durations up to 24 months.
Action Areas: Human dignity

☐ New projects ☑ Changes to existing projects ☐ Initiate a call
Time Sensitive – ☑ YES ☐ NO
A timely response is critical for the department to disburse this funding in FY 2022-2023 and deliver on Prime Ministerial commitments.

Summary

Recommendation

The purpose of this omnibus memorandum is to seek approval to increase the value of 4 previously approved projects with ACT-Accelerator Partners (WHO, UNICEF, Pan-American Health Organization (PAHO) and Medicines Patent Pool) for a total value of $117,000,000. It also seeks approval to enter into 1 agreement (PAHO) and to amend 3 existing agreements (WHO, UNICEF, Medicines Patent Pool) for project implementation, as well as to extend project durations from 18 to 24 months in order to enable significant program scale-up and reinforce sustainability. As part of Canada's Global Initiative for Vaccine Equity (CanGIVE), the proposed programming would pursue a comprehensive approach to advancing COVID-19 vaccination coverage while also strengthening health systems and enhancing preparedness for future health crises in 12 countries.[1] Individual funding allocations and detailed project descriptions can be found in Annexes A and B.

Key Commitments

An omnibus memorandum signed on September 27, 2022, (BPTS 03030-2022) approved the allocation of $615,000,000 out of the total $732,000,000 for the ACT-Accelerator from Budget 2022, announced by the Prime Minister in May 2022. All 4 projects recommended for approval in this memorandum were approved in the original memorandum. A retention of $117,000,000 in funds was undertaken to ensure that Canada has reserved the ability to be responsive to the evolving COVID-19 epidemiology, country vaccination needs and emergent health priorities. In addition, this funding was retained to enable Canada to meet the Prime Ministerial commitment to donate the equivalent of 200 million doses of COVID-19 vaccines by the end of 2022. Given that projects outlined in this memorandum would advance vaccine delivery, demand and local production, the $117,000,000 in funding would be counted as dose equivalents, fulfilling Canada's dose donation commitment. Dose equivalent calculations utilise the OECD dose donation conversion rate of $6.72 USD per dose, in line with the agreed G7 methodology.

[1] Bangladesh, Colombia, Côte d'Ivoire, the Democratic Republic of Congo, Ghana, Haiti, Jamaica, Mozambique, Nigeria, Senegal, South Africa and Tanzania.

GAC - Released under the Access to Informations Act/
AMC - Divulgué en vertu de la loi sur l'accès à l'information

Rationale

Nearly 3 years into the COVID-19 pandemic, low vaccination rates and overstretched health systems in lower-middle income countries (LMICs) continue to present significant risks to achieving long-term control of the COVID-19 virus globally. Many high risk populations remain unprotected against COVID-19, even as countries begin to integrate COVID-19 vaccination within under-resourced routine immunization and health services. Based on the current pandemic landscape, the proposed project increases in this memorandum would build upon Canada's support to trusted ACT-Accelerator partners by supporting vaccine-related activities that seek to advance COVID-19 vaccination coverage while also strengthening health systems and enhancing preparedness for future health crises.

Expected Results

Globally, COVID-19 vaccines are in over-supply, and countries are currently requesting urgent assistance to bolster health system capacity to generate demand for and deliver COVID-19 vaccines and routine health services. The 4 CanGIVE projects recommended in this memorandum would contribute to increased equitable access to COVID-19 vaccines, improve deployment and delivery of essential health services and COVID-19 medical countermeasures, and strengthen health systems to increase resilience in the face of health crises.

The proposed allocations in this memorandum would contribute to the Departmental Results Framework outcome of improving physical, social and economic well-being for the poorest and most vulnerable, particularly for women and girls, in countries where Canada engages. Funding would mitigate the impacts of the pandemic on the world's poorest and most marginalized, particularly women and girls, and advance Canada's Feminist International Assistance Policy.

For specific activities, expected outcomes and implementation of the proposed amendments, please see Annex B.

Rationale

Many regions continue to experience resurgences in COVID-19 cases, resulting in exacerbated strain on health systems, increased hospitalizations and backsliding in routine immunization. New weekly cases of COVID-19 are again increasing in the Western Pacific (+18%), Southeast Asia (+15%) and Americas (+12%) regions while deaths have also increased in the Western Pacific by 14%.[2] The ongoing impacts of the pandemic have contributed to the largest sustained decline in childhood vaccinations in 30 years, resulting in 25 million children worldwide missing out on their diphtheria, tetanus and pertussis vaccines.[3]

There is a continued need for COVID-19 public health measures, particularly vaccination, as countries move towards recovery and resumption of regular health service provision. While nearly 13 billion COVID-19 vaccine doses have been administered globally thus far, many low-income countries (LICs) remain below 20% coverage, particularly in Africa and in countries experiencing humanitarian contexts. The biggest barrier to increased vaccination in LICs is no longer the availability of vaccines, but limited absorptive and delivery capacity, as well as low demand for COVID-19 vaccines due to low risk-perception and misinformation. Continued global action is crucial to mitigate the ongoing risks from COVID-19 and ensure that countries have access to, and can deliver, the vaccines needed to manage the virus sustainably. In this important phase approaching endemic COVID-19 response, efforts must also strengthen the equity and resilience of health systems so that countries can better respond to health crises while also maintaining routine immunization and essential public health care.

As part of Canada's Global Initiative for Vaccine Equity (CanGIVE), the 4 recommended projects would be implemented in 12 countries, prioritizing support for vaccine delivery and demand generation as part of COVID-19 campaigns and integrated service delivery, including bundling with humanitarian responses as needed. These projects would also enable the strengthening of critical elements of country health systems,

[2] Weekly epidemiological update on COVID-19 - 16 November 2022 (who.int)
[3] COVID-19 pandemic fuels largest continued backslide in vaccinations in three decades (who.int)

GAC - Released under the Access to Informations Act/
AMC - Divulgué en vertu de la loi sur l'accès à l'information

improve preparedness capacities for future pandemics, and support the scale-up of local manufacturing capacity to diversify global vaccine production in the years to come.

Gender Equality and the Empowerment of Women and Girls

Disease outbreaks and pandemics affect women and men differently, and tend to worsen existing gender inequalities, sexual and gender-based violence, as well as discrimination due to increased tensions in the household, economic stress, including unpaid care work and disruption or collapse of systems and structures that protect girls and women. In particular, the COVID-19 pandemic has disproportionately affected women and girls. Furthermore, 70% of healthcare workers are women; women and girls also dominate the social and service sectors globally.

ACT-Accelerator partners have robust strategies and proven track records of working with governments and civil society organizations to dismantle barriers that would prevent women and girls, in all their diversity, and groups experiencing vulnerability from accessing health and immunization services. Global Affairs Canada (GAC) would continue to work with these organizations to ensure the full integration of gender-based analysis and gender equality throughout their strategies and programming efforts. The proposed projects would align with GAC gender equality requirements; additional information is outlined in Annex B.

Expected Results and Implementation

The ACT-Accelerator aligns with GAC's Departmental Results Framework Outcome 3.1: Improved physical, social and economic well-being for the poorest and most vulnerable, particularly for women and girls, in countries where Canada engages. It also aligns with the Feminist International Assistance Policy Health and Nutrition Action Area COVID-19 Logic Model Ultimate Outcome: Decreased COVID-19 related mortality and morbidity of populations, especially for those experiencing marginalization and/or vulnerability.

Specifically, the recommended projects are expected to contribute to increasing equitable access to COVID-19 vaccines, and improving the deployment and delivery of essential health services and COVID-19 medical countermeasures through the key outcomes outlined below:

- Strengthened capacity to deliver COVID-19 vaccines in lower-middle income countries (LMICs).
- Increased ability of manufacturers in LMICs to develop, test, license and produce COVID-19 vaccines.
- Strengthened country capacity to provide primary and community-based services that are critical for the effective delivery of COVID-19 medical countermeasures, as well as essential health services.
- Strengthened country capacity to detect, prevent and respond to health emergencies.

Specific project details and anticipated results are outlined in Annex B.

Risk

Given the unprecedented situation that the world is facing due to the impacts of the COVID-19 pandemic, there are numerous variables that may affect project implementation, including the development, manufacturing, supply and effectiveness of the products, the capacity of downstream recipients, evolving country demands and priorities, among other social factors. While the overall risk level for this programming is low, programming may be affected by weak health systems and decreasing demand for vaccines at the national and subnational levels, resulting in the inability to forecast and sustain COVID-19 efforts effectively and efficiently.

Over the past year, Canada has been working closely with ACT-Accelerator partners, which are long-standing and reliable global health organizations with decades of experience in delivering assistance to LICs and LMICs, as well as a solid track record in financial accountability, risk management and results.
ACT-A partner organizations have significant expertise collaborating with local bodies and operating in difficult contexts with marginalized populations. Given our strong collaboration, coordination and commitment to deliver results, GAC anticipates minimal risks, but plans to mitigate these risks by continuing to work with these leading international authorities and partners, and by retaining flexibility to adjust allocations, within the

GAC - Released under the Access to Informations Act/
AMC - Divulgué en vertu de la loi sur l'accès à l'information

delegated authorities of the Minister of International Development, in response to evolving situational and operational realities.

Individual project risks can be found in Annex B: Summary of Project Descriptions.

Other Considerations

Projects outlined in this memorandum would allow Canada to meet its 200 million dose donation commitment. In line with the 2022 G7 methodology, both physical dose donations and cash equivalents for vaccine-related activities are counted towards G7 dose donation commitments. Dose equivalents are calculated utilising the 2021 OECD rate of $6.72 USD per dose. To date, Canada has donated the equivalent of approximately 190 million doses. Proposed allocations to ACT-Accelerator partners in this memorandum (valued at $117 million in Annex A) translate into an additional 13.89 million dose equivalents, adding up to more than 204.5 million doses provided by Canada, by the end of 2022.

Communications

If approved, the $117 million in funding allocations to CanGIVE partners would provide an opportunity to demonstrate Canada's continued support to the global response to the COVID-19 pandemic, and the fulfilment of its 200-million COVID-19 vaccine dose commitment. The funding could be announced by the Prime Minister or the Minister of International Development, prior to the end of 2022, in the context of a high-level international event, or in a news release to mark the achievement of this goal. There has been a sustained level of media interest in Canada's progress towards meeting this goal. Individual funding allocations to partners would be announced at a later date; for instance, in the context of ministerial trips.

This funding is part of the $732-million commitment to the Access to COVID-19 Tools (ACT) Accelerator announced by the Prime Minister at the Global COVID-19 Summit hosted by the United States on May 12, 2022, while the CanGIVE initiative was announced by the Minister of International Development on June 22, 2022.

Canada's contribution to the projects would be publicly recognized by partners in all of their communications and project activities, including events, website, social media and publications. Information on Canada's contributions, the project descriptions, anticipated results and stories from the field would be posted on GAC's website and social media platforms. Relevant Canadian missions would amplify social media content on the projects on their platforms, as appropriate.

Due Diligence

The department has assessed the past performance, technical, managerial, administrative and financial capacity of the fund recipients listed in Annex A and is satisfied that they are competent and would be able to deliver the intended development results for their projects without impeding upon their capacity to deliver on existing commitments.

☑ **Completed: No concern was raised.**

As part of the due diligence process, these projects have been assessed against contracting and financial management requirements, as well as gender equality and environmental sustainability policies. These projects meet departmental due diligence requirements.

These projects have been assessed against the requirements of subsection 4(1) of the *Official Development Assistance Accountability Act* to ensure that they contribute to poverty reduction, take into account the perspectives of the poor, and are consistent with international human rights standards. Equitable access to life-saving COVID-19 countermeasures, undertaken for, and in collaboration with LICs and LMICs, would help reduce poverty and uphold international human rights. As it complies with the criteria set out in the legislation, it shall be included in the annual report to Parliament as official development assistance.

GAC - Released under the Access to Informations Act/
AMC - Divulgué en vertu de la loi sur l'accès à l'information

Recommendations

It is recommended that you sign below to approve amendments to 3 agreements and enter into 1 agreement for the projects listed below and outlined in detail in Annex B, to be funded through resources from Budget 2022. It is also recommended that you approve extensions to project durations for all 4 projects from 18 to 24 months.

1. **P-011782 COVID-19 Vaccine Delivery and Demand**
   An increase of $60 million to the existing grant arrangement with UNICEF, for a total value of GAC contribution of $170 million.
2. **P-011860 COVID-19 Vaccine Delivery and Demand**
   An increase of $27 million to the existing grant arrangement with the World Health Organization, for a total value of GAC contribution of $57 million.
3. **P-011811 Improving Equitable Access & Vaccination**
   An increase of $15 million to the originally approved project with the Pan-American Health Organization, resulting in a grant arrangement to be signed for a total value of GAC contribution of $45 million.
4. **P-011103 COVID Vaccine Manufacturing Hub**
   An increase of $15 million to the existing grant agreement with the Medicines Patent Pool, for a total value of GAC contribution of $45 million.

Christopher MacLennan
Deputy Minister of International Development

2022-11-23
Date

☑ I AGREE with the recommendation.

☐ I DO NOT AGREE with the recommendation.

Harjit Singh Sajjan
Minister of International Development

2022-11-28
Date

Attachments:

- Annex A: Project Funding Table
- Annex B: Summary of Project Descriptions

GAC - Released under the Access to Informations Act/
AMC - Divulgué en vertu de la loi sur l'accès à l'information

Annex A

Project Funding

(Figures in millions of Canadian dollars)

Projects	Countries	Partner	Signed Project Value	Cost increase	New Total Project Value
**P-011782**<br><br>COVID-19 Vaccine Delivery and Demand	Jamaica, Colombia, Haiti, Democratic Republic of Congo (DRC), Senegal, Nigeria, Côte d'Ivoire, Ghana, Mozambique, Tanzania, Bangladesh	UNICEF	$110 million	$60 million	$170 million
**P-011860**<br><br>COVID-19 Vaccine Delivery and Demand	Democratic Republic of Congo (DRC), Senegal, Tanzania, Nigeria, Côte d'Ivoire, Ghana, Mozambique	WHO	$30 million	$27 million	$57 million
**P-011811**<br><br>Improving Equitable Access & Vaccination	Regional (Americas)	PAHO	$30 million	$15 million	$45 million
**P-011103**<br><br>COVID Vaccine Manufacturing Hub	South Africa	Medicines Patent Pool	$30 million	$15 million	$45 million
**Projects Total**			**$200 million**	**$117 million**	**$317 million**

GAC - Released under the Access to Informations Act/
AMC - Divulgué en vertu de la loi sur l'accès à l'information

Annex B

Summary of Project Description, Changes and Activities, and Expected Results

Project Number: P011782-002
Partner: United Nations Children's Fund (UNICEF)
Title: COVID-19 Vaccination Delivery and Demand
Country/Region: Jamaica, Colombia, Haiti, Democratic Republic of Congo (DRC), Senegal, Nigeria, Côte d'Ivoire, Ghana, Mozambique, Tanzania, Bangladesh
Duration: 2022-2024
Global Affairs Canada Contribution: $170 million in 2022/2023
Partner Contribution: N/A
Gender Equality Policy Marker: GE-01
Environmental Integration Coding: Level 0 – Not Integrated
Action Areas: Human Dignity (Health)

Project Description:
As part of Canada's Global Initiative for Vaccine Equity (CanGIVE), the COVID-19 Vaccine Delivery and Demand project would aim to reduce COVID-19 related mortality and morbidity, particularly amongst vulnerable and priority-use populations, by increasing equitable access to and uptake of COVID-19 vaccines in 11 countries. Through this project, UNICEF would support increased equitable distribution and delivery of COVID-19 vaccines and strengthened gender-sensitive WASH, primary health care and routine immunization services, resulting in greater COVID-19 vaccination coverage for priority and vulnerable groups. The project would also strengthen health systems to engage vulnerable and priority-use populations with gender-equitable COVID-19 vaccination, generating increased demand for COVID-19 vaccines.

Gender Equality Policy Marker: GE-01
The COVID-19 pandemic has further entrenched existing gender disparities and barriers, particularly at the household and community level. Evidence has shown that in some countries, women and other vulnerable priority groups such as the elderly and marginalized communities, such as migrants and internally displaced persons, are disproportionately left behind in COVID-19 vaccine coverage. The project would aim to increase gender-equitable distribution and delivery of COVID-19 vaccines and strengthen gender-responsive routine immunization through: i) community outreach and engagement with women and marginalized groups; ii) amplification of women's and girls' voices to decision-markers and as health champions; iii) systemic integration of women's associations into COVID-19 and routine immunization structures; iv) development of targeted communications and approaches for women; v) development and roll-out of a joint gender checklist and faith-gender network; and vi) facilitation of vaccine delivery to women and increased gender-responsiveness of COVID-19 vaccine monitoring and surveillance data.

Risk:
The risk level associated with the delivery of COVID-19 vaccines is low and include challenges relating to national administration and political commitment, public demand and vaccines timing out or expiring in country. To mitigate these risks UNICEF would apply its own internal mechanisms and strong existing partnerships at country-level, alongside complementary oversight on access to supply, investments and delivery progress provided through the COVAX Facility via the Gavi Board, the AMC Engagement Group, the COVID-19 Vaccination Delivery Support Temporary Steering Committee and the UNICEF-led Funders Forum for Delivery. Challenges to scaling up vaccine roll-out and demand generation may present in countries experiencing humanitarian contexts. To mitigate this, UNICEF would leverage longstanding partnerships with local governments and partners to ensure additional security, logistics and operational planning in areas of operation.

Environmental Integration Coding: Level 0 – Not Integrated
The project was screened as having low to moderate risk. It is recommended that the initiative proceed because the partner organization has adequate processes to ensure that the initiative is not likely to result in significant negative environmental effects.

GAC - Released under the Access to Informations Act/
AMC - Divulgué en vertu de la loi sur l'accès à l'information

Expected Results:

- Increased gender-equitable distribution and delivery of COVID-19 vaccines.
- Strengthened health systems to engage vulnerable and priority populations with gender-equitable COVID-19 vaccination and outreach.

Project Number: P011811-001
Partner: Pan American Health Organization (PAHO)
Title: Improving Equitable Access and Vaccination Coverage against COVID-19
Country/Region: Latin America and the Caribbean
Duration: 2022-2024
Global Affairs Canada Contribution: $45 million in 2022/2023
Partner Contribution: N/A
Gender Equality Policy Marker: GE-01
Environmental Integration Coding: Level 0 – Not Integrated
Action Areas: Human Dignity (Health)

Project Description:
Through this project, the Pan-American Health Organization (PAHO), aims to improve equitable access and vaccination coverage against COVID-19 throughout Latin America and the Caribbean (LAC), with a focus in Colombia, Haiti and Jamaica. In line with Canada's Global Initiative for Vaccine Equity (CanGIVE), activities would seek to increase COVID-19 vaccination rates, including adapting strategies to suit each country's context. The approach would support: i) Strengthened health system staff capacity for vaccination through improved policy, programmatic, regulatory, infrastructure and human resources, including integration of a gender perspective; ii) Improved strategy development, information systems and digital platforms by health systems for COVID-19 vaccine surveillance, including data disaggregated by sex, age and ethnicity; iii) Improved capacity of public health experts to build evidence on COVID-19 vaccination uptake, including gender inequalities and inequities; and iv) Improved capacity of health workers in outreach, risk communication and community engagement, with a focus on gender equality.

Gender Equality Policy Marker: GE-01
This project aims to improve equitable access and vaccination coverage against COVID-19, including a specific line of intervention to strengthen health equity in the context of COVID-19 vaccination, with a focus on gender equality. PAHO would seek to build the capacity of national stakeholders to address the differentiated needs of women; undertake a rights-based approach to promote gender equality and address persistent constructs of gender through health communication efforts; develop and roll-out regional and country level virtual courses and community engagement methodologies on gender, traditional medical practice and health; increase the availability and access to sex, age and ethnicity disaggregated information; establish partnerships with women's advocacy groups; and undertake operational research to understand the barriers to access for differentiated populations.

Risk:
While the overall risk level for this project remains low, some risks may present related to the absorptive and delivery capacity of downstream recipients. Programming may be affected by weak health systems at the national and subnational levels, resulting in the inability to establish, sustain and integrate effectively COVID-19 vaccination capacities while also managing additional crises. A lack of qualified health workers may pose additional challenges to the roll-out of COVID-19 vaccines and demand generation efforts.
To address potential risks, Canada has elected to work with PAHO given their proven track record as a leading global health partner in the Americas with a long history of promoting strong immunization systems, administering vaccines and having well-established regional and country partnerships.

Environmental Integration Coding: Level 0 – Not Integrated
The project was screened as having low to moderate environmental risk or opportunity. It is recommended that the initiative proceed as the partner organization has adequate processes to ensure that the initiative is not likely to cause significant negative environmental effects. Measures would be put in place to ensure compliance with PAHO's environmental processes and requirements.

GAC - Released under the Access to Informations Act/
AMC - Divulgué en vertu de la loi sur l'accès à l'information

Expected Results:

- Increased access to COVID-19 vaccination for populations in situation of vulnerability, including women and girls.
- Increased demand by populations in situation of vulnerability, including women and girls, for COVID-19 vaccines.

Project Number: P011860-002
Partner: World Health Organization (WHO)
Title: COVID-19 Vaccine Delivery and Demand
Country/Region: Democratic Republic of Congo (DRC), Senegal, Tanzania, Nigeria, Cote d'Ivoire, Ghana and Mozambique
Duration: 2022-2023
Global Affairs Canada Contribution: $57 million in 2022/2023
Partner Contribution: N/A
Gender Equality Policy Marker: GE-01
Environmental Integration Coding: Level 0 – Not Integrated
Action Areas: Human Dignity (Health)

Project Description:
As part of Canada's Global Initiative for Vaccine Equity (CanGIVE), this WHO project would scale-up COVID-19 vaccine service delivery among the highest priority groups in Cote d'Ivoire, the Democratic Republic of the Congo, Nigeria, Senegal, as well as Tanzania, Ghana and Mozambique, maintaining flexibility to respond to urgent needs in the African region, as required. The planned activities focus on community engagement and seek to address inequities in service delivery by identifying access barriers and conducting context-specific campaigns. Working with other partners, the WHO would coordinate the operational support and logistics for vaccine delivery including, but not limited to the deployment of mobile teams in order to vaccinate older adults, health workers and people with comorbidities, according to country polities for priority groups. The WHO would also strengthen coordination mechanisms in each country to oversee micro-planning, operational support, vaccine demand and delivery. Additionally, the WHO would integrate COVID-19 vaccination services into primary health care to mainstream access to immunization services.

Gender Equality Policy Marker: GE-01
This project aims to support the equitable distribution of COVID-19 vaccines in accordance with the WHO allocation framework. Evidence has shown that in some countries, women and other at-risk priority groups, such as the elderly and marginalized communities, are disproportionately left behind on COVID-19 vaccine coverage. The WHO would implement gender-responsive vaccine delivery activities based on barrier analyses conducted in each country. The project would take into consideration the specific barriers these groups face to ensure equitable access to vaccines and promote confidence in vaccination among these groups.

Environmental Integration Coding: Level 0 – Not Integrated
The project was screened as having low to moderate environmental risk or opportunity. In this project, the main areas of engagement related to environmental management of biomedical waste and energy were used for cold chains, among others.

Risks
The risk level associated with the delivery of COVID-19 vaccines is low and includes challenges relating to national administration and political commitment, public demand and vaccines timing out or expiring in country. To mitigate these risks WHO would apply its own internal mechanisms and strong existing partnerships at country-level, alongside complementary oversight on access to supply, investments and delivery progress provided through the COVAX Facility. Challenges to scaling up vaccine roll-out and demand generation may present in countries experiencing humanitarian contexts. To mitigate this, WHO would leverage longstanding partnerships with local governments and partners to ensure additional security, logistics and operational planning in areas of operation.

GAC - Released under the Access to Informations Act/
AMC - Divulgué en vertu de la loi sur l'accès à l'information

Expected Results:
With a gender responsive perspective and guided by the WHO gender guidelines for immunization programs (Immunization Agenda 2030, WHO, UNICEF & GAVI, 2021):

- At least 80% of people fully vaccinated (according to national policy) among the selected priority groups defined in each country of focus by end 2023.
- Validated standard microplans available in 80% of the targeted sub-national localities.
- Periodic adjustments of plans and strategies guided by improved data analysis from operational research and assessments.
- Visible and functional partner coordination mechanisms for COVID-19 vaccines rollout established in the target localities, inclusive of Civil Society Organisations and community engagement.

Project Number: P011103-002
Partner: Medicines Patent Pool (MPP)
Title: COVID-19 mRNA Technology Transfer and Manufacturing Hub
Country/Region: Sub-Saharan Africa, Asia
Duration: 2022-2024
Global Affairs Canada Contribution: $45 million over 2022/2023
Partner Contribution: N/A
Gender Equality Policy Marker: GE-01
Environmental Integration Coding: Level 0 – Not Integrated
Action Areas: Human Dignity (Health)

Project Description:

As part of Canada's Global Vaccine Initiative for Vaccine Equity (CanGIVE), the overall goal of this project is to increase regional manufacturing capacity in lower-middle income countries (LMICs) through the establishment of a COVID-19 mRNA Technology Transfer and Manufacturing Hub and network. In July 2021, the mRNA hub project was established by WHO and Medicines Patent Pool (MPP) to address the immediate need for COVID-19 vaccines and the longer-term need to build sustainable, locally owned vaccine production capacity in LMICs. The project is based around a technology transfer "hub"—Afrigen—located in South Africa, which would provide the technology development, training and technology transfer, and "spoke" facilities located in LMICs, which would receive training and technology from the hub to be able to produce and sell health products commercially. While the project would initially focus on a COVID-19 vaccine, it would also build a pipeline of future mRNA vaccine candidates (TB, HIV, etc.).

Gender Equality Policy Marker: GE-01

This project aims to contribute to an inclusive manufacturing sector by ensuring adequate gender responsiveness of selected hubs and spokes. Gender equality would be specifically considered as a criterion in the selection of new spokes and included in benchmarks for staff training. Additionally, the project would provide opportunities for women living in marginalized communities to participate in the manufacturing sector and compete in the marketplace, through proactively targeting those countries in the establishment of the hubs/spokes network, which currently have lacking or limited (biologicals) manufacturing capacity.

Environmental Integration Coding: Level 0 – Not Integrated

The project was screened as having low to moderate risk. It is recommended that the initiative proceed as the partner organization has adequate processes to ensure that the initiative is not likely to result in significant negative environmental effects; measures would be put in place to ensure that the partner process is used.

Risk:

GAC - Released under the Access to Informations Act/
AMC - Divulgué en vertu de la loi sur l'accès à l'information

The COVID-19 mRNA Technology Transfer and Manufacturing Hub is the first of its kind. The risks to project implementation involve potential intellectual property (IP) restrictions on the vaccine candidate, the capacity of “spoke” facilities to uptake the mRNA technology, and long-term sustainability of manufacturing facilities. Medicines Patent Pool is mitigating such risks by: i) Providing expertise in IP management, entering agreements to acquire IP, and inventing around existing patents by using new processes more suitable to production in LMICs; ii) applying a rigorous selection process to the selection of “spoke” facilities to ensure required capacity; and iii) supporting spoke facilities not only with technology transfer, but also bio-manufacturing training, business model development and regulatory strengthening. GAC is further mitigating risks to long-term sustainability through engagement on the Gavi Board, one of the largest purchasers of vaccines globally in order to support the entry of new vaccine manufacturers through a dedicated market shaping strategy.

Expected Results:

- Increased mRNA vaccine production capacity in LMICs.

GAC - Released under the Access to Informations Act/
AMC - Divulgué en vertu de la loi sur l'accès à l'information

Purchase Order: 7438737
Project: *P011103*

GRANT AGREEMENT

BETWEEN

THE DEPARTMENT OF FOREIGN AFFAIRS, TRADE AND DEVELOPMENT

AND

MEDICINES PATENT POOL

COVID-19 MRNA TECHNOLOGY TRANSFER AND MANUFACTURING HUB

GAC - Released under the Access to Informations Act/
AMC - Divulgué en vertu de la loi sur l'accès à l'information

Purchase Order: 7438737
Project: *P011103*

Grant Agreement

**Between**	Her Majesty the Queen in right of Canada ("Her Majesty"), represented by the Minister for International Development acting through the Department of Foreign Affairs, Trade and Development (hereinafter "DFATD" or the "Department")
**And**	Medicines Patent Pool ("the Organization"), a legal entity having its head office/principal place of business at:<br><br>Rue de Varembé 7, fifth floor, 1202 Geneva, Switzerland

(Individually referred to as "Party" and collectively as "the Parties"):

Whereas DFATD wishes to provide a grant (hereinafter referred to as "the Grant") to the Organization, in support of the project entitled "*COVID-19 mRNA Technology Transfer and Manufacturing Hub*" described in detail in Annex A (hereinafter referred to as "the Project");

Whereas the Organization is prepared to receive and administer the Grant for the purposes of the Project and will implement the Project directly or enter into agreement(s) with implementing partner(s), if applicable;

Whereas the objective of this Grant Agreement (hereinafter referred to as "the Agreement") is to set out the details of the transfer and administration of the Grant;

THEREFORE, DFATD and the Organization agree as follows:

1. Purpose of the Project

The purpose of the Project is to increase regional manufacturing capacity in southern Africa through the establishment of a COVID-19 mRNA Technology Transfer and Manufacturing Hub. A complete Project description is attached at Annex A.

2. Terms of Payment

2.1. Upon signature of this Agreement by both Parties, DFATD shall make one single payment in an amount of fifteen million Canadian dollars (CDN $ *15,000,000*) for the purpose of the Project described in Annex A under DFATD's fiscal year 2021-2022 (the "Grant").

2.2. The instalment schedule above and amount set out in Sub-Article 2.1 as well as the Estimated Budget provided in Annex B may be updated.

2.3. DFATD shall deposit the funds in the Organization's bank account, the details of which DFATD has on file. The Organization shall advise DFATD by notice of any change to the banking information prior to DFATD making the payment.

GAC - Released under the Access to Informations Act/
AMC - Divulgué en vertu de la loi sur l'accès à l'information

2.4. Any payment to be made to the Organization is subject to there being an appropriation by the Parliament of Canada for the fiscal year in which the payment is to be made. If DFATD's appropriation is changed by the Parliament or if funds are not available for any other reason, this Grant may be reduced or this Agreement may be terminated.

2.5. The Organization agrees to use the Grant funds and any interest earned on the Grant funds exclusively for the purposes of the Project.

2.6. DFATD may withhold payment or request reimbursement of the Grant, following consultation with the Organization should DFATD reasonably determine that the Organization:

a) failed to use the Grant for the purpose of the Project as described in Annex A;

b) receives a payment made by error; or

c) is no longer eligible for the Grant.

3. Administration and Reporting

3.1. All accounts and financial statements shall be denominated in Canadian dollars.

3.2. The Organization shall provide DFATD with the following reports:

3.2.1. An annual narrative report on results, within three (3) months of the end of each calendar year, that provides an evidence-based assessment of progress toward the achievement of intended outputs, results and impacts. This annual narrative report on results should provide evidence of performance using actual data on performance indicators, results and impacts as described in the relevant results framework for this Project, in comparison to baseline data and targets.

The annual narrative report on results should be accompanied by an annex providing actual data on each performance indicator as per its collection frequency.

The annual narrative report on results shall include the following components:

a) an analysis of the key challenges and constraints internal and external to the Organization which may influence the success of the Project as a whole, as well as explanations of variance between expected and actual results, lessons learned and mitigation measures. The Organization will also describe its efforts to ensure sustainability of results achieved and specify whether the Project is on track to achieve the expected outputs, results and impacts;

b) if relevant, an assessment of how the Organization's gender equality strategy, if any, is being implemented by the Project and how it is contributing to the achievement of expected Project results;

GAC - Released under the Access to Informations Act/
AMC - Divulgué en vertu de la loi sur l'accès à l'information

c) if relevant, a summary of environmental issues that affect or arise from the Project and how they are being addressed to ensure expected results are achieved;

d) if relevant, a summary of what governance and human rights issues affect the Project and a description of how governance considerations (i.e. capacity and responsiveness; effectiveness and efficiency; transparency and accountability; equity, equality and non discrimination; participation and inclusion) have been taken into consideration to address the issues in the design, implementation, results and monitoring of the Project.

3.2.2. If relevant, a final narrative report within three (3) months of the of the Project, or in the event of termination of this Agreement, after termination. The report will include an evidence-based analysis of the cumulative outputs, results and impacts achieved over the course of the Project. This final narrative report should provide evidence of performance using actual data on output, outcome and impact level performance indicators, identified in the relevant results framework for this Project, in comparison to baseline data and targets.

The final narrative report should also be accompanied by an annex providing actual data on each performance indicator as per its collection frequency. The report should also outline challenges and lessons learned, and include a summary of considerations identified above in subsections 3.2 b), c), d) above, and how the project is ensuring the sustainability of results achieved.

3.2.3. At DFATD's request and expense, in consultation with the Organization, more frequent reports.

3.2.4. Sub-Article 3.2 above shall survive the expiration or early termination of this Agreement.

4. Monitoring and Evaluation

4.1. The Organization shall permit or cause to be permitted any authorized representative of the Department access to the site(s) of the Project to inspect and assess the progress of the Project and shall supply upon request such data as the Department may reasonably require for monitoring and evaluation purposes. The Department shall keep the Organization informed with respect to the results of such inspections and assessments. The Department reserves the right to proceed with a follow-up review, whenever it deems it necessary, during the implementation of the Project and for three (3) years following the expiry or early termination of the Agreement. The Organization shall maintain and keep in a secure place during this period relevant original records and documents in support of the use of the Grant.

GAC - Released under the Access to Informations Act/
AMC - Divulgué en vertu de la loi sur l'accès à l'information

5. Auditing

5.1. The Grant shall be subject exclusively to the provisions on internal and external audit provided for in the Organization's regulations and rules. The Organization shall inform DFATD of the publication of external audit reports and shall provide a copy to DFATD upon request.

5.2. The Organization shall make these records and all other information necessary to ensure compliance with the terms and conditions of the Agreement, including those in the possession of third parties, available for audit and examination by the Department, the Auditor General of Canada or their respective authorized representatives, as per subsection 7.1 of the *Auditor General Act*. The Organization shall accord, at its own expense, the Department, the Auditor General of Canada or their respective authorized representatives, the proper facilities required for such an audit. The Organization's expenses associated with an audit are not recoverable from the Department.

6. Notices and Communications

6.1. Any notice or communication shall mention the title of the Project and the Project number.

6.2. Any notice given pursuant to the Agreement shall be effectively given if delivered, sent by registered letter or by facsimile to the Organization at the address mentioned below. Any change to the address may be made by notice in accordance with this Article.

6.3. Any communication given pursuant to the Agreement shall be effectively given if delivered, or sent by registered letter, mail, facsimile or email to the Organization at the address mentioned below.

To the Department of Foreign Affairs, Trade and Development:

Name: Megan Cain
Title: Director
Division: Global Health and Nutrition Platforms
Branch: Multilateral and Global Programs Branch
Telephone: 343-203-6096
Address: 111 Sussex Dr, Ottawa, Ontario, K1A 1J1
Email: megan.cain@international.gc.ca

s.19(1)

GAC - Released under the Access to Informations Act/
AMC - Divulgué en vertu de la loi sur l'accès à l'information

Grant Agreement

Purchase Order: 7438737
Project: *P011103*

To the Organization:

Name: Charles Gore
Title: Executive Director
Division: Medicines Patent Pool
Telephone: +41 (0)22 533 50 50
Address: Rue de Varembé 7, fifth floor, 1202 Geneva, Switzerland
Email: [redacted]

7. Duration of the Agreement

7.1. This Agreement shall come into effect upon the date of the last signature and shall expire on March 31, 2024.

8. Suspension and Termination

8.1. DFATD may at any time, by notice in writing, suspend or terminate the Agreement in whole or in part.

8.2. Where applicable, within thirty (30) days prior to the termination date, the Organization shall consult DFATD with respect to the reallocation of any uncommitted balance of the Grant funding.

9. Sexual Misconduct

9.1. Recognizing that sexual exploitation and abuse infringe universally recognized international legal standards, and in accordance with Canada's commitment to provide international aid based on a human rights framework, the Organization declares and guarantees that it has in place, and will maintain for the entire duration of the Agreement, a Code of Conduct available to the public that aims to prevent sexual exploitation and abuse, to investigate and intervene if such a situation arises.

9.2. The Code of Conduct shall be integrated into the activities of the Organization and shall, at minimum, include the following provisions:

9.2.1. An integrated accountability process across the Organization, including roles and responsibilities to ensure oversight and compliance with the Code of Conduct;

9.2.2. An anonymous and confidential reporting mechanism and fair and confidential investigative procedures to address all allegations of sexual exploitation and abuse;

9.2.3. Training on the prevention of sexual exploitation and abuse and corrective measures to be taken in the event of proven misconduct;

9.2.4. Action to be taken, including disciplinary measures, in the event of serious misconduct.

GAC - Released under the Access to Informations Act/
AMC - Divulgué en vertu de la loi sur l'accès à l'information

9.2.5. The Organization shall provide a copy of its Code of Conduct to all personnel, to Local Partners and Ultimate Recipients and promotes protection against sexual exploitation and abuse. The Organization shall ensure that Local Partners and Ultimate Recipients sign an attestation declaring their compliance with the Organization's Code of Conduct, or adopt their own policies and procedures to prevent sexual exploitation and abuse that are consistent with the goals and objectives of the Organization's Code of Conduct.

9.2.6. For the purposes of Article 9, the following definitions apply:

a) Sexual abuse: any sexual assault or threat of sexual assault committed with force coercion, or in the course of an unequal relationship. Any sexual activity with a child is considered to be sexual abuse.

b) Sexual exploitation: any actual or attempted abuse of a position of vulnerability, differential power, or trust, for sexual purposes, including, but not limited to, profiting monetarily, socially or politically.

c) Protection from sexual exploitation and abuse (PSEA): a term used by the United Nations and the non-governmental organization community that refers to action taken to protect vulnerable persons from sexual exploitation and abuse by their own employees and associated personnel.

10. Gender Equality

10.1. In line with Global Affairs Canada's Policy on Gender Equality (https://international.gc.ca/world-monde/funding-financement/policy-politique.aspx?lang=eng), the Organization shall explicitly and systematically implement the gender equality commitments identified in the Agreement, and any other gender equality considerations derived from subsequent gender equality analysis, at all stages of the Project.

10.2. As part of its regular reporting, the Organization shall report to the Department on the application of the above provisions.

11. Amendments

11.1. This Agreement may only be amended before its expiry or early termination. Any amendments shall be made in writing and signed and dated by DFATD and the Organization.

11.2. Without proceeding by way of an amendment as prescribed by Sub-Article 11.1, and subject to DFATD's prior approval, the Parties may amend this Agreement, before the expiry or early termination of this Agreement, in the following situations, documented through a communication, with acknowledgement and acceptance by DFATD and the Organisation:

GAC - Released under the Access to Informations Act/
AMC - Divulgué en vertu de la loi sur l'accès à l'information

a) any change to the instalments schedule when made within the approved Grant amount and budget;

b) any change to the Estimated Budget provided in Annex B;

c) no cost extension of the expiry date of this Agreement;

d) any change to Annex A – Description of the Project, when there is no change in the scope and nature of the Project as determined by DFATD;

e) change of administrative nature, as determined by DFATD which includes those that are intended to improve the delivery of the Project without limiting or affecting its scope, results, duration or value.

12. Indemnification

12.1. The Organization shall, both during and following the termination or expiry of the Agreement, save harmless and indemnify Her Majesty, her employees and her agents from and against all claims, losses, damages, costs and expenses or actions or other proceedings made against them in any manner, attributable to any injury, death, damage to or loss of property arising or alleged to arise from the execution of the Project, except to the extent that the injury, death, damage or loss has been caused by the negligence of Her Majesty, her employees or agents.

13. Public Office Holder, Member of the Canadian House of Commons, Member of the Senate, Public Servant

13.1. No current or former public office holder, member of the Canadian House of Commons, member of the Senate, current or former public servant of the Government of Canada who is not in compliance with the Canadian Conflict of Interest Act, 2006, c. 9, s.2, the Conflict of Interest Code for Members of the House of Commons, the Conflict of interest Code for Senators, the Values and Ethics Code for the Public Service and the Values and Ethics Code for the Public Sector shall derive a direct benefit from this Agreement unless the provision or receipt of such benefit is in compliance with such legislation and codes.

14. Lobbying Activities

14.1. The Organization declares and guarantees that any person lobbying on its behalf, as defined in the Lobbying Act of Canada, R.S.C., 1985, c. 33 [4th Supp.], is compliant with that act.

15. Public Recognition

15.1. Acknowledgement of Grant

At no additional cost to DFATD, the Organization agrees to acknowledge DFATD's funding in any public reference to the Project such as but not limited to

GAC - Released under the Access to Informations Act/
AMC - Divulgué en vertu de la loi sur l'accès à l'information

announcements, interviews, speeches, press releases, publications, signage, websites, promotional materials and advertising, ensuring the appropriateness and accuracy of any messages. The Organization agrees to use any available opportunities to demonstrate that the Project is funded in whole or in part by the Canadian Government through DFATD.

After consultation, DFATD or the Organization may request to cease all public recognition activities inter alia for security, programming or other compelling reasons. DFATD and the Organization will consult each other to determine when the public recognition activities may resume.

15.2. Announcements

DFATD shall be informed, with reasonable advance notice, of any major public announcement relating to activities funded under this Agreement, to give the representative of the Government of Canada the opportunity to be involved in making the announcement. Where DFATD has expressed the desire to become involved, the Organization shall cooperate with DFATD in making the announcement.

15.3. Public materials

All public materials issued jointly by the DFATD and the Organization must be judged acceptable by both Parties and shall be made available in both English and French.

16. Declarations and Guarantees

16.1. Fraud and Corruption

16.1.1. The Organization acknowledges that it has rules and policies to adequately implement internal controls, in regards to anti-corruption, anti-fraud, anti-bribery and other situations of misuse of funds.

16.1.2. As signatory of the Organisation for Economic Co-operation and Development (OECD) Convention on Combating Bribery of Foreign Public Officials in International Business Transactions (1997), and the United Nations Convention Against Corruption (UNCAC, 2007), the Government of Canada is committed to the fight against corruption, fraud and bribery. Therefore, DFATD reserves the right to take any appropriate action to address such practices, including recovering resources lost, suspending or terminating the Agreement.

16.1.3. The Organization declares and guarantees that no offer, gift or payment, consideration or benefit of any kind, which constitutes an illegal or corrupt practice, has been or will be made to anyone by the Organization, either directly or indirectly, as an inducement or reward for the award or execution of the Agreement.

GAC - Released under the Access to Informations Act/
AMC - Divulgué en vertu de la loi sur l'accès à l'information

16.1.4. The Organization declares and guarantees that neither the Organization, nor its employees involved in the Project:

a) were convicted during a period of three (3) years prior to and since the submission of the Project proposal, by a court of law in Canada or in any other jurisdiction for an offence involving fraud, bribery or corruption or;

b) are under sanction, for an offence involving fraud, bribery or corruption, imposed by a government, an international governmental organization or an organization providing development assistance

16.1.5. The Organization declares and guarantees that it has taken all reasonable steps to assure itself that neither its local partners nor its subcontractors or sub-recipients, nor its local partners' or subcontractors' and sub-recipients' employees involved in the Project:

a) were convicted during a period of three (3) years prior to and since the submission of the Project proposal, by a court of law in Canada or in any other jurisdiction for an offence involving fraud, bribery or corruption or;

b) are under sanction, for an offence involving fraud, bribery or corruption, imposed by a government, an international governmental organization or an organization providing development assistance.

16.1.6. The Organization shall notify DFATD immediately of any allegation or actual case of misuse of funds fraud, bribery, corruption or financial irregularity which may involve DFATD's funding or which could put DFATD's funding at risk. The Organization shall immediately provide electronically to allegations@international.gc.ca with a copy to DFATD's representative identified in the Agreement, a written summary of such event including a description of the event, the amount involved, the actions taken or to be taken by the Organization to resolve the issue and any additional information that will assist DFATD in its determination of the way forward.

16.2. Anti-Terrorism

16.2.1. The Organization declares and guarantees that the funding for the purposes of the Project shall not knowingly be used to benefit terrorist groups or individual members of those groups, or for terrorist activities, either directly or indirectly, as defined in the Criminal Code R.S.C., 1985, c. C-46 or appearing on the Consolidated United Nations Security Council Sanctions List as modified during the term of this Agreement. The Organization shall notify in writing DFATD immediately if it is unable to complete the Project as a result of terrorism-related concerns.

16.2.2. The Organization is responsible for consulting all relevant lists in order to stay informed of the listed terrorist groups and their members and must ensure that the

GAC - Released under the Access to Informations Act/
AMC - Divulgué en vertu de la loi sur l'accès à l'information

Grant of DFATD does not benefit any listed terrorist entity and their members, any sanctioned groups or persons. Entities or individuals listed as terrorists can be found at the following web addresses:

a) Criminal Code of Canada list.

b) Regulations Implementing the United Nations Resolutions on the Suppression of Terrorism (RIUNRST)

c) The United Nations Security Council Consolidated Sanctions List is available on the United Nations Security Council website (https://www.un.org/securitycouncil/) to implement the sanction measures imposed by the United Nations Security Council pursuant to resolutions 1267 (1999), 1989 (2011) and 2253 (2015) concerning ISIL (Da'esh), Al-Qaida, and associated individuals, groups, undertakings and entities, and pursuant to resolution 1988 (2011) concerning the Taliban and associated individuals.

The Organization is responsible for consulting the aforementioned lists even in the event that the provided web addresses are no longer valid.

16.2.3. For specific Project(s), DFATD may request the Organization, in addition to the above:

a) to provide to DFATD the names of its executive officers and members of the board of directors, as well as a list of its implementing partner(s) and their executive officers and members of the board of directors, before the signature of this Agreement. If not received before signature, the Organization shall provide to DFATD the names and list as soon as they are available;

b) to notify DFATD if any implementing partner(s) change or if they are aware of any changes in those holding the positions of board members, directors or executive officers within the Organization or any subcontractor. Any change to name and list shall be submitted to the Department in writing, no later than thirty (30) days before signing any agreement with the proposed new implementing partner(s).

16.2.4. DFATD may inform the Organization in writing if it has identified implementing partner(s) that are associated directly or indirectly with terrorism. In such instance, DFATD will, in consultation with the Organization, determine an appropriate course of action, including suspension or termination of this Agreement.

16.2.5. The Organization shall include a corresponding provision in any subcontract or sub-agreement that the Organization enters into for the purposes of the Project.

16.3. Economic Sanctions and Other Trade Controls

GAC - Released under the Access to Informations Act/
AMC - Divulgué en vertu de la loi sur l'accès à l'information

16.3.1. The Organization declares and guarantees that funding for the purposes of the Project will not be knowingly used, either directly or indirectly, in a manner that contravenes economic sanctions imposed by Canada and enforced by regulations under the United Nations Act (R.S.C. (1985), c. U-2); the Special Economic Measures Act (S.C. (1992), c. 17); the Justice for Victims of Corrupt Foreign Officials Act (S.C. (2017), c. 21) as they are amended from time to time, or for activities that would contravene the provisions of the Export and Import Permits Act (R.S.C. (1985), c. E-19). Information on Canadian sanctions and export and import controls can be found at the following links:

a) https://www.international.gc.ca/world-monde/international_relations-relations_internationales/sanctions/index.aspx?lang=eng

b) https://www.international.gc.ca/world-monde/international_relations-relations_internationales/sanctions/types.aspx?lang=eng

c) https://www.international.gc.ca/controls-controles/index.aspx?lang=eng

16.3.2. The Organization shall consult the above links to be aware of the foreign governments, persons and activities subject to economic sanctions and other trade controls during the term of this Agreement.

16.3.3. The Organization shall comply with the legislations and regulations related to economic sanctions and other trade controls, and with any modifications made to them, during the term of this Agreement.

16.3.4. The Organization shall include a corresponding provision in all subcontracts and sub-Agreements it signs for the purposes of the Project.

16.4. The Organization shall notify in writing the Department immediately if it is unable to complete the Project as a result of any issue identified in Article 16. The Organization agrees that if it does not comply with this Article 13, the Department will determine an appropriate course of action, including the suspension or termination of this Agreement.

17. Environmental Assessment

17.1. This Agreement may involve the carrying out of one or more components that is likely to cause negative environmental effects. The Organization shall ensure that the project is not likely to cause significant adverse environmental effects. The Organization shall ensure that the management of environmental effects, including any analysis, is carried out in accordance with the environmental processes and requirements of the Organization.

17.2. Upon DFATD's request, the Organization shall provide DFATD with a copy of any environmental analysis(-es) and any supporting documentation.

GAC - Released under the Access to Informations Act/
AMC - Divulgué en vertu de la loi sur l'accès à l'information

17.3. DFATD may also evaluate whether the management of environmental effects was carried out in accordance with the environmental process(-es) and requirements of the Organization.

17.4. The Organization shall explicitly and systematically implement environmental considerations identified in the Agreement, and in any subsequent environmental analysis, at all stages of the project.

17.5. As part of its regular reporting, the Organization shall report to DFATD on the application of the above provisions.

18. Official Languages

18.1. **Public recognition**

18.1.1. All public information materials issued jointly by DFATD and the recipient must be considered acceptable by both parties and be available in both of Canada's official languages.

18.1.2. In consultation with DFATD, the recipient uses every opportunity available to ensure the visibility and recognition of Canada's contribution to the Project in publications, speeches, press releases, websites, social media or other communications material in accordance with the Federal Identity Program which can be consulted at: https://www.international.gc.ca/world-monde/funding-financement/public_visibility_recognition-visibilite_reconnaissance_publique.aspx?lang=eng.

18.2. **Information about the Project**

18.2.1. The recipient ensures that basic information about the Project is available in both of Canada's official languages.

18.3. **Project activities**

18.3.1. The recipient ensures that the Project's workshops are conducted in English and in French, and that official language minority communities in Canada are invited to participate. To do so, the recipient, as required, ensures that the content of the documents is available in English and in French.

18.3.2. The recipient ensures that all publications, communications and/or services provided in the context of the Project for specific group targeted by the project are provided in both official languages.

18.3.3. The recipient ensures that its staff is able to communicate and assess funding applications in both of Canada's official languages.

GAC - Released under the Access to Informations Act/
AMC - Divulgué en vertu de la loi sur l'accès à l'information

18.4. **Promotion**

18.4.1. The recipient takes the official language minority community into consideration in the planning and implementation of its activities, and makes reasonable efforts to promote both official languages, as applicable.

19. No Employee or Agency Relationship

19.1. Nothing in this Agreement has the effect of creating a partnership, joint venture, agency or employment relationship between the Parties. The Organization agrees that neither the Organization nor any of its employees, agents, subcontractors or sub-recipients shall represent themselves in any manner to be employees, agents, or partners of Her Majesty or DFATD.

20. Total Funding

20.1. The Organization declares that all sources of funding for the Project with their corresponding amounts have been disclosed in writing to the DFATD. Should new funding from other sources for the Project become available during the Agreement, the Organization shall disclose and update this information in writing in its reports to DFATD. Where the total amount of funding from all sources exceeds one hundred percent (100%) of the value of the Project, DFATD reserves the right to adjust the Grant or recover any surplus paid, up to the amount of the Grant made under the Agreement.

21. Intellectual Property

21.1. All intellectual property rights are vested in the Organization.

21.2. The Organization hereby grants Her Majesty and DFATD the right to inter alia use, reproduce, adapt, translate, publish, disseminate and distribute the Organization's reports prepared by the Organization and shared with DFATD for non-commercial purposes and free of charge.

21.3. The obligations contained in this Article must be reproduced in all sub-agreements and subcontracts.

22. Access to Information and Confidential Information

22.1. Access to Information

All information provided by the Organization shall be treated in accordance with the Access to Information Act and the Privacy Act. DFATD may compile and publish statistics based on information contained in the Agreement and arising from its performance. DFATD may publish the Organization's name and address, the amount of the Grant, the type of activities funded, the title of the Project and the name of the recipient country.

22.2. Confidential Information

GAC - Released under the Access to Informations Act/
AMC - Divulgué en vertu de la loi sur l'accès à l'information

The Organization shall not disclose any confidential information or documents or make use of any intellectual property rights subject matter that it becomes aware of or takes possession of during the implementation of the Project, without having obtained written authorization from the appropriate authority that can release it from the obligation to confidentiality. Upon DFATD's request, the Organization shall provide the Department with a copy of the approval obtained.

23. Procurement and Disposal of Assets

23.1. The Organization shall be responsible and accountable for the procurement of goods, equipment and services for the Project and shall respect the principles of transparency, integrity, competition, fairness and value for money. The Organization shall maintain procurement files containing the relevant procurement documentation.

23.2. Assets acquired with the Grant are to be used for the purposes of the Project and it is the responsibility of the Organization to take proper care and ensure the security of such assets.

24. Contractual Commitments with Third Parties

24.1. DFATD shall not be held liable for any loans, leases, capital leases or any other contractual commitments entered into by the Organization with any third party for the implementation of the Project.

25. Dispute Resolution

25.1. The Parties agree to attempt to resolve any dispute through negotiation or through other mutually agreed appropriate alternate resolution process.

25.2. Notwithstanding Sub-Article 25.1, the Parties maintain the right to seek legal recourse through a court having relevant jurisdiction.

26. Applicable Law and Jurisdiction

26.1. This Agreement shall be governed by, and is to be construed and interpreted in accordance with, the laws in force in *Ontario, Canada*, and the laws of Canada applicable therein. The Parties - acknowledge the exclusive jurisdiction of the courts and tribunals of the province of *Ontario, Canada*.

27. Notice of Completion of Activities, Assignment and Successors

27.1. The Organization shall notify DFATD when all activities relating to the Project have been completed.

27.2. This Agreement may not be assigned by the Organization without the prior written consent of DFATD.

27.3. The Agreement is binding on the Parties and their successors and permitted assigns.

s.19(1)

GAC - Released under the Access to Informations Act/
AMC - Divulgué en vertu de la loi sur l'accès à l'information

28. Entire Understanding

28.1. This Agreement, together with Annex A and B, which forms an integral part of the Agreement, constitute the entire understanding between DFATD and the Organization with respect to the Project.

29. Signatures

29.1. This Agreement is written in English in two copies. The Parties can sign this Agreement without the other Party being present. Each copy signed by the Parties shall be an original.

29.2. This Agreement has been signed for Her Majesty and for the Organization by their respective representatives, duly authorized.

Medicines Patent Pool	Her Majesty
[redacted]	Hisko, Mellissa Digitally signed by Hisko, Mellissa Date: 2022.03.18 10:14:40 -04'00'
Signature	*Signature*
Charles Gore	Mellissa Hisko
Name	Name
Executive Director	Acting Director, COVID-19 Response Taskforce
Title	Title
18th March 2022	March 18, 2022
Date	Date

GAC - Released under the Access to Informations Act/
AMC - Divulgué en vertu de la loi sur l'accès à l'information

Purchase Order: 7438737
Project: *P011103*

Annex A – Project Description

1. PROJECT DESCRIPTION

1.1 Canada is providing $15 million to the Medicines Patent Pool to support the COVID-19 Manufacturing and Technology Transfer Hub project, undertaken by COVAX.

1.2 Medicines Patent Pool is a COVAX partner. Working through Medicines Patent Pool, this funding supports COVAX partners to: i) establish a technology transfer hub for mRNA COVID-19 vaccines in South Africa, ii) facilitate technology transfer to recipient manufacturing facility/facilities, and iii) establish vaccine production by the recipient facility/facilities. This project is part of Canada's commitment to the ACT-Accelerator.

1.3 The full scope of the five-year initiative (total budget of roughly CAD$130 million) is to establish voluntary technology transfer to several recipient facilities in Africa, Latin America and Asia. Canada's $15 million investment supports the initial phase of the project over the first two years. During this period, Canada's funding will support: the establishment of the technology transfer hub itself (Afrigen Biologics); transfer technology of a locally developed mRNA vaccine to a network of recipient facilities, the first of which is the Biovac Institute; and exploration into the application of mRNA technologies beyond COVID-19 for other infectious diseases such as malaria, HIV and tuberculosis, which will be conducted in partnership with the South African Medical Research Council.

2. EXPECTED RESULTS

2.1 The overall aim of this project is to increase regional manufacturing capacity in southern Africa through the establishment of a COVID-19 mRNA Technology Transfer and Manufacturing Hub.

2.2 The project will support increased regional availability of mRNA vaccines for COVID-19 and explore the broader application of mRNA technologies towards additional infectious diseases. The goal is to achieve ultimate production at a first facility reaching 50-100[1] million doses per year for the southern African region.

2.3 Reporting will be provided annually and produced in coordination of the World Health Organization. Regular updates will also be provided via the mRNA Hub Steering Committee Funders Forum.

[1] To be further refined as additional facilities in the mRNA network begin production, demand for COVID-19 vaccines becomes clearer and mRNA vaccine development for additional diseases is tested.

GAC - Released under the Access to Informations Act/
AMC - Divulgué en vertu de la loi sur l'accès à l'information

3. PROJECT ACTIVITIES

3.1 Canada's funding will primarily support the establishment of the Technology Transfer Hub, through activities such as:

- Preparation and start-up of the Hub facilities;
- Training of staff and technical assistance
- Receipt and/or development of mRNA vaccine technology;
- Review and negotiation of intellectual property agreements and regulatory processes;
- Conducting of research and development activities, including preclinical and clinical trials;
- Design of the technology transfer program;
- Maintenance of the Hub facilities; and
- Other miscellaneous activities, as required

3.2 To the extent possible, Canada's funding will also support the preparation of recipient manufacturing facilities, with activities including, but not limited to:

- Training of staff in mRNA technology;
- Preparation of facilities to receive mRNA technology;
- Transfer of technology;
- Phase 3 trials on vaccine candidate(s);
- Scale-up of vaccine manufacturing;
- Collaboration with academic partners to explore broader application of mRNA technologies; and
- Other miscellaneous activities, as required.

GAC - Released under the Access to Informations Act/
AMC - Divulgué en vertu de la loi sur l'accès à l'information

Grant Agreement

Purchase Order: 7438737
Project: *P011103*

ANNEX B – Estimated Budget for the Project

[redacted]

[redacted]

Canada

s.20(1)(c)

GAC - Released under the Access to Informations Act/
AMC - Divulgué en vertu de la loi sur l'accès à l'information

Grant Agreement

Purchase Order: 7438737
Project: *P011103*

[redacted]

GAC - Released under the Access to Informations Act/
AMC - Divulgué en vertu de la loi sur l'accès à l'information

Schulz, Sara -MNV [She,Her | Elle]

From: [redacted]@oecd.org
Sent: April 4, 2022 3:35 AM
To: Drolet, Hubert -SWS; Schulz, Sara -FMNV; Guiet, Jeremie -SWS
Cc: Mamhikoff, Maher -SWS; [redacted]@oecd.org
Subject: RE: Quick Summary of Mtg re: ODA Eligibility and P011103 mRNA Manufacturing Hub

Dear all,

Thank you for sharing the description of the project and for talking me through it during our call last week. I could discuss with [redacted] and can confirm that in our view **the project described is indeed ODA-eligible.**

The objective of the manufacturing hub is to increase mRNA vaccine production capacity in low and middle-income countries (under-served regions), which will promote regional health security. The focus is on support to manufacturers in low and middle-income countries to produce their own vaccines (South Africa, Argentina and Brazil are all ODA-eligible countries; Bangladesh, Indonesia, Pakistan, Serbia and Vietnam as well). Moreover, the vaccine is expected to be more suited to the geographical contexts (e.g. fewer storage constraints) and the project will also explore the possibly to apply the mRNA technology to other infectious diseases, hence binging longer term benefits to the regions concerned.
The concern that the WP-STAT earlier discussed in relation to donors' funding of R&D for COVID-19 vaccines was that the development of such a vaccine would be a global public good, benefitting also in large part to donor countries themselves. However, the Manufacturing hub project is focused on the problems of developing countries, it aims at transferring a technology (mRNA) to developing countries, supporting their manufacturing facilities and building their human capacity. This confirms that the project "is administered with the promotion of the economic development and welfare of developing countries as its main objective" and it is therefore deemed ODA-eligible.
The funds will support the private sector in the developing countries concerned (pharmaceutical and biotechnology companies). As mentioned during the call, there is a review this year of the reporting rules for this type of support which will look at the accounting methodology of instruments such as equities or loans to the private sector (grant equivalent vs cash-flows). Our understanding is that Canada's funding to the Hub will be in the form of a grant, for which the basis of reporting will in any case remain the same.

Best regards, [redacted]

From: Hubert.Drolet@international.gc.ca <Hubert.Drolet@international.gc.ca>
Sent: 28 March, 2022 9:05 PM
To: Sara.Schulz@international.gc.ca; GUIET Jeremie [Canada] <jeremie.guiet@international.gc.ca>
Cc: MAMHIKOFF Maher [Canada] <maher.mamhikoff@international.gc.ca>; [redacted] DCD/FSD <[redacted]@oecd.org>
Subject: Quick Summary of Mtg re: ODA Eligibility and P011103 mRNA Manufacturing Hub

Good afternoon Sara,

This is simply to quickly document the outcome of this morning's "due diligence" meeting with the OECD's Secretariat and eligibility of P011103 in ODA. In attendance were

Sara Schulz – GAC / FMNV Program /Senior Analyst / GAC Covid Task Force
Hubert Drolet – GAC /SWS Deputy Director / Intl. Dev. Stats /
Jeremie Guiet -- GAC /SWS Senior Analyst / Intl. Dev. Stats /
[redacted] -- OECD-[redacted]/Development Cooperation Division (DCD)

GAC - Released under the Access to Informations Act/
AMC - Divulgué en vertu de la loi sur l'accès à l'information

- Schulz/FMNV quickly summarized the contents of the documentation provided to the Secretariat in order to assist with the ODA eligibility assessment.
- [redacted] of the OECD confirmed that she had read the documentation provided and concluded that the project falls well within the ODA definition set out in the Reporting Directives.

- In her appraisal, she considered whether developing countries were the main beneficiaries. Broadly speaking there was no uncertainty regarding the overall mandate/objective of supporting LDCs, and it was clearly within the purview of ODA; capacity building and technical assistance/transfer are clearly ODA-able.
- She stated having initial reservations regarding the research component, but was reassured by the clarity in the design documents, which clearly stated that the research would be conducted in developing countries, with the support of local and regional research organizations/councils.
- Another aspect which was discussed was the potential issue of supporting donors' own private sector organizations in ODA. [redacted] explained that this could be a future eligibility issue being discussed in the year. Essentially, supporting donor's own private sector could crowd out regional investments, and would not meet ODA criteria of additionality. On this point, Schulz/FMNV explained that it would be NOT the case in this initiative; the grants support existing established organizations in developing countries, who are scaling up much needed technical expertise. Here, Canada may need to exercise care to ensure investments continues to be additional in character.

- Schulz/FMNV asked whether the DCD could provide written confirmation of the discussion. [redacted] agreed to provide such confirmation, after quickly confirming with the manager of her unit later this week,.

Trust this helps,

----
a -FMNV
022 10:37 AM
rt -SWS
ie -SWS
DA Eligibility - P011103 mRNA Manufacturing Hub

I'm available Monday morning except for 10-11am EST. Would be happy to join, thanks for coordination Hubert!

Sara

From: [redacted]@oecd.org <[redacted]@oecd.org>
Sent: March 25, 2022 10:31 AM
To: Drolet, Hubert -SWS <Hubert.Drolet@international.gc.ca>
Cc: Schulz, Sara -FMNV <Sara.Schulz@international.gc.ca>; Guiet, Jeremie -SWS <jeremie.guiet@international.gc.ca>
Subject: RE: ODA Eligibility - P011103 mRNA Manufacturing Hub

Dear Hubert and Jeremie,

s.19(1)

s.21(1)(b)

GAC - Released under the Access to Informations Act/
AMC - Divulgué en vertu de la loi sur l'accès à l'information

I did not forget, but these days are really busy! It is a good idea to organise a call early next week (Monday and Tuesday afternoons work on my side). Thanks and best regards, [redacted]

From: Hubert.Drolet@international.gc.ca <Hubert.Drolet@international.gc.ca>
Sent: 25 March, 2022 1:12 PM
To: [redacted] DCD/FSD <[redacted]@oecd.org>
Cc: Sara.Schulz@international.gc.ca
Subject: RE: ODA Eligibility - P011103 mRNA Manufacturing Hub
Importance: High

[redacted]

I tried cold calling you over Teams/Skype this morning, but wasn't able to reach you.

Apologies for pressing, as I suspect there is a lot on your plate to tidy up since last weeks meetings.

Is there a small chance you were able to look at the documents I shared with you earlier this week? The officers in charge would like to know if we could get an indication of ODA eligibility by early next week, since it may factor into budget decisions on approval. My sense is that this initiative is clearly focused on vaccine equity in ODA-eligible countries, including but not limited to Covid-19. [redacted] this initiative seems to be more clearly aligned with specific issue of vaccine manufacturing in the south.

Could we organize a call early next week to discuss with my colleague copied above? Many thanks and best regards,

Hubert

On Mar 24, 2022, at 1:29 PM, Drolet, Hubert -SWS <hubert.drolet@international.gc.ca> wrote:

GAC - Released under the Access to Informations Act/
AMC - Divulgué en vertu de la loi sur l'accès à l'information

Hi Sara, I have not received a response. Planning to follow up with a phone call tomorrow am to get an update. Sorry for the delay.

From: Schulz, Sara -FMNV <Sara.Schulz@international.gc.ca>
Sent: March 24, 2022 1:21 PM
To: Drolet, Hubert -SWS <Hubert.Drolet@international.gc.ca>
Subject: Re: ODA Eligibility - P011103 mRNA Manufacturing Hub

Hi Hubert,

Wondering if you got a response on this from [redacted]

Sara

Sent from my iPhone

On Mar 21, 2022, at 8:09 AM, Drolet, Hubert -SWS <hubert.drolet@international.gc.ca> wrote:

[redacted]

Hope you are doing well. It was nice to see you in Paris this week, even if our encounters were brief and interrupted!

We touched based quickly on the sidelines of the wpstat meeting regarding the eligibility of mRna tech transfer, and I was wondering if you had received this query from other donors. Below is some additional detail.

As you can see, this fund is supported by a number members and from my assesment, is of primary benefit to a number of development countries on the DAC list of recipients. Does the Secretariat have a recommendation how to report this fund in the CRS?

Many thanks for your advice and please let me if you would require more information.

GAC - Released under the Access to Informations Act/
AMC - Divulgué en vertu de la loi sur l'accès à l'information

Hubert

Sent from my Bell Samsung device over Canada's largest network.

-------- Original message --------

From: "Schulz, Sara -FMNV" <Sara.Schulz@international.gc.ca>

Date: 2022-03-18 10:58 AM (GMT-05:00)

To: "Drolet, Hubert -SWS" <Hubert.Drolet@international.gc.ca>

Cc: "Guiet, Jeremie -SWS" <jeremie.guiet@international.gc.ca>, "Hisko, Mellissa -FMNV" <mellissa.hisko@international.gc.ca>

Subject: RE: ODA Eligibility - P011103 mRNA Manufacturing Hub

Hi Hubert,

Hope your week of meetings is wrapping up well! Checking in to see if there are any updates on the tech transfer hub following your OECD conversations? Wishing you safe travels home.

Best,

Sara

From: Drolet, Hubert -SWS <Hubert.Drolet@international.gc.ca>
Sent: March 16, 2022 10:28 AM
To: Schulz, Sara -FMNV <Sara.Schulz@international.gc.ca>
Cc: Guiet, Jeremie -SWS <jeremie.guiet@international.gc.ca>
Subject: RE: ODA Eligibility - P011103 mRNA Manufacturing Hub

GAC - Released under the Access to Informations Act/
AMC - Divulgué en vertu de la loi sur l'accès à l'information

This is excellent. You have not missed the boat, so to speak... I will make sure to liaise with DCD staff on the sidelines of this week's mtg and provide you with an update asap. All the best,

Sent from my Bell Samsung device over Canada's largest network.

-------- Original message --------

From: "Schulz, Sara -FMNV" <Sara.Schulz@international.gc.ca>

Date: 2022-03-16 3:24 PM (GMT+01:00)

To: "Drolet, Hubert -SWS" <Hubert.Drolet@international.gc.ca>

Cc: "Guiet, Jeremie -SWS" <jeremie.guiet@international.gc.ca>

Subject: RE: ODA Eligibility - P011103 mRNA Manufacturing Hub

Hi Hubert,

I hope I have not missed the window for you to bring this up while you are with OECD colleagues. We had a number of urgencies earlier this week!

I am sharing with you the confidential proposal from Medicines Patent Pool, the partner that we will be supporting in this WHO-led project. Pasted below are key messages and background from an internal issues brief on the hub. I hope that info is helpful as context. For use in conversation with OECD colleagues, this public Q&A on the Hub and its approach make quite clear that the knowledge, IP and tech transfer will remain in and be owned by the investors (residing in developing countries) but will be made freely available to the recipient facilities (now planning to facilitate technology transfer to 13 LMIC countries). Product development will target the unique needs of LMICs and distribution will also target countries with stated demand for the products in LMICs.

Let me know if anything else would be helpful!

GAC - Released under the Access to Informations Act/
AMC - Divulgué en vertu de la loi sur l'accès à l'information

Best,

Sara

Key messages

- Canada is committed to working collaboratively with others, including governments, multilateral institutions, and industry, to identify and remove bottlenecks, and accelerate the production and equitable distribution of affordable, safe, effective COVID-19 vaccines and other medical countermeasures.

- Canada is working with international partners to address barriers to equitable access of vaccines by improving global capacity to manufacture them.

- At the G20, the Prime Minister announced an investment of up to $15M to support COVAX Manufacturing Taskforce partners and a South African consortium to set up an mRNA technology transfer and manufacturing hub in South Africa.

- This landmark partnership will enable regional development and production of mRNA vaccines and technologies, taking steps now to address one of the major barriers to more equitable access to vaccination beyond the acute phase of the pandemic.

Background

- The COVAX-led mRNA manufacturing hub was announced in June 2021. This initiative will work with COVAX Facility partners (**Medicines Patent Pool and the WHO**) to establish a technology transfer hub for mRNA COVID-19 vaccines in South Africa, facilitate technology transfer to a South African-based recipient manufacturing facility and establish vaccine production by a network of recipient facilities, that currently stretches across 13 countries. The initiative will also explore the broader application of mRNA technologies towards additional infectious diseases in collaboration with the South African Medical Research Council.

[redacted]

GAC - Released under the Access to Informations Act/
AMC - Divulgué en vertu de la loi sur l'accès à l'information

[redacted]

· On February 23, 2022, Hub partners announced new facilities that will receive the mRNA technology in **Bangladesh, Indonesia, Pakistan, Serbia and Vietnam**. This was following an announcement on February 18 2022 announcing facilities joining the network in **Egypt, Kenya, Nigeria, Senegal, South Africa and Tunisia**. This is in addition to three existing recipient facilities: Sinergium (Argentina), Bio-Manguinos (Brazil) and Biovac (South Africa).

· Funders to the Hub include ([redacted]; **France** [redacted] **Canada** $15M (publically announced); **EU** ([redacted], **African Union**, [redacted] **Belgium** ([redacted] **Germany** ([redacted], **Norway** [redacted], (not yet public); and additional donors in final negotiations.

Project Description

· The ultimate aim of this project is the establishment of a Hub network that will enable the transfer of mRNA vaccine technology to develop and produce a COVID-19 mRNA vaccine and related mRNA technologies. This project will support Phases 1 and 2 of the Hub establishment and scale up over 2021-2023.

· Phase 1 will result in Hub facilities prepared, vaccine technology received and/or developed, intellectual property agreements and regulatory processes reviewed, pre-clinical and clinical trials conducted, technology transfer program designed and biomanufacturing training center maintained.

· Phase 2 of the project will result in recipient manufacturing facilities prepared, staff trained in mRNA technology, technology transferred from the Hub, phase 3 trials conducted and vaccines manufactured, with ultimate production at a first facility reaching 200 million doses per year.

· The project will support increased regional availability of mRNA vaccines for COVID-19 and explore the broader application of mRNA technologies towards additional infectious diseases.

· The total budget for this five-year initiative is €92 million.

From: Drolet, Hubert -SWS <Hubert.Drolet@international.gc.ca>
Sent: March 11, 2022 12:24 PM
To: Schulz, Sara -FMNV <Sara.Schulz@international.gc.ca>
Cc: Guiet, Jeremie -SWS <jeremie.guiet@international.gc.ca>
Subject: RE: ODA Eligibility - P011103 mRNA Manufacturing Hub

GAC - Released under the Access to Informations Act/
AMC - Divulgué en vertu de la loi sur l'accès à l'information

Hi Sara,

Great timing. I'll be visiting the OECD next week and can ask the Secretariat (DCD) directly.

I hesitate to give you any answer now as the DCD tends to be quite conservative/restrictive in their interpretation and the devil is in the details.

My instinct is that there is a clear development link wrt technology transfer on both the supply and demand side. My first question to you is whether donors are receiving some sort of benefit out of this collaboration.. or is this cut and dry, I.e. the majority of the benefits accrue to developing countries. Any documentation you can send supporting the latter would help to make certify the ODA eligibility.

Thx!

Sent from my Bell Samsung device over Canada's largest network.

-------- Original message --------

From: "Schulz, Sara -FMNV" <Sara.Schulz@international.gc.ca>

Date: 2022-03-11 9:02 AM (GMT-05:00)

To: "Drolet, Hubert -SWS" <Hubert.Drolet@international.gc.ca>

GAC - Released under the Access to Informations Act/
AMC - Divulgué en vertu de la loi sur l'accès à l'information

Subject: ODA Eligibility - P011103 mRNA Manufacturing Hub

Hi Hubert,

We've not met, I am part of the COVID-19 Taskforce, nice to e-meet you. I am looking to confirm the process to determine a project's ODA eligibility. I was referred to you by Gillian Harris who I believe has worked with you regarding the ODA eligibility of vaccine dose donations. I am the PTL for a new project, Canada has committed $15 million to COVAX partners for a COVID-19 mRNA Technology Transfer Hub, P011103. If you can let me know what documents might be required for SWS to make a determination and what the overall process is, that would be much appreciated!

Best,

Sara

Sara Schulz

Senior Analyst | Analyste Principale

COVID-19 Global Health Response Task Force (fMNV) | Groupe de travail de la réponse sanitaire mondiale de la COVID-19 (fMNV)

Global Health and Nutrition Platforms (MNC) | Plateformes de santé mondiale et de nutrition (MNC)
Health and Nutrition Bureau (MND) | Direction générale de la Santé et de la Nutrition (MND)

sara.schulz@international.gc.ca
Tel. : 343-548-9938

s.21(1)(b)

GAC - Released under the Access to Informations Act/
AMC - Divulgué en vertu de la loi sur l'accès à l'information

Schulz, Sara -MNV [She,Her | Elle]

From: Hisko, Mellissa -FMNV
Sent: May 4, 2022 11:50 AM
To: Murphy, Liam -MFM; *oMFM
Cc: Cain, Megan -FMNV; Tabah, Joshua -MND; Schulz, Sara -FMNV; Bender, Tracey -FMNV
Subject: FOR APPOVAL: Manufacturing Questions from oMINE

Hi Liam,

We have been asked for information on manufacturing programming options by oMINE. Below are the questions and our proposed responses for MFM approval prior to sending.

Thank you.

Mellissa

****

Increasing funding to the South Africa mRNA hub and PAHO initiative (assuming ACT-A eligibility). Does the team have advice/options ?

- Increasing manufacturing capacity in low and middle incoming countries (LMICs) is a pillar of the proposed signature COVID-19 Vaccination Action Canada (C-VAC initiative). Opportunities to support vaccine manufacturing have been identified in the C-VAC programming options proposed for Budget 2022 allocations currently with oMINE.

- As part of the C-VAC elements of the Budget 2022 programming options, Global Affairs Canada is proposing to allocate $30 million to vaccine manufacturing to enhance ACT-Accelerator partner efforts in support of regional and national manufacturing initiatives where C-VAC priority countries have committed to scale-up vaccine production capacity. All programming must contribute to meeting Canada's ACT-Accelerator burden share consistent with Budget 2022.
- As part of the $30 million commitment, GAC proposes to allocate $15 million to the mRNA Vaccine Technology Transfer Hub Programme in partnership with Medicines Patent Pool (MPP) and WHO. To be fully funded, the initiative requires an additional 27 million CAD out of a budget of 117 million CAD for 2021-24. Canada's $15M contribution would support stage III clinical trials, as well as fund grants to recipient manufacturing facilities in [redacted] C-VAC countries in the African region (e.g. Nigeria) to ensure successful scale-up of training, equipment and operations. This would bring the total Canadian contribution to the Hub to $30 million, all of which is ACT-Accelerator gap reducing, making Canada a top donor to this initiative. The remaining funding gap is expected to be covered by new donors to the project [redacted]
- GAC is also proposing to allocate up to $15 million in funding to PAHO to support PAHO's regional manufacturing platform and provide targeted assistance to scale-up vaccine production capacity in the Latin American region, including in C-VAC priority countries - pending final validation from the ACT-Accelerator Secretariat that funding to PAHO for manufacturing would be considered gap reducing and count towards the achievement of Canada's burden share.

Other possibilities for funding global south manufacturing capacity, such as CEPI/IPD MADIBA project in Senegal?

GAC - Released under the Access to Informations Act/
AMC - Divulgué en vertu de la loi sur l'accès à l'information

- There are other opportunities to contribute to global south manufacturing efforts bilaterally, however, these are not being undertaken by ACT-A partners and would not count as gap reducing to the ACT-Accelerator. At this time, the Department does not have any internal resources to support initiatives outside of ACT-A.
- GAC has assessed possible contributions to CEPI, including for earmarked manufacturing efforts in priority countries, however, funds specifically for manufacturing through CEPI do not count as gap reducing to the ACT-Accelerator.

From: Assefa, Abebech -MINE <Abebech.Assefa@international.gc.ca>
Sent: May 2, 2022 4:15 PM
To: Cain, Megan -FMNV <Megan.Cain@international.gc.ca>; Tabah, Joshua -MND <Joshua.Tabah@international.gc.ca>; Hisko, Mellissa -FMNV <mellissa.hisko@international.gc.ca>
Cc: *MINE-Dept Unit/Unité ministérielle <D-MINE-DeptUnit@international.gc.ca>; *DME Advisors <D-DMEAdvisors@international.gc.ca>
Subject: ACT-A Breakdown

Good afternoon,
oMINE would like to receive advice from the team on the following:

(1) increasing funding to the South Africa mRNA hub and PAHO initiative (assuming ACT-A eligibility). Does the team have advice/options ?
(2) other possibilities for funding global south manufacturing capacity, such as CEPI/IPD MADIBA project in Senegal

Thank you

Abebech

s.19(1)

s.21(1)(b)

GAC - Released under the Access to Informations Act/
AMC - Divulgué en vertu de la loi sur l'accès à l'information

Tarr, Michael -MNV [He,Him | Il]

From: Charles Gore <[redacted]
Sent: May 30, 2022 11:37 AM
To: Tarr, Michael -FMNV; Gelise McCullough
Cc: Schulz, Sara -FMNV
Subject: Re: Follow up

Dear Mike,

We're very happy that you're happy! Largely the work of Gelise, [redacted]

That's great news about the funding. I think we can have something to you by the end of the week, assuming I can get time with WHO to agree it. As you point out, it will need to be fairly high level at this point

Very best

Charles

Charles Gore | Executive Director | Medicines Patent Pool
Rue de Varembé 7, 1202 Geneva, Switzerland
Tel: +41 22 533 5050 | Direct Tel : [redacted]
Mob: [redacted]
E-mail: [redacted]
@CharlieGore
Website | Newsletter

From: "Michael.Tarr@international.gc.ca" <Michael.Tarr@international.gc.ca>
Date: Monday, 30 May 2022 at 14:24
To: Charles Gore <[redacted] Gelise McCullough [redacted]
Cc: "Sara.Schulz@international.gc.ca" <Sara.Schulz@international.gc.ca>
Subject: Follow up

Dear Charles and Gelise,

Hope you're both doing well and recovered from the flurry of event activity and busy WHA week. Thank you for being nimble with Canada's last-moment change in representative. I was able to watch the full recording and thought the questions did a great job of capturing the various program facets and partner perspectives. The video is also fantastic and a great asset to share here.

Although we unfortunately weren't able to have the Minister deliver a video announcement, as Sara previously mentioned Canada's next ACT-A allocations are taking shape, including further support to the mRNA Hub program.

In this light, wondering if you would be able to provide a very brief outline – 1-2 pages max – of how an investment at [redacted] could be put to use.

A few notes:

- The funding would likely be available in January 2023 (for an implementation period of up to 24 months).

s.21(1)(b)

GAC - Released under the Access to Informations Act/
AMC - Divulgué en vertu de la loi sur l'accès à l'information

- Canada would like to continue core funding to the Hub in Cape Town while also supporting expansion in the Spokes. The countries we are particularly interested in are Senegal, Nigeria, Kenya, and Bangladesh.
- [redacted]
- Appreciate that Spoke budgets and workplans may still to be defined – at this juncture we would only require high-level areas of activity. We can fill in the details down the road.

Please let us know if any further info would be helpful.

Would be very grateful to receive the outline by end of this week if possible.

Best regards,
Mike

Mike Tarr
Senior Analyst | Analyste Principal
COVID-19 Global Health Response Task Force (fMNV) | Groupe de travail de la réponse sanitaire mondiale de la COVID-19 (fMNV)
Health and Nutrition Bureau (MND) | Direction générale de la Santé et de la Nutrition (MND)
Global Affairs Canada | Affaires mondiales Canada
michael.tarr@international.gc.ca

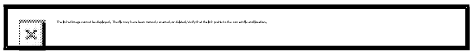

GAC - Released under the Access to Informations Act/
AMC - Divulgué en vertu de la loi sur l'accès à l'information

s.15(1) - International

s.21(1)(b)

medicines
patent
pool

Geneva

Object: request for funding for the mRNA Technology Transfer Hub Programme

With this letter, the mRNA Technology Transfer Hub Programme is requesting [redacted] to support the set up and scale-up of the South African mRNA Vaccine Technology Transfer Hub in an amount of [redacted] and provide technical and financial support to the technology recipients (spokes) in an amount of [redacted] This financial support will be extremely important to contribute to improving health and health security in LMICs through sustainable, regional production of vaccines.

<u>Programme activities:</u>

To achieve the Programme objective of establishing or enhancing sustainable mRNA vaccine manufacturing in regions with no or limited capacity, WHO and MPP have envisaged a hub/spoke model where the hub is developing the technology that is then is transferred to other manufacturers in LMICs. In this regard, WHO and MPP have:

1) initiated a collaboration with the South African mRNA Vaccine Consortium (SAMVAC) to:
   a) establish a technology transfer hub (technology donor) for mRNA COVID-19 vaccines at Afrigen in South Africa;
   b) transfer the technology developed by the hub to the first spoke (technology recipient), Biovac;
   c) develop a pipeline of novel mRNA vaccine candidates for SARS-CoV2 and other diseases through a collaboration of SAMRC with other research institutions.

2) identified 14 additional recipient manufacturing facilities (spokes) all around the world that have expressed their interest in the Programme and will receive the technology transfer and will then establish vaccine production.

<u>Programme funding:</u>

Please find below an outline on the general Programme needs and on how a contribution of [redacted] could be put in use to support activities conducted in the framework of the mRNA Technology Transfer Hub Programme.

To implement the roadmap linked to the South African mRNA hub (**points 1a-c above**), the South African Consortium and supporting infrastructure will require c. €103 million funding over a 5-year period (Q4/2021-Q4/2026):

- c. €8 million for the secretariat to coordinate and support the overall effort.
- c. €41 million for Afrigen for the establishment of the hub, including the preparation of facilities, the development of vaccine technology, the reception of the necessary regulatory approvals, the conduct of pre-clinical studies, the design of the tech transfer program, and the maintenance of the training centre.
- c. €15 million for Biovac for the establishment of the first spoke, including the establishment of a plan for equipment and processes for Drug Substances and Drug Products, the preparation of facilities, the training of staff and receipt of the

s.21(1)(b)

GAC - Released under the Access to Informations Act/
AMC - Divulgué en vertu de la loi sur l'accès à l'information

technology from Afrigen, the process scale-up, the demonstration and processing of validation runs.

- c. €24 million for SAMRC to develop local innovation and products, including the establishment of a pipeline of vaccine candidates (TB, HIV etc.) with pre-clinical testing, and the optimization of technology for LMIC application.
- c. €15 million for the conduct of clinical trial activities.

Currently the received and committed funding is covering about 60% of the needs for this component of the Programme activities, although there are ongoing discussions with potential donors. For this reason, Canada's funding of CAD 7.5 million is critical to fill the currently still existing financial gap and ensure the smooth implementation of the above mentioned activities.

Exact allocation of the budget to activities is still not possible while awaiting the allocation of a large national contribution that has very restrictive use. Canada's flexibility around exact allocation on the first CAD 15 million has been extremely helpful and we would request that this can applied to this second tranche of funding.

In addition, the Programme is intending to facilitate technology transfer to recipient manufacturing facilities and support the establishment of vaccine production at the recipient facilities (**point 2 above**) by providing technical and financial support. In the original call for Expressions of Interest spokes were told they would need to be self-supporting and therefore no budget was allocated to their support. However, since the selection of the spokes it has become apparent that some of them will need both some financial and extra technical support. The budget required for these activities and the exact workplans are still under definition as we assess their needs.

We currently expect to need to fund the following for some of them:

- travel and consultancy support for on-site visits to assess the needs of those spokes clearly not currently in a position to receive a technology transfer;
- training related expenses including travel (the spokes will all undergo an introductory training in the mRNA technology and some of them will be offered a range of training, including in biomanufacturing at the training hub in South Korea);
- extra technical support through consultants to support readiness for tech transfer, including advice on plant and equipment;
- consultancy support on models of sustainability to ensure their ability to respond to the next pandemic;
- financial and technical support to facilitate the technology transfer to the receiving facilities and ensure successful implementation of the technology.

The Programme is in the process of better detailing and costing the above mentioned activities and determining which spokes really need support. We note Canada's interest in Senegal, Kenya, Nigerian and Bangladesh and will keep this in mind during selection. We have already started approaching some possible funding sources so Canada's funding of [redacted] could therefore be the first received for this specific purpose and would be extremely helpful in leading the way and prompting other donors to step up.

GAC - Released under the Access to Informations Act/
AMC - Divulgué en vertu de la loi sur l'accès à l'information

medicines
patent
pool

The mRNA Technology Transfer Hub Programme and gender equality

Activities included under the realm of the Programme are implemented in accordance with the principles of good governance, human rights, gender equality, environmental sustainability and protection and the inclusion of socially or economically deprived groups.

Gender-responsive components are included into the planning and implementation of this project, with a specific focus on strengthening gender equality in manufacturing and in access to vaccines.

More in detail, the Programme is primarily taking into account the following:

- Contribute to an inclusive manufacturing sector by ensuring adequate gender responsiveness of selected hub and spokes. Gender equality is specifically considered as a criterion in the selection of new hubs and spokes.
  - Update: A question on gender equality policy was part of the Expression of Interest questionnaire that was completed as part of the selection of 15 spoke facilities (Your organization's core policies: Does your organization have core policies with respect to gender equality, conflict management and/or health security?). The response to this question was taken into account for each of the spoke's selection.

- Actively promote the inclusion of women in the biomanufacturing training offered to the hub and spokes and in the virtual/hands-on trainings performed in the context of the technology transfer.
  - Update: The project has now the adopted the objective of at least 40% women participation in trainings. So far, 5 spoke facilities have received an introductory training on the mRNA technology at Afrigen. Out of the 27 participants, 11 were women (41%).

- Clinical trials developed and conducted with gender inclusion embedded.
  - Update: Clinical trial participants will not be selected based on gender, to avoid introduction of a bias (women and men will be both offered trial participation and be included in the study if the meet inclusion criteria and do not meet exclusion criteria). Data analysis will also be conducted by gender.

- Gender-balanced recruitment of new trainees and staff at all levels (hub).
  - Update: The hub (Afrigen), primary recipient of the Canada's funding, is closely monitoring the gender of people hired to work for the project so that gender-balance is maintained. A workforce report with gender analysis was provided by Afrigen in July.

- Training modules on Protection for Sexual Exploitation and Abuse (PSEA) and workplace safety promotion and/or developed and delivered.
  - Update: This training module will likely no longer be offered to spokes and hub.

- Hub and recipient facilities gender policies strengthened

GAC - Released under the Access to Informations Act/
AMC - Divulgué en vertu de la loi sur l'accès à l'information

s.15(1) - International

s.21(1)(b)

medicines
patent
pool

- Update: Gender specific policies at the hub (Afrigen) and spokes (Biovac and others) receiving the funding will be reviewed and might be requested to be strengthened as much as possible, if needed.

Draft budget

**1- Continued support of technology transfer activities South African Hub (Afrigen) and spoke (Biotec)**	
• GMP (R&D) COVID-19 mRNA vaccine manufacturing process and analytical methods development<br>• mRNA vaccine batch production and testing for a Phase I clinical trial	
• GMP COVID-19 mRNA vaccine manufacturing process scale-up and process and analytical methods validation<br>• mRNA vaccine batches production and testing for a Phase III clinical trial	
• Develop a second-generation mRNA vaccine technology and establish a pipeline of vaccine candidates for Low-Middle-Income Countries	
**2- Additional support for technology transfer activities across the spoke network**	
• Support to local regulatory authorities to achieve Maturity level 3 for vaccines	
• Training of local workforce in GMP manufacturing, vaccinology, mRNA technology	
• Technology transfer support to spokes (including technical support before and during technology transfer, equipment and consultancy support on models of sustainability)	
**Total Direct Costs**	
**Indirect Cost (overhead)**	
**Total estimated budget**	

GAC - Released under the Access to Informations Act/
AMC - Divulgué en vertu de la loi sur l'accès à l'information

Schulz, Sara -MNV [She,Her | Elle]

From: Hisko, Mellissa -FMNV
Sent: June 9, 2022 8:47 AM
To: Schulz, Sara -FMNV
Subject: RE: FOR REVIEW: CVAC Country Action Plans

Thanks Sara will check these out and move to Megan ASAP. I will recommend we send to geos directly for feedback and integration. We need to give them one week.

Heading to an appointment now back by 10ush

From: Schulz, Sara -FMNV <Sara.Schulz@international.gc.ca>
Sent: June 9, 2022 8:43 AM
To: Hisko, Mellissa -FMNV <mellissa.hisko@international.gc.ca>
Subject: FOR REVIEW: CVAC Country Action Plans

Hi Mellissa,
Thanks for this feedback. Based on these comments, I have brought all 13 country action plans up to date with programming summaries. They are ready for final review before going to the geos. They are all in the attached folder.

One point for your decision – [redacted]

Best,
Sara

From: Hisko, Mellissa -FMNV <mellissa.hisko@international.gc.ca>
Sent: June 8, 2022 8:12 AM
To: Schulz, Sara -FMNV <Sara.Schulz@international.gc.ca>
Subject: RE: CVAC Country Action Plans

Thanks for these Sara – on the right rack.

For Nigeria: would like to see us use more plain language explanation and refer less to UNICEF programming names and programs…. Describe what will be done not the UNICEF mechanism through which it will flow. Makes it hard to follow and MINE wont know what these things are.

Haiti: Great, minor comment.

From: Schulz, Sara -FMNV <Sara.Schulz@international.gc.ca>
Sent: June 6, 2022 1:57 PM
To: Hisko, Mellissa -FMNV <mellissa.hisko@international.gc.ca>
Subject: CVAC Country Action Plans

Hey Mellissa,

GAC - Released under the Access to Informations Act/
AMC - Divulgué en vertu de la loi sur l'accès à l'information

For your review, here are the filled in action plans for Nigeria and Haiti. Let me know if this is on the right track and I will adjust all the others to match. Tell me if we need to go more visual ;).

Sara

GAC - Released under the Access to Informations Act/
AMC - Divulgué en vertu de la loi sur l'accès à l'information

Schulz, Sara -MNV [She,Her | Elle]

From: Morris, Phedra Moon -DHAKA -DA
Sent: June 29, 2022 10:57 PM
To: Schulz, Sara -FMNV
Cc: Sebhatu, Joseph -DHAKA -DA; Nickerson, Elaine -OAK; Yampolsky, Raya -DHAKA -GR; Hisko, Mellissa -FMNV; Andolfatto, Maegen -DHAKA -DA
Subject: Re: URGENT INPUT requested - Canada's Global Initiative for Vaccine Equity (CAN-GIVE)

Sounds good! That is quite an interesting component of the initiative - hoping it pans out.

Cheers
Phedra Moon

Sent from my iPhone

On 30 Jun, 2022, at 02:30, Schulz, Sara -FMNV <Sara.Schulz@international.gc.ca> wrote:

Thanks very much to you and the team for this feedback, Phedra. Noted on the programming content regarding humanitarian populations including and beyond the Rohingya + host populations. Regarding Incepta Ltd., the conversations with Medicines Patent Pool are still early stages, please hold off on engaging locally for now. MPP are expecting to receive more detailed plans from the company shortly that will inform the sustainability and partnership approach.

Best,
Sara

From: Morris, Phedra Moon -DHAKA -DA <PhedraMoon.Morris@international.gc.ca>
Sent: June 27, 2022 9:54 AM
To: Schulz, Sara -FMNV <Sara.Schulz@international.gc.ca>
Cc: Sebhatu, Joseph -DHAKA -DA <Joseph.Sebhatu@international.gc.ca>; Nickerson, Elaine -OAK <ELAINE.NICKERSON@INTERNATIONAL.gc.ca>; Yampolsky, Raya -DHAKA -GR <Raya.Yampolsky@international.gc.ca>; Hisko, Mellissa -FMNV <mellissa.hisko@international.gc.ca>
Subject: FW: URGENT INPUT requested - Canada's Global Initiative for Vaccine Equity (CAN-GIVE)

Hello Sara,

Please see below what the team has put together regarding the CAN-GIVE initiative. In summary – the feedback is that this would make sense for Bangladesh and support important gap-filling for a large population which has a substantial number of vulnerable people. Having said that, as the vast majority of the vaccination program in Bangladesh has been national – including for the Rohingya – [redacted]

Cheers,
Phedra Moon

GAC - Released under the Access to Informations Act/
AMC - Divulgué en vertu de la loi sur l'accès à l'information

In response to our questions on if the GoB would have any reservations with regards to having Bangladeshis and Rohingya refugees as the beneficiary population of the proposed UNICEF CAN-GIVE project, [redacted] has confirmed both in writing and during a phone that the GoB would be supportive of the CAN-GIVE initiative.

Responses by [redacted] UNICEF Bangladesh:

- What are you views/comments on this proposed initiative? UNICEF applauds the initiative of Canada for CAN-GIVE and supporting the vaccination efforts against COVID and support the essential health services. There is an ongoing need to support the *essential health services*, as more children and women are affected by lower capacity in the health sector to provide essential health services due to the COVID response [[i.e. due to resources being allocated to COVID response]].
- What are your views/comments on the proposed beneficiary population. We are aware that there can be sensitivities with regards to the refugee population. UNICEF supports both, the Rohingya refugees and the Bangladeshi population. The Ministry has been requesting UNICEF's support for the vaccination of the Rohingya, both in Cox Bazar and Bashan Char. In Cox Bazar, 488,552 Rohingya received first dose (12 years and above), 358,486 received 2 doses (12 years and above). The child vaccination campaign started and achieved good coverage. In Bashan Char the vaccination also is ongoing. We also support PHC in the camps and we support outbreak response (AWD/Cholera/COVID/Diphtheria).
  At the same time, UNICEF needs to support the Bangladesh MoHFW in the COVID response through support in national planning, coordination, support to the cold chain, vaccine management, communications to sustain demand and support he vaccination efforts. Equally, UNICEF needs to support the essential health services to ensure Infection Prevention Control and quality MNCAH services. UNICEF also supported the oxygen infrastructure in the health system with an oxygen landscape analysis, installation of piped oxygen in 30 hospitals, manifold oxygen systems in 60 upazillas, provision of oxygen concentrators and accessories. UNICEF now use this increased capacity to improve the quality of care using oxygen among newborns and children.
- [redacted]
- Anything that stands out? There are still immediate needs for the system strengthening of oxygen and capacity building on safety and maintenance of the systems, support to essential health services, and data for decision making. For COVID vaccination, the most urgent needs are covered in 2022. UNICEF needs financial support with grants valid into 2023 for support to ongoing vaccination, system strengthening and support to essential health services.

<u>Task 1: on content of proposal</u>

From the health team:

- UNICEF is an active partner for supporting implementation of "Bangladesh preparedness and response plan" (BPRP) for the coronavirus disease 2019 (COVID-19) 2020 (copy attached);
- UNICEF has been a long time partner with Canada to support Immunization and cold chain installation nationally and at district level in Bangladesh.

Best copy available / Meilleure copie disponible

GAC - Released under the Access to Informations Act/
AMC - Divulgué en vertu de la loi sur l'accès à l'information

s.19(1)

s.21(1)(b)

s.15(1) - International

- I understand [redacted] that Currently UNICEF is supporting GoB in upgrading cold chain installation, maintenance, transportation, provision of oxygen etc.
- We also believe that this is a good initiative to support humanitarian and at-risk population, especially for the Rohingya. I suggest to add few of the climate vulnerable locations and people apart from Rohingya and its host neighborhood population for vaccine equity and primary health care (e.g., hard to reach areas of Sunamganj, Kishorganj and similar riverine areas.)
- The GoB has prepared "Bangladesh Preparedness and Response Plan for COVID-19" (copy attached) where they have highlighted the following:
    - Given the current understanding of transmission dynamics of COVID-19, densely populated areas such as the camps and other concentrated settlements in the District present a particular challenge, since an approach based on physical distancing and the typical containment strategies used in high income settings cannot be implemented in the same way. Different work and housing patterns preclude this level of isolation. Tailored approaches are being applied instead.

- **Activities:**

    - Multi-sector actions supporting both the public health and indirect impacts of the pandemic include:

        1. Reducing the footprint of the operation by delivering critical services only;
        2. Mitigating the risk of interruptions to the existing life-saving response, by ensuring preventive measures are taken during all critical service delivery and urging physical distancing to the extent possible in the camp setting;
        3. Communicating key messages through all Sector operations;
        4. In support of the District health response, providing multi-sector support to the existing and planned isolation, treatment, and quarantine facilities;
        5. Supporting families that will be providing home-based care for COVID-19 patients;
        6. Protecting older persons;
        7. Adjusting cyclone and monsoon preparedness plans to account for COVID-19;
        8. Augmenting Government social safety nets for the poorest and most vulnerable Bangladeshis in the District whose livelihoods will be impacted by the pandemic.

- We support the [redacted] initiative to Medicines Patel Pool / COVAX mRNA Vaccine Technology Transfer Hub efforts to facilitate training, technical support and technology to transfer to a local firm "***Incepta Vaccine Ltd." In Bangladesh.*** Incepta Vaccine Ltd. is a profit-making private company. How Incepta will work for vaccine equity after getting the technical assistance and technology for vaccine manufacturing can be discussed briefly. The approval from Directorate General of Drug Administration (DGDA) and other regulatory mechanisms to be ensured in due process.

Task 2: Engagement Chart

Canadian Mission and Senior Official Engagement	
• **DHAKA-DA** ***to participate in meetings*** as scheduled to engage on financing, prioritization and operational vaccine delivery efforts being facilitated by UN agencies, country government and donors.	• These discussions are held as the Health Development Part Consultative Group' which m monthly. CAN is represented health team lead

GAC - Released under the Access to Informations Act/
AMC - Divulgué en vertu de la loi sur l'accès à l'information

Best copy available / Meilleure copie disponible

• **Head of Mission/Head of Cooperation** bilateral meeting with UNICEF and WHO country Reps as focal points, and if possible Secretary for the Ministry of Health to discuss COVID-19 response and integration within primary health care/routine immunization, especially for humanitarian populations.	• Please note that Bangladesh has [illegible] vast majority of their vaccine pro[illegible] distribution, etc. They had a few [illegible] bilateral donations of large quant[illegible] 10 million doses), but very much [illegible] their success (rightly) to be natio[illegible] repeated requests of interest for [illegible] the past year we have received a [illegible] response. It does not mean that t[illegible] not gaps and we do think this sup[illegible] be well utilised, but it will not be [illegible] important contribution by Bangla[illegible] their overall large scale vaccinati[illegible] program. We should not expect c[illegible] engagement on this specific proje[illegible] Canada. We did not respond to th[illegible] requests for vaccines for refugee[illegible] we had vaccines available for dor[illegible] past year. There will need to be s[illegible] of this.
• Continued Canadian participation at a technical level in the Health Population Nutrition DP Consortium and Bangladesh Preparedness and Response Plan working group	• BPRP working groups meetings h[illegible] regularly during early C-19 pande[illegible] 2021), and since then is need bas[illegible] Canada will patriate as and when [illegible] meeting organized.
• Potential site visit could be included – if at the same time as a vaccination booster campaign – during high-level visit. Do not propose visits specifically for this activity as scheduling is last minute, and again, apart from large-scale bilateral country to country donations, support via multilaterals is seen as a given more than something to make fanfare around.	

Begin forwarded message:

From: "Schulz, Sara -FMNV" <Sara.Schulz@international.gc.ca>
Date: 23 June, 2022 01:00:07 GMT+6
To: "Morris, Phedra Moon -DHAKA -DA" <PhedraMoon.Morris@international.gc.ca>
Cc: "Hisko, Mellissa -FMNV" <mellissa.hisko@international.gc.ca>, "Nickerson, Elaine -OAK" <ELAINE.NICKERSON@international.gc.ca>, "Yampolsky, Raya -DHAKA -GR" <Raya.Yampolsky@international.gc.ca>

GAC - Released under the Access to Informations Act/
AMC - Divulgué en vertu de la loi sur l'accès à l'information

Subject: RE: URGENT INPUT requested - Canada's Global Initiative for Vaccine Equity (CAN-GIVE)

Thanks very much for this quick response Phedra. Noting the work week, we have been able to get some flex and welcome feedback on the summary by **next Wednesday June 29th**.

The UNICEF concept was developed by UNICEF global, with consultations via their national offices and COVAX partner, WHO. The initiative is part of Canada's multilateral funding to the ACT-Accelerator, some of which are pooled flexible funds, some of which are this CAN-GIVE country-earmarked approach at MINE's request. As context, the request for a humanitarian focus in a few targeted countries also came from MINE, though you'll see in the program description UNICEF has struck a balance between humanitarian response and broader health system support.

The details in the current UNICEF proposal for each of the 13 countries are quite high level - essentially what you see in the action plan document I shared - and we have some time over the next months to refine the concepts further based on field-level feedback and confirmation.

Please don't hesitate to reach out for additional context or info. Sharing the Minister's high-level announcement of CAN-GIVE for references as well. Elaine, let me know what is most helpful!

Best,
Sara

From: Morris, Phedra Moon -DHAKA -DA <PhedraMoon.Morris@international.gc.ca>
Sent: June 22, 2022 9:47 AM
To: Schulz, Sara -FMNV <Sara.Schulz@international.gc.ca>
Cc: Hisko, Mellissa -FMNV <mellissa.hisko@international.gc.ca>; Nickerson, Elaine -OAK <ELAINE.NICKERSON@INTERNATIONAL.gc.ca>; Yampolsky, Raya -DHAKA -GR <Raya.Yampolsky@international.gc.ca>
Subject: RE: URGENT INPUT requested - Canada's Global Initiative for Vaccine Equity (CAN-GIVE)

Hello Sara,

This is the first we have heard of this. Who has developed the concepts? Interesting. Do we know GoB's involvement? Could we see the concept notes that were done and who from Bangladesh was involved? The Rohingya component would be WHO led and so hopefully UNICEF outlined the partnership.

Just to flag that DHAKA is a Sun-Thurs workweek.

Very interesting idea on the vaccine production.

GAC - Released under the Access to Informations Act/
AMC - Divulgué en vertu de la loi sur l'accès à l'information

Cheers,

Phedra Moon

From: Schulz, Sara -FMNV <Sara.Schulz@international.gc.ca>
Sent: June 22, 2022 4:43 PM
To: Nickerson, Elaine -OAK
<ELAINE.NICKERSON@INTERNATIONAL.gc.ca>
Cc: Hisko, Mellissa -FMNV <mellissa.hisko@international.gc.ca>; Morris, Phedra Moon -DHAKA -DA <PhedraMoon.Morris@international.gc.ca>
Subject: URGENT INPUT requested - Canada's Global Initiative for Vaccine Equity (CAN-GIVE)
Importance: High

Hi Elaine,

In follow up to earlier emails (below), MND has now received programming concepts from ACT-A partners for the signature vaccine delivery initiative we have all been working on the past few months. MINE is aiming to publically announce the initiative later this morning. Bangladesh is featured as one of 13 countries in the initiative, newly named Canada's Global Initiative for Vaccine Equity (CAN-GIVE) – used to be called CVAC but we have found a catchier name!

Priority programming needs in Bangladesh were assembled based on the country profiles that HQ and mission staff worked on earlier this year. While originally the concept had been to support some bilateral programming, you may recall that funding for this initiative has to be programmed through ACT-Accelerator partners to be consistent with Budget 2022 - in this case, UNICEF and Medicines Patent Pool. Following engagement with these partners, we now have a notional country budget, programming scope, and some advocacy/engagement activities in country. We are being asked (urgently) by oMINE to share by Monday next week a detailed plan across the CAN-GIVE countries.

Would it be possible to urgently give this short two page action plan a once over and adjust anything you and mission staff deem necessary, particularly the advocacy and engagement portion, by Friday COB? Don't hesitate if a quick call would be the more expedient way to finalize the inputs, I'm available if you'd like to speak. In particular, your insight on the suggested advocacy engagement options would be most helpful – these built on partnerships that were flagged by mission staff in the initial country needs assessment phase earlier so hopefully are well aligned from your perspective, but please validate and adapt as required. [redacted]

With thanks,

GAC - Released under the Access to Informations Act/
AMC - Divulgué en vertu de la loi sur l'accès à l'information

Sara

Sara Schulz
Senior Analyst | Analyste Principale
COVID-19 Global Health Response Task Force (fMNV) | Groupe de travail de la réponse sanitaire mondiale de la COVID-19 (fMNV)
Global Health and Nutrition Platforms (MNC) | Plateformes de santé mondiale et de nutrition (MNC)
Health and Nutrition Bureau (MND) | Direction générale de la Santé et de la Nutrition (MND)
sara.schulz@international.gc.ca
Tel. : 343-548-9938

From: Tabah, Joshua -MND <Joshua.Tabah@international.gc.ca>
Sent: May 25, 2022 3:58 PM
To: Cesaratto, Sylvia -NDD <Sylvia.Cesaratto@international.gc.ca>; Cohen, Sara -NLD <Sara.Cohen@international.gc.ca>; Dutton, Jess -ESD <Jess.Dutton@international.gc.ca>; Epp, Weldon -OPD <Weldon.Epp@international.gc.ca>; Flanagan Whalen, Ann -ELD <Ann.FlanaganWhalen@international.gc.ca>; Fry, Robert -EUD <Robert.Fry@international.gc.ca>; Guttman, Tamara -WED <Tamara.Guttman@international.gc.ca>; Hartman, David -OAD <David.Hartman@international.gc.ca>; Khan, Tarik -WFD <Tarik.Khan@international.gc.ca>; Kutz, Heidi -ECD <Heidi.Kutz@international.gc.ca>; Lebleu, Marcel -WWD <Marcel.Lebleu@international.gc.ca>; Lundy, Peter -OSD <Peter.Lundy@international.gc.ca>; Thissen, Sonya -NMD <Sonya.Thissen@international.gc.ca>; Walsh, Eric - NGD <Eric.Walsh@international.gc.ca>; Parenteau, Jean-Bernard -KSD <jean-bernard.parenteau@international.gc.ca>; Smith, Andrew (Drew) -PVD <Andrew.Smith2@international.gc.ca>; Wega, Edmond -DPD <Edmond.Wega@international.gc.ca>
Cc: Mollica, Karen -NDS <Karen.Mollica@international.gc.ca>; Sorger, Carmen -NDC <Carmen.Sorger@international.gc.ca>; Labelle, Chantal -NLA <Chantal.Labelle@international.gc.ca>; McLaughlin, Emily -NLG <Emily.McLaughlin@international.gc.ca>; Boyd, Sean -ESC <sean.boyd@international.gc.ca>; De Sorgher, Alizee -OPD <Alizee.DeSorgher@international.gc.ca>; Martin, Elisha Jean -ELD <ElishaJean.Martin@international.gc.ca>; Saint-Arnaud, Louis -EUD <Louis.Saint-Arnaud@international.gc.ca>; Clark, Andrew -WEK <Andrew.Clark@international.gc.ca>; Frost, Gillian -OAK <Gillian.Frost@international.gc.ca>; Simard, Nicolas -WFO <Nicolas.Simard@international.gc.ca>; Roma, Giovanna -BRLIN -GR <Giovanna.Roma@international.gc.ca>; Harper, Diane -WWC <Diane.Harper@international.gc.ca>; Gawn, Mark -OSF <Mark.Gawn@international.gc.ca>; Belzile, Anne-Sophie -NMD <Anne-Sophie.Belzile@international.gc.ca>; Levasseur-Rivard, Sacha -NGI <Sacha.Levasseur-Rivard@international.gc.ca>; Brown, Geneviève -KSN <Genevieve.Brown@international.gc.ca>; Watkinson, Meghan -PVP

GAC - Released under the Access to Informations Act/
AMC - Divulgué en vertu de la loi sur l'accès à l'information

<meghan.watkinson@international.gc.ca>; McKenzie, Renée -DPD <Renee.McKenzie@international.gc.ca>; Cain, Megan -FMNV <Megan.Cain@international.gc.ca>; Hisko, Mellissa -FMNV <mellissa.hisko@international.gc.ca>; Dunkley Elliott, Emma -FMNV <Emma.DunkleyElliott@international.gc.ca>; Chenier, Kristen -MNC <Kristen.Chenier@international.gc.ca>; Paradis, Mylene -MNG <Mylene.Paradis@international.gc.ca>
Subject: COVID response: C-VAC update

(Le français suit)

Colleagues,

I am writing to share an update regarding the COVID-19 Vaccination-Action Canada (C-VAC) signature initiative, following the announcement by the Prime Minister at the Global COVID-19 "Biden" Summit on May 12, 2022 of the further $732M for ACT-A.

Following engagements with DME and MINE, we have now received the greenlight on the proposed programmatic partners for the C-VAC initiative, including notional program areas of focus and budgets - UNICEF, WHO, PAHO, and Medicines Patent Pool. While implementing through only multilateral partners is different from the initial C-VAC concept, the core countries of work will not change, nor does the opportunity for programming impact. The initiative continues to present a real opportunity to contribute to concrete progress on COVID-19 vaccination coverage in your countries of responsibility. The attached document provides a snapshot of programming options that will be pursued.

As a next step, Megan Cain and the FMNV team are engaging the ACT-A partners to elaborate programming options. Each country will have its own programming plan, as originally conceived, with earmarked funding through relevant partners. The FMNV team will propose programmatic elements based on the country analysis your teams provided and input from the implementing multilateral partner. These light-touch **country programming plans will be sent to your teams for validation and comment by May 30, 2022.** While programming plans are being elaborated, we ask that you begin to identify country-level opportunities for Canadian political leadership - including MINE participation in relevant national, regional or global fora (e.g.: field visits and upcoming travel), as well as key opportunities for Senior mission staff to engage countries, donors, and partners on Canada's vaccine roll-out efforts through this initiative.

As always, should you have any questions, please do not hesitate to reach out to Task Force Director Megan Cain, or DD Mellissa Hisko.

Thank you for your ongoing cooperation in this evolving initiative.

Joshua

GAC - Released under the Access to Informations Act/
AMC - Divulgué en vertu de la loi sur l'accès à l'information

****
Collègues,

Je vous écris pour partager une mise à jour pour l'initiative phare COVID-19 Vaccination-Action Canada (C-VAC) suite à l'annonce du $732M pour ACT-A faite par le Premier ministre lors du Sommet mondial sur la COVID-19, le 12 mai 2022.

Suite aux engagements avec le Bureau du Sous-ministre du Développement international (DME) et le Ministre de la Coopération internationale (MINE), nous avons maintenant reçu le feu vert pour les partenaires programmatiques proposés pour C-VAC, y compris les niveaux de référence et les budgets théoriques - UNICEF, OMS, PAHO et Medicines Patent Pool. Bien que la mise en œuvre de l'initiative phare par le biais de partenaires multilatéraux soit différente du concept initial de C-VAC, les principaux pays de travail ne changeront pas et l'impact de la programmation devrait être les mêmes. L'initiative continue de présenter une réelle opportunité de contribuer à des avancements concrets pour la couverture vaccinale COVID-19 dans vos pays d'accréditation. Le document ci-joint donne un aperçu des options de programmation qui seront poursuivies.

Comme prochaine étape, Megan Cain et l'équipe FMNV engageront les partenaires de l'Accélérateur ACT pour concevoir des options de programmation. Chaque pays aura son propre plan de programmation, tel qu'il a été conçu à l'origine, avec un financement affecté. L'équipe FMNV proposera des éléments programmatiques basés sur les analyses de pays fournies par vos équipes, en consultations avec les partenaires multilatéraux de mise en œuvre. **Ces plans de programmation par pays seront envoyés à vos équipes pour validation et commentaires d'ici le 30 mai 2022.** Tandis que FMNV développe les plans de programmation, nous vous demandons de commencer à identifier des opportunités au niveau national pour le leadership politique canadien - incluant la participation de MINE aux réunions nationales, aux forums régionaux ou mondiaux (par exemple, visites sur le terrain et voyages à venir), ainsi que des occasions opportunes pour les cadres supérieurs de la mission de faire participer les pays, les donateurs et les partenaires aux efforts de déploiement des vaccins du Canada dans le cadre de cette initiative.

Comme toujours, si vous avez des questions, n'hésitez pas à contacter la directrice du groupe de travail, Megan Cain, ou DD Mellissa Hisko.

Merci de votre coopération continue dans cette initiative en évolution.

Joshua

From: Tabah, Joshua -MND <Joshua.Tabah@international.gc.ca>
Sent: April 26, 2022 1:53 PM
Subject: COVID response: Budget 2022 and C-VAC update

GAC - Released under the Access to Informations Act/
AMC - Divulgué en vertu de la loi sur l'accès à l'information

Colleagues,

I'm reaching out as a follow-up to the release of Budget 2022 and the $732 million in 2022-23 identified for international assistance at GAC, and specifically on the implications for the Department's COVID-19 Vaccine Action Plan Canada (C-VAC) initiative.

Budget 2022: Per the language of the Budget, the full $732 million for COVID response is to go through ACT-A partners, ie. all multilaterals. GAC was additionally awarded funding for Global Health Security, but this was also subsequently confirmed to be specifically for an upcoming replenishment. The additional funds we had proposed, for more flexible COVID response programming and for increased pandemic preparedness investments, was not retained. Finally, note that while the $732M was initially communicated via the Budget, we expect the government will choose to formally "announce" this during the US/Biden Summit on COVID-19 on May 12th. As a result, we ask that you avoid any public communications about this until that time.

Implications for C-VAC: We are grateful to you and your teams for the collaboration on this country-level vaccine delivery initiative. It remains highly relevant: with immunization levels very low in many developing countries and with vaccines now going to waste because of lack of absorption capacity and demand, increasing support for delivery is the top priority in the international vaccine space. For us, given the Budget result, the main thrust of C-VAC implementation must now come via our support through the ACT-Accelerator, and therefore the 9 multilateral agencies that make up ACT-A (World Health Organization, UNICEF, Gavi, CEPI, Global Financing Facility, the Global Fund, FIND, Unitaid, the Wellcome Trust, the World Bank). We remain committed to C-VAC, but obviously need to significantly adapt and adjust proposed programming and activities to fit into this narrower set of funding parameters, in particular by earmarking support to relevant partners for specific lines of effort in targeted countries, and then communicating this out to your teams to allow for this be leveraged and amplified.

We have begun preliminary thinking on this, with a next step to engage DME and MINE for their direction. We will continue to keep you up to date as we learn more and as we begin to finalize ACT-A allocation decisions. Note that we have not yet had discussions regarding the potential for programs to use any remaining spending flexibility from their global health targets on local vaccine delivery/absorption issues to supplement these multilateral C-VAC allocations, but please let us know if this is something you are considering.

The fMNV team, led by Megan Cain, and I remain available to discuss further as required.

Joshua

***

Chers collègues,

GAC - Released under the Access to Informations Act/
AMC - Divulgué en vertu de la loi sur l'accès à l'information

Je vous contacte dans le cadre du suivi de la publication du Budget 2022 et les 732 millions de dollars identifiés pour l'aide internationale à AMC en 2022-2023, et plus particulièrement sur les implications pour l'initiative du Plan d'action pour les vaccins contre la COVID-19 (C-VAC).

Budget 2022: Selon le budget, la totalité des 732 millions de dollars pour la réponse COVID doit passer par les partenaires ACT-A, c'est-à-dire, par voie multilatérale. AMC a également reçu un financement pour la sécurité sanitaire mondiale, mais cela a également été confirmé par la suite comme étant spécifiquement destiné à un prochain réapprovisionnement. Les fonds supplémentaires que nous avions proposés, pour une programmation plus flexible de la réponse à la COVID et pour des investissements accrus dans la préparation à la pandémie, n'ont pas été retenus. Enfin, notez que si les 732 millions de dollars ont été initialement communiqués via le budget, nous nous attendons à ce que le gouvernement choisisse de « l'annoncer » formellement lors du sommet États-Unis/Biden sur la COVID-19 le 12 mai. Par conséquent, nous vous demandons d'éviter toute communication publique à ce sujet d'ici-là.

Implications pour C-VAC : Nous vous sommes reconnaissants, ainsi qu'à vos équipes, pour votre collaboration à cette initiative de distribution de vaccins au niveau national. Elle reste très pertinente : avec des niveaux de vaccination très bas dans de nombreux pays en développement et avec des vaccins qui sont désormais gaspillés en raison du manque de capacité d'absorption et de demande, l'augmentation du soutien à la livraison est la priorité absolue dans l'espace vaccinal international. Pour nous, compte tenu du résultat budgétaire, l'essentiel de la mise en œuvre de C-VAC doit désormais passer à travers l'ACT-Accelerator, et les 9 agences multilatérales qui composent ACT-A (Organisation Mondiale de la Santé, UNICEF, Gavi, CEPI , Mécanisme de financement mondial, Fonds mondial, FIND, Unitaid, Wellcome Trust, Banque mondiale). Nous restons attachés à C-VAC, mais nous devons évidemment adapter et ajuster de manière importante la programmation et les activités proposées pour s'adapter à cet ensemble plus restreint de paramètres de financement, en particulier en attribuant un soutien aux partenaires concernés pour des efforts spécifiques dans des pays spécifiques, puis en communiquant cela à vos équipes pour permettre à cela d'être optimisé et amplifié.

Nous avons commencé une réflexion préliminaire à ce sujet, avec une prochaine étape pour engager DME et MINE pour direction. Nous continuerons à vous tenir au courant au fur et à mesure que nous en apprendrons davantage et que nous commencerons à finaliser les décisions d'attribution. Notez que nous n'avons pas encore eu de discussions concernant la possibilité pour les programmes d'utiliser toute flexibilité de dépenses restante pour des objectifs de santé mondiale sur les problèmes de livraison / absorption de vaccins locaux pour compléter ces allocations multilatérales C-VAC, mais veuillez nous faire savoir si c'est quelque chose que vous envisagez.

GAC - Released under the Access to Informations Act/
AMC - Divulgué en vertu de la loi sur l'accès à l'information

L'équipe fMNV, dirigée par Megan Cain, et moi-même, restons disponibles pour discuter davantage si nécessaire.

Joshua

Joshua Tabah

Director General – Health and Nutrition
Directeur général – Santé et nutrition
Global Issues and Development Branch | Secteur des enjeux mondiaux et du développement

Tel: 343 203 6241; 343 998 8297 (m)
111 Sussex Drive, Ottawa, ON K1A 0G2
Global Affairs Canada | Affaires mondiales Canada
Government of Canada | Gouvernement du Canada

GAC - Released under the Access to Informations Act/
AMC - Divulgué en vertu de la loi sur l'accès à l'information

s.21(1)(a)
s.21(1)(b)

Canada's Global Initiative for Vaccine Equity (CanGIVE): Supporting COVID-19 Vaccine Roll-Out Programming Proposal

Canada is responding to the call for catalytic surge capacity to support vaccine roll-out in countries with high vaccine-related needs. Canada's Global Initiative for Vaccine Equity (CanGIVE) is a signature, whole-of-department initiative to support country-led efforts that enhance COVID-19 vaccine delivery, demand and production.

Informed by country level analysis in CanGIVE priority countries, this document outlines the proposed programming options for CanGIVE implementation, [redacted] Canada's programming will provide earmarked, country-specific support through ACT-Accelerator partners. All CanGIVE programming will contribute to meeting Canada's ACT-Accelerator burden share consistent with Budget 2022, through a collaborative approach with Canadian missions in priority countries[1] to scale-up the three CanGIVE pillars of vaccine delivery, demand and production. Complementary country advocacy engagement plans will follow to leverage these investments for maximum impact and to ensure Canadian visibility, while also reinforcing a "one plan" approach at country level with key partners (host governments, COVAX, USAID, Germany, etc.).

Partner	Project Description	Funding Amount
**Enhancing Vaccine Delivery and Increasing Demand**		
UNICEF	• Canada will support UNICEF to bolster vaccine logistics, supply chains, and health workforce while also undertaking demand generation activities and surge vaccination campaigns in 8 to 10 CanGIVE countries, primarily in Africa, with low vaccination rates and absorptive capacity.<br>• Country specific funding levels will be determined with UNICEF according to each countries' needs. Programming will complement a general basket of COVAX Delivery Support funds provided by Canada to Gavi and UNICEF.	[redacted]
UNICEF WFP/ UNHCR	• Working through UNICEF and other UN humanitarian partners to pilot nexus humanitarian programming alongside COVID-19 vaccination campaigns in 1 or 2 countries to increase efficiency of vaccination delivery and enhance vaccine confidence amongst displaced populations.	[redacted]
PAHO	• Building on Canada's existing COVID-19 response with the Pan-American Health Organization (PAHO), provide funding to PAHO for increased support for vaccine delivery in the Latin America and Caribbean region, specifically including CanGIVE countries Haiti, Jamaica and Colombia.	[redacted]

[1] [redacted]

GAC - Released under the Access to Informations Act/
AMC - Divulgué en vertu de la loi sur l'accès à l'information

	• Programming will also include regional support aimed at increasing vaccination amongst Venezuelan and other refugee and migrant populations.	
WHO	• Provide funding to WHO to strengthen vaccine delivery elements of health systems, including identifying needs and gaps in vaccine roll-out, facilitating partner coordination in country, information campaigns to target misinformation and increase vaccine confidence, and monitoring of country progress against vaccination targets.	[redacted]
**Increasing Vaccine Production**		
WHO/ MPP	• Increase support to the COVAX mRNA Technology Transfer Hub Programme in South Africa in support of phase III clinical trials, as well as start-up grants [redacted]	[redacted]
PAHO	• Support for PAHO's regional manufacturing platform and provide targeted assistance to scale-up vaccine production capacity in the Latin American region, including CanGIVE priority countries and COVAX mRNA Hub Programme recipient countries.	[redacted]
	**TOTAL BUDGET**	[redacted]

s.21(1)(a)

s.21(1)(b)

GAC - Released under the Access to Informations Act/
AMC - Divulgué en vertu de la loi sur l'accès à l'information

Canada's Global Initiative for Vaccine Equity (CanGIVE)

Canada is responding to the call for catalytic surge capacity to support vaccine roll-out and increase demand in countries with high COVID-19 vaccine-related needs. **Canada's Global Initiative for Vaccine Equity (CanGIVE)** is a signature initiative to support country-led efforts that enhance COVID-19 vaccine delivery, demand and production with a view to sustainable health systems strengthening in 13 countries. Informed by country level analysis, ACT-Accelerator Partners UNICEF, WHO, PAHO and Medicines Patent Pool (MPP) will deliver complementary, earmarked programming that contributes to Canada's ACT-Accelerator burden share, consistent with Budget 2022.

- **UNICEF** will lead efforts to bolster COVID-19 vaccine logistics, supply chains as well as strengthen the health workforce and scale-up targeted demand generation in 12 countries. Programming will include a focus on increased access for vulnerable populations, including a specific focus on bundling humanitarian and COVID-19 response in Bangladesh and Jordan.
- **WHO** will coordinate vaccine delivery support down to the local context in 5 African countries, including integration of COVID-19 response within primary health care and immunization.
- **PAHO** will increase access to COVID-19 vaccination through strengthened health systems, surveillance, and community outreach in 3 Latin American countries, alongside complementary flexible regional support. PAHO will also seek to expand manufacturing capacities and an enabling environment for local production in the region.
- **MPP** will support the sustainability of mRNA Vaccine Technology Transfer Hub in South Africa alongside bespoke catalytic funding for Hub tech transfer recipient manufacturing facilities in [redacted] countries.

CanGIVE Program Allocations – *in millions of Canadian dollars*

Country	UNICEF	PAHO	WHO	MPP	TOTAL
[redacted]					

CanGIVE will reinforce these investments for maximum impact through strategic country-level advocacy and political engagement led by Canada's Minister of International Development and senior missions staff to ensure Canadian visibility and reinforce a "one plan" approach at country level with the COVAX Vaccine Delivery Partnership (CoVDP).

[1] WHO and MPP country-specific program allocations are in final negotiations. Part of the WHO allocation ($2.4M) is currently being finalized.

s.21(1)(a)

s.21(1)(b)

GAC - Released under the Access to Informations Act/
AMC - Divulgué en vertu de la loi sur l'accès à l'information

Schulz, Sara -MNV [She,Her | Elle]

From: Tarr, Michael -FMNV
Sent: July 25, 2022 9:29 AM
To: Schulz, Sara -FMNV
Subject: RE: Meet with MPP this morning

Sounds good. I'm happy to run this by Charles if it's easier to keep your other meeting in place.

Glad you're fresh!

From: Schulz, Sara -FMNV <Sara.Schulz@international.gc.ca>
Sent: Monday, July 25, 2022 9:17 AM
To: Tarr, Michael -FMNV <Michael.Tarr@international.gc.ca>
Subject: RE: Meet with MPP this morning

Was just emailing you about this!
[redacted] this morning and getting caught up on emails. Thanks for the heads up on this one.

I don't necessarily have to be part of the meeting, although I would have to move our vaccines team Monday priorities meeting to join. Let me get back to you on that asap.

My only questions on the funding were to ask if our notional amounts for the support to recipient facilities are acceptable to MPP following whatever feedback on budgets they received from countries. For CanGIVE, we had notionally put this amount against the following countries:

[redacted]

Will confirm shortly if I can move our team meeting to join.

Sara

From: Tarr, Michael -FMNV <Michael.Tarr@international.gc.ca>
Sent: July 25, 2022 9:09 AM
To: Schulz, Sara -FMNV <Sara.Schulz@international.gc.ca>
Subject: Meet with MPP this morning

Hi Sara,

[redacted]

Just quickly on the MPP meet you'll see in your calendar for 11am this morning – Charles had reached out to discuss next funding and I asked him to include you on the invite as I know you were hoping to loop back with them. That said, I know you're unlikely able to attend, so I'm happy to fill you in afterwards and raise any questions, etc., from your side (just send a few bullet points my way).

Look forward to catching up at some point this week,
Mike

GAC - Released under the Access to Informations Act/
AMC - Divulgué en vertu de la loi sur l'accès à l'information

s.15(1) - International

s.21(1)(b)

July 25
Charles & Monica

Can-GIVE nominal allocations:

- [redacted]

[redacted]

Notes:

- [redacted]
- Timeline: need to confirm rough timeline of when funds would be disbursed (18 months from what date). This could determine which spoke countries are included and at what level.
- Spoke funding would go to regulatory work (training from South Africa regulatory group) as well as 'continental' training hub ([redacted]

s.15(1) - International

s.21(1)(b)

GAC - Released under the Access to Informations Act/
AMC - Divulgué en vertu de la loi sur l'accès à l'information

Schulz, Sara -MNV [She,Her | Elle]

From: Hisko, Mellissa -FMNV
Sent: August 11, 2022 7:29 AM
To: Schulz, Sara -FMNV
Subject: Re: For decision - MPP project
Attachments: MPP project questions.docx

Agree with all points and come back to me with more info [redacted]

Sent from my iPhone

On Aug 10, 2022, at 4:53 PM, Schulz, Sara -FMNV <Sara.Schulz@international.gc.ca> wrote:

Hey Mellissa,
A few questions below in red that I'd like to check with you on the MPP project for Mike to move ahead in the negotiations with MPP:

<u>Background:</u>

- [redacted]
- [redacted]

<u>Key points from MPP meeting (July 25):</u>

- [redacted]
- [redacted]
- [redacted]
- [redacted]
- [redacted]
- [redacted]

<u>Questions:</u>

- [redacted]
- [redacted]

s.21(1)(b)

GAC - Released under the Access to Informations Act/
AMC - Divulgué en vertu de la loi sur l'accès à l'information

- [redacted]

From: Tarr, Michael -FMNV <Michael.Tarr@international.gc.ca>
Sent: July 29, 2022 6:04 PM
To: Hisko, Mellissa -FMNV <mellissa.hisko@international.gc.ca>
Cc: Schulz, Sara -FMNV <Sara.Schulz@international.gc.ca>; Bender, Tracey -FMNV <Tracey.Bender@international.gc.ca>
Subject: Regional manufacturing update

Hi Mellissa,

Please find below and attached a few items on the regional manufacturing file:

- **For follow-up**: MPP project questions (attached). The partner has asked for some direction in order to come back with a revised concept note that specifies spoke countries and budgets. These may be simple Y/N answers or require more consideration/information (have discussed briefly with Sara). Not urgent – we can follow up by mid-August.

- **For awareness**: Update for management (below). Picking up where Sara left off, I will aim to produce these on a quarterly basis.

Thx,
Mike

***

Internal

- On July 25, fMNV/Tarr met with Charles Gore, MPP Executive Director, to discuss mRNA hub program developments and the concept note for the second tranche of Canada's funding ([redacted]
- On June 22, Canada (PRET/Khawam) attended a hub site visit in Cape Town as part of a larger Unitaid/MPP field visit for donors.
- In June, mRNA Hub project was submitted for consideration for potential MINE travel to Africa (Fall 2022).
- Ongoing communication with N-Branch on the PAHO regional manufacturing project. PAHO has now provided a second draft of concept note, with more detailed activities and budget.
- Initial contact from colleagues to explore how regional manufacturing might fit into the next phase of Canada's Biomanufacturing and Life Sciences Strategy. Further discussion needed.

International

- WTO: In June, WTO members reached a consensus decision on TRIPS to waive certain IP protections for COVID-19 vaccines. It is not clear the decision will have any tangible benefit for the regional manufacturing of vaccines due to waiver exclusions.
- G7: Support for regional manufacturing has been identified among actions in the *Pact for Pandemic Readiness* that aligns efforts in surveillance and rapid response as well as the

s.15(1) - International

s.21(1)(b)

GAC - Released under the Access to Informations Act/
AMC - Divulgué en vertu de la loi sur l'accès à l'information

Partnership for Global Infrastructure (June 2022 launch) that aims to mobilize $600B in public and private investment in LMICs over the next five years.
- Institut Pasteur de Dakar in Senegal: In June, one of the largest vaccine manufacturing projects in Africa received EUR $75M from the European Investment Bank. Construction has begun for a new facility that will produce up to 300M doses of COVID-19 and other vaccines annually for use across Africa.
- BioNTech: A high-profile ground-breaking ceremony for the company's first vaccine production facility in Africa was held during the Commonwealth Heads of State Meeting in Rwanda in June. The facility is based on two modular containers (BioNTainers) that will produce a range of mRNA-based vaccines for the needs of African countries.

mRNA Hub Program
- Funding: [redacted]
- Spoke development: Discussions and planning is under way for portfolio of vaccine candidates as well as budget development for financial and technical support requirements. Introductory training has been provided to Brazil, Argentina, Bangladesh and Indonesia spokes. Upcoming training for India and Egypt.
- Workforce training: While initially selecting South Korea for a global training hub, the program is now looking to develop several regional workforce training hubs (5-6 in total).
- Partnerships: [redacted]
- Events: MPP participation at AIDS2022 in a roundtable discussion on the mRNA technology transfer hub program (July 31).

Media
- POLITICO: Scaling up Africa's vaccine manufacturing easier said than done (link).
- Nature: Why a vaccine hub for low-income countries must succeed (link).
- Nature: The radical plan for vaccine equity (link)
- The Atlantic: The power imbalance undermining global health (link)

Comments:
- Regional manufacturing continues to see increasing interest from regional bodies, LMIC and donor countries, media, and CSOs. The issue is now firmly embedded within WHO, G7, and G20 policy tracks on pandemic prevention, preparedness and response.
- [redacted]
- While significant political attention and funding is resulting in a growing number of manufacturing initiatives, there is general view that not all will be successful or sustainable due to factors such as IP barriers, predictable demand, and long-term procurement. There is also a growing presence of manufacturers from the global north setting up production capacity in LMICs, particularly in Africa.
- Gavi, [redacted] release of a white paper (A New Era of Vaccine Manufacturing in Africa), dialogue with stakeholders in the space, the creation of a dedicated position (link), and the inclusion of regional manufacturing in Gavi

GAC - Released under the Access to Informations Act/
AMC - Divulgué en vertu de la loi sur l'accès à l'information

s.21(1)(b)

5.1 discussions. [redacted]

GAC - Released under the Access to Informations Act/
AMC - Divulgué en vertu de la loi sur l'accès à l'information

Tarr, Michael -MNV [He,Him | Il]

From: Monica Moschioni [redacted]
Sent: September 12, 2022 8:56 AM
To: Tarr, Michael -FMNV [He,Him | Il]
Cc: Charles Gore; Jane Caldwell
Subject: RE: Budget
Attachments: MPP budget_fund request_Canada_Jun2022_final_modif 12Sep2022.docx; MPP budget_fund request_Canada_Jun2022_final_modif 12Sep2022.pdf

Follow Up Flag: Follow up
Flag Status: Completed

Dear Michael,
PFA the modified budget where we have broken down the Overheads [redacted]

Please let s know if there are questions.
Kind regards
Monica

From: Michael.Tarr@international.gc.ca <Michael.Tarr@international.gc.ca>
Sent: Wednesday, 7 September 2022 19:45
To: Monica Moschioni [redacted]
Cc: Charles Gore <[redacted] Jane Caldwell <[redacted]
Subject: RE: Budget

Hi Monica,

Thanks for the update – absolutely fine for early next week.

Appreciate the effort to track down this info.

Best,
Mike

From: Monica Moschioni <[redacted]
Sent: September 7, 2022 7:13 AM
To: Tarr, Michael -FMNV [He,Him | Il] <Michael.Tarr@international.gc.ca>
Cc: Charles Gore <[redacted] Jane Caldwell <[redacted]
Subject: RE: Budget

Hi Michael,
To respond more clearly to your request, we have checked with Biovac and SAMRC.
[redacted]
SAMRC situation is not fully clear and we might be able to get a better view early next week. Would it be ok if we come back to you on Monday/Tuesday next week?

GAC - Released under the Access to Informations Act/
AMC - Divulgué en vertu de la loi sur l'accès à l'information

s.19(1)

s.20(1)(c)

s.21(1)(b)

Just to add further clarity, MPP overheads are not included in the budget.

I have added the head of MPP fin dept in CC.

Thx a lot
Monica

From: Michael.Tarr@international.gc.ca <Michael.Tarr@international.gc.ca>
Sent: Tuesday, 6 September 2022 19:55
To: Monica Moschioni <[redacted]
Cc: Charles Gore [redacted]
Subject: RE: Budget

Hi Monica,

Thanks for quick reply. That's correct – if possible to separate out the overheads into that line would be much appreciated.

Thanks kindly,
Mike

From: Monica Moschioni <[redacted]
Sent: September 6, 2022 1:53 PM
To: Tarr, Michael -FMNV [He,Him | Il] <Michael.Tarr@international.gc.ca>
Cc: Charles Gore <[redacted]
Subject: RE: Budget

HI Michael,

[redacted]

Thx a lot.

Monica

GAC - Released under the Access to Informations Act/
AMC - Divulgué en vertu de la loi sur l'accès à l'information

s.19(1)

s.20(1)(c)

s.21(1)(b)

[redacted]

From: Michael.Tarr@international.gc.ca <Michael.Tarr@international.gc.ca>
Sent: Tuesday, 6 September 2022 17:24
To: Monica Moschioni <[redacted]
Cc: Charles Gore <[redacted]
Subject: FW: Budget

Hi Monica,

A quick follow-up on the draft budget (attached).

Question from our finance on the indirect/overhead costs - [redacted]

Thanks for the clarification,
Mike

From: Monica Moschioni <[redacted]
Sent: August 16, 2022 9:40 AM
To: Tarr, Michael -FMNV <Michael.Tarr@international.gc.ca>
Cc: Charles Gore <[redacted]
Subject: RE: Follow up

Hi Michael,
PFA our draft budget (word and PDF).
Should you have question, please do not hesitate to contact us.
Best regards

Monica

From: Michael.Tarr@international.gc.ca <Michael.Tarr@international.gc.ca>
Sent: Monday, 15 August 2022 15:42
To: Monica Moschioni <[redacted]

GAC - Released under the Access to Informations Act/
AMC - Divulgué en vertu de la loi sur l'accès à l'information

s.19(1)

s.21(1)(b)

Cc: Charles Gore <[redacted]
Subject: RE: Follow up

Hi Monica,

Hope you had a nice weekend there.

I've been requested by our finance department to provide a draft budget along with the existing concept note. This is a working budget only – it doesn't lock MPP into any spending breakdown. We can refine the budget and selected countries later, when the grant agreement is completed.

I've attached a rough template that would be sufficient level of detail required at this point. Would you be able to provide this by mid-week? My apologies for the tight turnaround, and please let me know if you have any questions or concerns.

Best,
Mike

From: Tarr, Michael -FMNV
Sent: August 12, 2022 9:42 AM
To: 'Monica Moschioni' <[redacted]>
Cc: Charles Gore <[redacted]
Subject: RE: Follow up

Hi Monica,

Thanks kindly for the quick response. A few comments below.

Wishing you a very nice weekend,
Mike

Spoke countries

- [redacted]
- Bangladesh – we are fine to support a private-only company so long as the same sustainability and equity approach applies as per the other spokes.
- Request – could you provide some information on the selection process for the spokes (general language or screening criteria)? For due diligence we need to demonstrate the managerial, financial and technical expertise of implementing agencies. Instead of doing this for each individual spoke facility, we're hoping there's a blanket solution via the selection process. → selection was made by WHO (PDVAC) following an EOI process. Will come back to you with some language. Excellent – thank you!

Budget

- We would be fine to support the regulatory and workforce training hub components, understanding this will involve travel, salary and/or consultancy costs. → would it be for all the countries or the selected ones? All the

GAC - Released under the Access to Informations Act/
AMC - Divulgué en vertu de la loi sur l'accès à l'information

s.19(1)

s.21(1)(b)

countries as needed – [redacted]

- Question – will the spoke support funding sit with and be spent by MPP? Or will funds be transferred into separate budgets held by spokes (similar to Biovac)? → to discuss. [redacted]

Timeframe → We are currently reviewing fundings timelines vs budgets vs cashflow. Will come back to you. **Perfect.**

- [redacted]
- [redacted] This is another level of finance complexity but we'd be happy to go this route if it'll provide the runway needed for spoke implementation and success. Let me know your thoughts on whether we should pursue this.

From: Monica Moschioni <[redacted]
Sent: August 12, 2022 4:04 AM
To: Tarr, Michael -FMNV <Michael.Tarr@international.gc.ca>
Cc: Charles Gore <[redacted]
Subject: RE: Follow up

Hi Michael,
Thx for coming back to us.
[redacted]. We will discuss together all the points below (some require WHO input as well) once he will be back in the office (in about 10 days) and will come back to you.
Few questions comments in red below from my side.

Have a nice day
Best regards

Monica

From: Michael.Tarr@international.gc.ca <Michael.Tarr@international.gc.ca>
Sent: Thursday, 11 August 2022 22:18
To: Charles Gore <[redacted]>; Monica Moschioni <[redacted]
Subject: Follow up

Dear Charles and Monica,

Hope you're both doing well [redacted]

Thanks for making space to meet a few weeks ago. We've discussed internally and have a few confirmations, questions and requests below:

Spoke countries

- [redacted]

GAC - Released under the Access to Informations Act/
AMC - Divulgué en vertu de la loi sur l'accès à l'information

- Bangladesh – we are fine to support a private-only company so long as the same sustainability and equity approach applies as per the other spokes.
- Request – could you provide some information on the selection process for the spokes (general language or screening criteria)? For due diligence we need to demonstrate the managerial, financial and technical expertise of implementing agencies. Instead of doing this for each individual spoke facility, we're hoping there's a blanket solution via the selection process. → selection was made by WHO (PDVAC) following an EOI process. Will come back to you with some language.

Budget

- We would be fine to support the regulatory and workforce training hub components, understanding this will involve travel, salary and/or consultancy costs. → would it be for all the countries or the selected ones?
- Question – will the spoke support funding sit with and be spent by MPP? Or will funds be transferred into separate budgets held by spokes (similar to Biovac)? → to discuss. [redacted]

Timeframe → We are currently reviewing fundings timelines vs budgets vs cashflow. Will come back to you.

- [redacted]
- [redacted] This is another level of finance complexity but we'd be happy to go this route if it'll provide the runway needed for spoke implementation and success. Let me know your thoughts on whether we should pursue this.

If you could revert on the questions and request above at earliest convenience, would be very appreciative.

Otherwise, please let me know if I've missed anything or whether this additional information is sufficient for a refined concept note. Ideally we would aim to have the next version by early-mid September.

Best,
Mike

GAC - Released under the Access to Informations Act/
AMC - Divulgué en vertu de la loi sur l'accès à l'information

s.15(1) - International
s.21(1)(b)

MPP Funding request_Draft budget_12Sep2022

Draft budget

Indicative figures only

1- Continued support of technology transfer activities South African Hub (Afrigen) and spoke (Biovac)	
• GMP (R&D) COVID-19 mRNA vaccine manufacturing process and analytical methods development<br>• mRNA vaccine batch production and testing for a Phase I clinical trial	
• GMP COVID-19 mRNA vaccine manufacturing process scale-up and process and analytical methods validation<br>• mRNA vaccine batches production and testing for a Phase III clinical trial	
• Develop a second-generation mRNA vaccine technology and establish a pipeline of vaccine candidates for Low- Middle-Income Countries	
2- Additional support for technology transfer activities across the spoke network	
• Support to local regulatory authorities to achieve Maturity level 3 for vaccines	
• Training of local workforce in GMP manufacturing, vaccinology, mRNA technology	
• Technology transfer support to spokes (including technical support before and during technology transfer, equipment and consultancy support on models of sustainability)	
Total Direct Costs	
Indirect Cost (overhead)	
Total estimated budget	

s.15(1) - International

s.21(1)(b)

GAC - Released under the Access to Informations Act/
AMC - Divulgué en vertu de la loi sur l'accès à l'information

Canada's Global Initiative for Vaccine Equity (CanGIVE)

Canada is responding to the call for catalytic surge capacity to support vaccine roll-out and increase demand in countries with high COVID-19 vaccine-related needs. **Canada's Global Initiative for Vaccine Equity (CanGIVE)** is a signature initiative to support country-led efforts that enhance COVID-19 vaccine delivery, demand and production with a view to sustainable health systems strengthening in 12 countries. Informed by country level analysis, ACT-Accelerator Partners UNICEF, WHO, PAHO and Medicines Patent Pool (MPP) will deliver complementary, earmarked programming that contributes to Canada's ACT-Accelerator burden share, consistent with Budget 2022.

- **UNICEF** will lead efforts to bolster COVID-19 vaccine logistics, supply chains as well as strengthen the health workforce and scale-up targeted demand generation in 11 countries. Programming will include a focus on increased access for vulnerable populations, including a specific focus on bundling humanitarian and COVID-19 response in Bangladesh and Jordan.
- **WHO** will coordinate vaccine delivery support down to the local context in 5 African countries, including integration of COVID-19 response within primary health care and immunization.
- **PAHO** will increase access to COVID-19 vaccination through strengthened health systems, surveillance, and community outreach in 3 Latin American countries, alongside complementary flexible regional support. PAHO will also seek to expand manufacturing capacities and an enabling environment for local production in the region.
- **MPP** will support the sustainability of mRNA Vaccine Technology Transfer Hub in South Africa alongside bespoke catalytic funding for Hub tech transfer recipient manufacturing facilities in [redacted] countries.

CanGIVE Program Allocations – *in millions of Canadian dollars*

Country	UNICEF	PAHO	WHO	MPP	TOTAL
[redacted]					

CanGIVE will reinforce these investments for maximum impact through strategic country-level advocacy and political engagement led by Canada's Minister of International Development and

GAC - Released under the Access to Informations Act/
AMC - Divulgué en vertu de la loi sur l'accès à l'information

senior missions staff to ensure Canadian visibility and reinforce a "one plan" approach at country level with the COVAX Vaccine Delivery Partnership (CoVDP).

s.21(1)(b)

GAC - Released under the Access to Informations Act/
AMC - Divulgué en vertu de la loi sur l'accès à l'information

Martin, Christine -MNV [She,Her | Elle]

From: Martin, Christine -FMNV [She,Her | Elle]
Sent: October 13, 2022 2:56 PM
To: Schulz, Sara -FMNV
Subject: proposal for $117M funding allocation

Hi Sara,

Based on conversations we had on Friday, here are some suggestions on potential funding allocations to support COVID-19 vaccination efforts. Noting that providing additional funds would require a project timeline extension. The proposed amounts assume a 24month period.

[redacted]

Happy to chat!
Christine

[redacted]

**$117M**	**TOTAL**

Christine Martin (she/her/hers)
Analyst | Analyste
COVID-19 Global Health Response Task Force (fMNV) | Groupe de travail de la réponse sanitaire mondiale de la COVID-19 (fMNV)
Health and Nutrition Bureau (MND) | Direction générale de la Santé et de la Nutrition (MND)
: christine.martin@international.gc.ca
: 343-543-7913
: chat with me | clavardez avec moi

111 Promenade Sussex/ Promenade Sussex, Ottawa, Ontario, K1N 1J1
Global Affairs Canada | Affaires mondiales Canada
Government of Canada | Gouvernement du Canada

GAC - Released under the Access to Informations Act/
AMC - Divulgué en vertu de la loi sur l'accès à l'information

Tarr, Michael -MNV [He,Him | Il]

From: Schulz, Sara -FMNV
Sent: October 24, 2022 1:15 PM
To: Bernadotte, Maeva -NLG; Tarr, Michael -FMNV [He,Him | Il]; Martin, Christine -FMNV [She,Her | Elle]
Cc: Guimond, Alexandre -NLG; McDowell, Charlotte -PRMOAS -GR; Hisko, Mellissa -FMNV [She,Her | Elle]
Subject: Additional allocations for CanGIVE projects
Attachments: Due diligence tracker-10687673-SGDE-EDRMS.DOCX.DRF; SGDE-EDRMS-#10687673-v1-Due diligence tracker.DOCX

Importance: High

Hey there PTLs,
We have had preliminary discussions with S-branch and can now confirm a way forward regarding the additional allocations for the CanGIVE projects. I'll be coordinating this process and am sending everything in an email because everyone is so busy, but I'm very happy to jump on a call if that would help to answer any questions. The notional amounts tested with oMINE and approach suggested for each project are summarized in the table below for your validation:

SAP number	Partner	Project description	Status	Approach	Signed Project Value	Additional Allocation	New Total Value
(P011782001)	UNICEF	CanGIVE Vx delivery (scale-up activities in 11 countries)	Agreement sent to partner for signature – disbursal Oct 2022	Proceed with current agreement and first payment as planned. Create new sub-element and grant amendment with fund increase.	$110M	$60M	$170
(P011860001)	WHO	CanGIVE (scale-up activities in 5 countries/possible addition of Ghana/Mozambique)	Agreement sent to partner for signature – disbursal Oct 2022	Proceed with current agreement and payment as planned. Create new sub-element and grant amendment with fund increase.	$30	$27M	$57
(P011103002)	MPP	CanGIVE manufacturing hub (increase funding to spokes + core)	Amendment in process – waiting on Supps B	Add fund increase to amendment already in process.	$30	$15M	$45
(P011811001)	PAHO	CanGIVE Vx delivery (scale-up activities in 3 countries and regional flex funds)	Draft Agreement in process – waiting on Supps B	Add fund increase to new agreement already in process.	$30	$15M	$45

GAC - Released under the Access to Informations Act/
AMC - Divulgué en vertu de la loi sur l'accès à l'information

In summary, UNICEF and WHO grants will proceed with signature and payment on the nearly finalized agreements, and the fund increases will come through a formal amendment. For PAHO, please hold on the current agreement because the approach suggested by S-branch is to add the new increase amount into the current draft agreement. Similarly, for MPP, hold on the current amendment, and we will add the new funds in so that we only make one amendment for $30M.

In terms of next steps, a suggested critical path is below. Timing is real quick in order to get the memo in front of MINE before they wind down for the holidays. Due diligence will be simplified, given the major shift is only to budget amounts and project timelines, not project scope. We hope this will keep the process manageable for us all! We are currently targeting November 10th to complete all due diligence; I'm confirming this with S-branch and will revert on any changes to that timing. Please track progress in the attached EDRMS document. Maeva, feel free to send by email and I can update the EDRMS as required.

ACTIVITY	DEADLINE	*NOTES*
Projects batched in Batch #4	Oct 24	*Sara - Confirmed with MFMZ.*
PTLs engage partners to confirm allocations and program implications – request updated high-level budgets	Week of Oct 24	*Prioritize increases to support scale up within scope of current projects as much as possible.*
PTLs submit FRET Request and Transfer Payment intake form	Oct 25	*Sara to send advance email to S-branch confirming group of projects. PTLs Please use similar text and distribution list when submitting forms. Copy Sara.*
Memo to MND for approval	Oct 28	
Memo to oMFM for approval	Nov 3	
PTLS submit FRETs for data verification	Nov 3	*All projects: FRET update to RF8 and RF6c **only**. DG overrides required.*
All due diligence complete	Nov 10	*All projects: Email to Gender specialist confirming increase amount but no change to outcomes/GE elements (ask Christine for sample)*<br>*UNICEF and WHO – EIP required*<br>*MPP and PAHO – no EIP required. email to enviro spec confirming increased amount but no significant change in outcomes/activities*
Submit Memo and IA to DCB	Nov 10	
MINE signature	Dec 2	

GAC - Released under the Access to Informations Act/
AMC - Divulgué en vertu de la loi sur l'accès à l'information

Thanks everyone, definitely don't hesitate if you have any questions.

Best,
Sara

Sara Schulz
a/Deputy Director | Directrice adjointe en interim
COVID-19 Global Health Response Task Force (fMNV) | Groupe de travail de la réponse sanitaire mondiale de la COVID-19 (fMNV)
Global Health and Nutrition Platforms (MNC) | Plateformes de santé mondiale et de nutrition (MNC)
Health and Nutrition Bureau (MND) | Direction générale de la Santé et de la Nutrition (MND)
sara.schulz@international.gc.ca
Tel. : 343-548-9938

s.21(1)(a)

s.21(1)(b)

GAC - Released under the Access to Informations Act/
AMC - Divulgué en vertu de la loi sur l'accès à l'information

Tarr, Michael -MNV [He,Him | Il]

From: Tarr, Michael -MNV [He,Him | Il]
Sent: October 31, 2022 12:42 PM
To: Martin, Christine -MNV [She,Her | Elle]
Subject: RE: Spoke funding

Perfect, thanks Christine.

MPP provided a strong written assessment of IP risk and mitigation. I will enter this into the FREF.

From: Martin, Christine -MNV [She,Her | Elle] <Christine.Martin@international.gc.ca>
Sent: October 31, 2022 9:35 AM
To: Tarr, Michael -MNV [He,Him | Il] <Michael.Tarr@international.gc.ca>
Subject: RE: Spoke funding

I got big boss Sara's input:

Overall, she agrees with you and me (my comments were in red). This is what she shared, noting her question on IP, which we have discuss in the past but any update on the issue would be appreciated:

Agree with the suggestions below – ideally we would want 'scenario 2' to build on the already approved scenario 1

[redacted]

Also – did Mike feel good about Charles' responses on any IP challenges that could present in the near future?

- Need to change "stream" to "scenario"
- [redacted]

From: Tarr, Michael -MNV [He,Him | Il] <Michael.Tarr@international.gc.ca>
Sent: October 31, 2022 8:58 AM

s.21(1)(a)
s.21(1)(b)
GAC - Released under the Access to Informations Act/
AMC - Divulgué en vertu de la loi sur l'accès à l'information

To: Martin, Christine -MNV [She,Her | Elle] <Christine.Martin@international.gc.ca>
Subject: RE: Spoke funding

Hey Christine!

Just a quick check-in – any chance you can confirm this with Sara this morning?

From your reply on Friday, did you mean to indicate preferences below? (I don't see any). Anyhow, if you don't have any strong Can-GIVE preferences or needs, I suggest the following:

- [redacted]
-

Merci!
Mike

From: Martin, Christine -MNV [She,Her | Elle] <Christine.Martin@international.gc.ca>
Sent: October 28, 2022 4:07 PM
To: Tarr, Michael -MNV [He,Him | Il] <Michael.Tarr@international.gc.ca>
Subject: RE: Spoke funding

Hi Mike,

Thank you so much for this! I appreciate your mind and value your input. I think I have a preference (see below) but will run by Sara because her DD mind is no. 1.

Thank you for everything, you rock!
-Christine

From: Tarr, Michael -MNV [He,Him | Il] <Michael.Tarr@international.gc.ca>
Sent: October 28, 2022 10:36 AM
To: Martin, Christine -MNV [She,Her | Elle] <Christine.Martin@international.gc.ca>
Subject: FW: Spoke funding

Hey Christine,

Have gone through it in more detail, with some notes and questions below. Can you go review with Sara from a CAN-Give lens and let me know? Gracias!

- Need to change "stream" to "scenario"
- [redacted]
-

GAC - Released under the Access to Informations Act/
AMC - Divulgué en vertu de la loi sur l'accès à l'information

- [redacted]

From: Tarr, Michael -MNV [He,Him | Il]
Sent: October 28, 2022 10:05 AM
To: Martin, Christine -MNV [She,Her | Elle] <Christine.Martin@international.gc.ca>
Subject: FW: Spoke funding

Let me know if you have any thoughts, requests, hopes, dreams, complaints.

GAC - Released under the Access to Informations Act/
AMC - Divulgué en vertu de la loi sur l'accès à l'information

Tarr, Michael -MNV [He,Him | Il]

From: Tarr, Michael -MNV [He,Him | Il]
Sent: October 31, 2022 1:59 PM
To: 'Monica Moschioni'
Cc: Ike James; Charles Gore
Subject: RE: Spoke funding

Well received, thanks kindly Monica for managing to get this done today.

I'm good to proceed with this document (signature only required for the final grant agreement).

Will keep you posted this moves ahead quickly now.

Best,
Mike

From: Monica Moschioni [redacted]
Sent: October 31, 2022 1:53 PM
To: Tarr, Michael -MNV [He,Him | Il] <Michael.Tarr@international.gc.ca>
Cc: Ike James <[redacted]; Charles Gore <[redacted]
Subject: RE: Spoke funding

Hi Michael,
PFA the documents revised based on our discussions (xls and doc).

I was unable to get it signed today by Charles. If necessary, I will provide you (hopefully tomorrow) with a signed version.

Please let me know if further adjustments are needed.

Kind regards
Monica

From: Michael.Tarr@international.gc.ca <Michael.Tarr@international.gc.ca>
Sent: Monday, 31 October 2022 17:21
To: Monica Moschioni [redacted]
Subject: RE: Spoke funding

Hi Monica,

Can you chat in 10 mins? Feel free to send a Teams link. Talk then, Mike

GAC - Released under the Access to Informations Act/
AMC - Divulgué en vertu de la loi sur l'accès à l'information

From: Monica Moschioni <[redacted]
Sent: October 31, 2022 12:17 PM
To: Tarr, Michael -MNV [He,Him | Il] <Michael.Tarr@international.gc.ca>
Cc: Charles Gore <[redacted]; Ike James <[redacted]
Subject: RE: Spoke funding

Hi Michael,
Thx for the feedback.
Should you be available I think would be good to have a quick chat, to make sure the document is modified as you want. I'm available now. If you also can speak.

[redacted]

I will modify the document based on the feedback below.

Now from the below I understand:

[redacted]

DO you want to have the training money to be grouped and flexible across the countries? [as proposed below]?

GAC - Released under the Access to Informations Act/
AMC - Divulgué en vertu de la loi sur l'accès à l'information

[redacted]

Looking forward to hearing from you.
Kind regards
Monica

From: Michael.Tarr@international.gc.ca <Michael.Tarr@international.gc.ca>
Sent: Monday, 31 October 2022 16:42
To: Monica Moschioni [redacted]
Cc: Charles Gore <[redacted] Ike James <[redacted]
Subject: RE: Spoke funding

Hi Monica,

Thanks kindly for the updated budget and information on IP.

After reviewing internally, please see a few requested changes below. [redacted]

a) Language tweak (if you could change 'stream' to 'scenario' throughout the document):
   - [redacted]
   - [redacted]

b) Scenario B breakdown
   - [redacted]
   - [redacted]

s.15(1) - International

s.19(1)

GAC - Released under the Access to Informations Act/
AMC - Divulgué en vertu de la loi sur l'accès à l'information

c) Spoke countries (for both scenarios)
   - [redacted]

Please let me know if any questions. If possible to get revised document back to me today, would be greatly appreciated.

Thanks,
Mike

From: Monica Moschioni [redacted]
Sent: October 28, 2022 9:53 AM
To: Tarr, Michael -MNV [He,Him | Il] <Michael.Tarr@international.gc.ca>
Cc: Charles Gore <[redacted]; Ike James <[redacted]
Subject: RE: Spoke funding

Dear Michael,
How are you? [redacted]

As discussed on Wednesday, we have prepared a funding request with two funding streams.

- [redacted]
- [redacted]

As requested, in the funding request we have also added a detailed explanation of our strategy for IP.

The detailed breakdown of Funding stream A is present in the Annex 1 of the request and in the xls attached.
The detailed breakdown of Funding stream B is present in the Annex 2 of the request and in the xls attached.

The amounts are expressed in both CAD and USD in the xls, while in the request, for simplicity, we have reported only the CAD figures.

We have kept in one of the tabs in the xls also the [redacted] funding, so that you can see the overall rationale for the breakdown of Funding stream B for the spokes component.

[redacted]

We did not include the description of the activities that will be funded only [redacted] this time in the word document [you have them in the file I sent you last time].
Please do not hesitate to contact us if you have urgent questions.
I will keep on monitoring the e-mail this evening and during the WE.

Looking forward to hearing from you.
I wish you a nice WE
Best regards
Monica

GAC - Released under the Access to Informations Act/
AMC - Divulgué en vertu de la loi sur l'accès à l'information

s.19(1)

From: Michael.Tarr@international.gc.ca <Michael.Tarr@international.gc.ca>
Sent: Friday, 21 October 2022 15:49
To: Monica Moschioni [redacted]
Cc: Charles Gore [redacted]; Ike James <[redacted]
Subject: RE: Spoke funding

Thanks Monica, either Tuesday or Wednesday between 17 and 19 would work for me.

Wishing you a nice weekend,
Mike

From: Monica Moschioni [redacted]
Sent: October 20, 2022 12:08 PM
To: Tarr, Michael -FMNV [He,Him | Il] <Michael.Tarr@international.gc.ca>
Cc: Charles Gore <[redacted] Ike James [redacted]
Subject: RE: Spoke funding

Dear Michael,
These are our **availabilities CET time** for the next week:

Tuesday 25th Oct: 14-15, or 17-19
Wed 26th Oct: 17-19
Fri 28th Oct: 15-17

Please let us know if any of these fits your schedule.
Kind regards
Monica

From: Monica Moschioni
Sent: Thursday, 20 October 2022 08:57
To: Michael.Tarr@international.gc.ca
Cc: Charles Gore <[redacted]; Ike James [redacted]
Subject: RE: Spoke funding

Hi Michael,
thx for the feedback. We will be happy to meet.
I will align with Charles and come back to you with a couple of slots.
Have a nice day
Monica

From: Michael.Tarr@international.gc.ca <Michael.Tarr@international.gc.ca>
Sent: Wednesday, 19 October 2022 21:45
To: Monica Moschioni [redacted]
Cc: Charles Gore <[redacted] Ike James [redacted]
Subject: RE: Spoke funding

Hi Monica,

GAC - Released under the Access to Informations Act/
AMC - Divulgué en vertu de la loi sur l'accès à l'information

Apologies for delay – hoping we can schedule a 30 minute meet to discuss the breakdown and the funding gap.

Kindly let me know a few windows of availability on your side over the coming week or so.

Best,
Mike

From: Monica Moschioni <[redacted]
Sent: October 18, 2022 7:13 AM
To: Tarr, Michael -FMNV [He,Him | Il] <Michael.Tarr@international.gc.ca>
Cc: Charles Gore [redacted] Ike James <[redacted]
Subject: RE: Spoke funding

Hi Michael,
How are you?
Just wanted to inform you that we are moving forward with a the funding request to [redacted] by using the breakdown we shared with you and also would like to know if there is any feedback on the proposal you could share with us or you need further information.

Looking forward to hearing from you.
Have nice day
Monica

From: Michael.Tarr@international.gc.ca <Michael.Tarr@international.gc.ca>
Sent: Friday, 7 October 2022 15:17
To: Monica Moschioni <[redacted]
Cc: Charles Gore <[redacted] Ike James <[redacted]
Subject: RE: Spoke funding

Hi Monica,

Thanks kindly – will follow up shortly.

Best
Mike

From: Monica Moschioni <[redacted]
Sent: October 7, 2022 7:27 AM
To: Tarr, Michael -FMNV [He,Him | Il] <Michael.Tarr@international.gc.ca>
Cc: Charles Gore <[redacted]; Ike James <[redacted]
Subject: RE: Spoke funding

Hi Michael,
Please find below the answers to your questions below in yellow:

- What is the funding gap of the core budget (minus anticipated funds from [redacted] Canada, others)? → Overall, as per current core budget projections (117M USD), we have 47M USD gap (70M secured). [redacted] Canada funds (as per the allocation you received, still not approved) should contribute ~14M USD, thus reducing the funding gap to 33M USD. Other funding discussions ongoing are very preliminary. Hope this answers your question.

GAC - Released under the Access to Informations Act/
AMC - Divulgué en vertu de la loi sur l'accès à l'information

- Understand spoke pillar budget is still under assessment, however do you have a rough estimate of funds still required? (or simply confirmation that additional funding could be absorbed) → We did not have the possibility yet to make in depth gap assessments for the spokes. It will be our priority in the upcoming months. To respond to your question, additional funding can be absorbed. As you might have seen in the allocation explanation shared with you, the funding for equipment only partially covers the cost of basic equipment (no infrastructural adjustments, no HR support…).

Please let me know if you need further clarifications.
Cheers
Monica

From: Michael.Tarr@international.gc.ca <Michael.Tarr@international.gc.ca>
Sent: Friday, 7 October 2022 12:41
To: Monica Moschioni <[redacted]
Cc: Charles Gore <[redacted] Ike James <[redacted]
Subject: RE: Spoke funding

Hi Monica,

Well received, with many thanks. Will follow up shortly.

In the meantime, a few quick questions:

- What is the funding gap of the core budget (minus anticipated funds from [redacted] Canada, others)?
- Understand spoke pillar budget is still under assessment, however do you have a rough estimate of funds still required? (or simply confirmation that additional funding could be absorbed)

If possible to get back to me today, would be fantastic.

Also to pass along – our Director General very much enjoyed the side event at UNGA.

Best,
Mike

From: Monica Moschioni <[redacted]
Sent: October 5, 2022 12:35 PM
To: Tarr, Michael -FMNV [He,Him | Il] <Michael.Tarr@international.gc.ca>
Cc: Charles Gore <[redacted] Ike James <[redacted]
Subject: RE: Spoke funding

Hi Michael,

Based on the discussions below and taking into consideration yours [redacted] country preferences, we have broken down the [redacted] for the spokes ([redacted] as depicted in the xls file attached.
The .docx file explains how the amounts were calculated. We have added explanations for all the funding categories, for clarity (including those not matched with Canada).

As you will see in the file attached, the [redacted] funds for the spokes have been allocated to Equipment procurement for the spokes you chose and for training (your preferred countries and other countries).

GAC - Released under the Access to Informations Act/
AMC - Divulgué en vertu de la loi sur l'accès à l'information

As discussed during the call, overall, we have tried to prefer public companies for funding [private/public designation is also included in the file].

[redacted]

Should you have any question, please do not hesitate to contact us. Should you need a meeting to go through all, please let us know.

Based on your feedback, we will prepare a narrative for funding request.

Have a nice day
Monica

Monica Moschioni, PhD MPH | Project Manager Technology Transfer |Medicines Patent Pool
Rue de Varembé 7, 1202 Geneva, Switzerland
Mob: [redacted]
E-mail: [redacted]
Website | Newsletter | Twitter | LinkedIn

From: Michael.Tarr@international.gc.ca <Michael.Tarr@international.gc.ca>
Sent: Wednesday, 14 September 2022 17:10
To: Charles Gore <[redacted]
Cc: Monica Moschioni <[redacted]
Subject: RE: Spoke funding

Dear Charles,

Appreciate the challenge on your hands and the efforts to find a workable solution. From our side, no issue to share the overall plan showing Canada's contribution.

Best,
Mike

From: Charles Gore <[redacted]
Sent: September 14, 2022 4:56 AM
To: Tarr, Michael -FMNV [He,Him | Il] <Michael.Tarr@international.gc.ca>
Cc: Monica Moschioni <[redacted]
Subject: Spoke funding

Dear Mike,

GAC - Released under the Access to Informations Act/
AMC - Divulgué en vertu de la loi sur l'accès à l'information

As you know [redacted] is also providing extra funding for the spokes. We are working to come up with a scheme that meets your requirements and [redacted] requirements, as well as fairness and has a rationale that is defensible (it's not easy!). Would you be OK sharing with [redacted] what the overall proposal would be, therefore including what Canada would be contributing in detail by spoke? I'll be asking [redacted] the same thing. Without having the full picture it will be hard for either of you to understand.

Very best

Charles

Charles Gore | Executive Director | Medicines Patent Pool
Rue de Varembé 7, 1202 Geneva, Switzerland
Tel: +41 22 533 5050 | [redacted]
Mob: [redacted]
E-mail: [redacted]
@CharlieGore
Website | Newsletter

s.21(1)(b)

GAC - Released under the Access to Informations Act/
AMC - Divulgué en vertu de la loi sur l'accès à l'information

Geneva, 31 October 2022

Object: request for funding for the mRNA Technology Transfer Hub Programme

With this letter the Medicines Patent Pool (MPP) would like to request on behalf of the **mRNA Technology Transfer Hub Programme** a contribution of:

- [redacted] **(Funding Scenario A)** or
- [redacted] **(Funding Scenario B)**

from the **Department of Foreign Affairs, Trade and Development** (DFATD) of Canada, for the years 2023-2025 (the Programme is currently planning activities up to end 2026).

The MPP foundation is a non-profit international organisation based in Geneva (Switzerland), whose mission is to increase access to, and facilitate the development of, life-saving medicines for LMICs. We do this through an innovative approach to voluntary licensing and patent pooling. We work with a range of partners — civil society, international organisations, industry, patient groups and governments — to prioritise and license novel and existing medicines and health technologies for people in these countries (more information available at https://medicinespatentpool.org/).

The mRNA Technology Transfer Hub Programme, convened by the World Health Organization (WHO) and its partner MPP, aims to contribute to enabling equitable access to mRNA vaccines, by increasing the distribution of sustainable manufacturing capacity across countries, enhancing regional and inter-regional collaborations, and developing and empowering local workforce through tailored and inclusive trainings and expert support.

The mRNA Technology Transfer Hub Programme is expected to contribute to the overall improvement of health and health security in LMICs including future pandemic readiness.

The mRNA Technology Transfer Hub Programme operates to fulfill two complementary objectives:

- Establish and/or enhance sustainable mRNA vaccine manufacturing capacity;
- Develop skilled human capital in the regions where mRNA vaccine manufacturing capacity is established and/or enhanced.

To achieve these objectives, WHO and MPP have:

1) Initiated a collaboration with the South African mRNA Hub consortium to establish a hub/spoke model where:
    - a Hub (Afrigen) is developing an mRNA-based vaccine technology (first-generation);
    - the technology is initially transferred to the first technology recipient -spoke - (Biovac) that will also scale-up and validate the process received from Afrigen to the intended commercial scale;
    - research to develop an improved second-generation mRNA technology and a pipeline of novel mRNA vaccine candidates for SARS-CoV2 and other diseases is

s.15(1) - International

s.21(1)(b)

GAC - Released under the Access to Informations Act/
AMC - Divulgué en vertu de la loi sur l'accès à l'information

carried out through a collaboration established by SAMRC with other institutions.

2) Identified 14 additional recipient manufacturing facilities (spokes) all around the world that have expressed their interest in the Programme, will receive the technology developed by Afrigen and industrialized by Biovac and will eventually seek for local regulatory approval and establish their own vaccine production.

Currently, the budget to cover activities addressed at point 1 above has been estimated being ~$117 million, of which ~$70 million has been secured (received or expected). Significant sums have been spent already, particularly on equipment and reagents for the first-generation mRNA-vaccine technology development.

In reference to point 2 above, the spokes were initially told they would need to be self-supporting with no budget allocation from the mRNA technology Transfer Hub Programme. However, since the selection of the spokes, it has become apparent that the majority of them will require both financial and technical support to complete the objectives in point 2 above. The budget required for these activities and the detailed workplans are under definition as we assess their needs.

To cover the funding gap indicated above linked to the activities carried out by the South African Consortium (point 1 above) and to contribute to the spokes support (point 2 above), we would like to request from DFATD a contribution whose breakdown might look like one of the two following scenarios:

FUNDING SCENARIO A

A contribution of [redacted] for the years 2023-2025 to be used to support the activities of the Programme in the following way:

- [redacted] to cover expenses incurred by the South African mRNA Hub consortium to:
    - establish a technology transfer hub (technology donor) for mRNA COVID-19 vaccines development at Afrigen in South Africa;
    - transfer the technology (first-generation) developed by the hub to the first spoke (technology recipient), Biovac, that will also scale-up and validate the process and the analytical methods received from Afrigen;
    - develop an improved second-generation mRNA technology and a pipeline of novel mRNA vaccine candidates for SARS-CoV2 and other diseases through a collaboration established by SAMRC with other institutions.

- [redacted] to provide technical and financial support to the 14 other technology recipients (spokes) selected to date by the WHO with a focus on four of them (Senegal, Nigeria, Kenya, and Bangladesh) to take into account the priority areas of the Canadian development aid. This support, based on requests expressed by the entities, includes:
    - Bio-manufacturing training (or similar) to empower local workforce and ensure staff readiness to receive the technology transfer [redacted]
    - Procurement of critical equipment for the manufacturing of the mRNA vaccines [redacted]

GAC - Released under the Access to Informations Act/
AMC - Divulgué en vertu de la loi sur l'accès à l'information

s.15(1) - International

s.21(1)(b)

medicines patent pool

FUNDING SCENARIO B

A contribution of [redacted] for the years 2023-2025 would be used to support the activities of the Programme in the following way:

- [redacted] to cover expenses incurred by the South African mRNA Hub consortium to:
  - establish a technology transfer hub (technology donor) for mRNA COVID-19 vaccines development at Afrigen in South Africa;
  - transfer the technology (first-generation) developed by the hub to the first spoke (technology recipient), Biovac, that will also scale-up and validate the process and the analytical methods received from Afrigen;
  - develop an improved second-generation mRNA technology and a pipeline of novel mRNA vaccine candidates for SARS-CoV2 and other diseases through a collaboration established by SAMRC with other institutions.

- [redacted] to provide technical and financial support to the 14 other technology recipients (spokes) selected to date by the WHO with a focus on four of them (Senegal, Nigeria, Kenya, and Bangladesh) to take into account the priority areas of the Canadian development aid. This support, based on requests expressed by the entities, includes:
  - Bio-manufacturing training (or similar) to empower local workforce and ensure staff readiness to receive the technology transfer ([redacted];
  - Procurement of critical equipment for the manufacturing of the mRNA vaccines [redacted].

You will find below a provisional funding break-down of the **Funding Scenario A -** [redacted] **(Annex 1)** and of the **Funding Scenario B –** [redacted] **(Annex 2).**

As indicated above, while the South Africa mRNA Hub Consortium is developing the first-generation mRNA-vaccine technology and has already started activities to develop an improved second-generation mRNA technology and identified disease targets relevant to the low- and middle-income countries other than SARS-CoV2, the Programme is in the process of quantifying the readiness status to receive the mRNA technology and the level of support required by each spoke. For this reason, the provisional allocation of resources presented here is subject to change with the project progression and the advancements in the technology transfer to the spokes.

Should one of these two funding scenarios and corresponding breakdown be approved by DFATD, MPP will: diligently keep Canada (DFATD) informed of relevant changes of the needs and funding allocations (in any case, MPP will ensure that the priority given to the four spokes identified by Canada will be maintained); formally acknowledge the Canadian contribution and ensure due visibility is given on the MPP's (above-mentioned) actions on which Canada has provided funding.

Intellectual Property (IP) considerations have been at the forefront of this Programme from the start (and is one of the reasons WHO insisted on MPP's involvement as co-convener). The situation is both complicated and unclear: there is much overlapping IP and there are a number

GAC - Released under the Access to Informations Act/
AMC - Divulgué en vertu de la loi sur l'accès à l'information

of cases before the courts to establish who actually owns the IP to a number of key discoveries. From the beginning MPP's approach has been to map the patent landscape in LMICs and, if there are patents that could be constraining, to apply for licences. If that fails, then we look to invent around the patents. This is very much in line with how commercial companies address this subject. Finally, of course, countries have rights under the TRIPS agreement to take unilateral action to prevent patents blocking needed access, rights that have been stressed most recently at the World Trade Organization. It is worth being clear that MPP has no intention – and has publicly stated this – of infringing any patents, not least because MPP's key partners for licensing are pharmaceutical companies.

In the case of the hub Programme, MPP has significant dedicated funding for IP work and has carried out a thorough examination of existing patents as they relate to mRNA vaccines and is embarking on a project to map the IP associated with the lipids used in mRNA vaccine manufacture. Since this is an area of significant innovation, new patents are being continuously filed and MPP is tracking these on an ongoing basis. There are in fact very few patents of concern in LMICs. However, where they exist, MPP began by discussing licensing with BioNTech and Moderna, but without success. Therefore, Afrigen, with support from WHO and the world-renowned experts that comprise MPP's mRNA Scientific Advisory Committee, is looking at inventing around certain patents, primarily by using new processes more suitable to production in LMICs. In the rare cases where this is impossible and patents exist, it will be up to individual governments to decide whether to take advantage of the tools at their disposal. As an example, the South African government has convened a cross-department group to consider a particular Moderna patent that has been accepted in South Africa without any examination and is not granted anywhere else in the world.

We thank you in advance for the support offered for the activities carried out by MPP, specifically the mRNA Technology Transfer Hub Programme. Canada's contribution would make an enormous difference and could significantly contribute to closing the funding gap.

We would like to express a special thanks to the Government of Canada and, in particular to the individuals involved in the support to the hub Programme. It is a joy to work with you.

GAC - Released under the Access to Informations Act/
AMC - Divulgué en vertu de la loi sur l'accès à l'information

s.15(1) - International

s.21(1)(b)

medicines patent pool

ANNEX1:
FUNDING SCENARIO A breakdown and activities overview

The contribution of [redacted] would enable MPP to fund South African mRNA Hub consortium activities and spokes as depicted in **Table 1** below.

Differential amounts have been allocated to activities and countries considering preferences expressed by the DFATD and other available funds.

Table 1. Proposed funds allocation by country/spoke (lines) and activity (columns). Values are expressed in million CAD.

[redacted]

NOTE: for the purpose of the funding breakdown presented below, whenever budgeting was made available in USD, the exchange rate of 1USD=1.298CAD has been applied.

Description of the activities:

1. **SA consortium: 1st generation technology development, scale-up and validation and R&D on 2nd generation technology – [redacted]**

   To partially cover the expenses of the South African mRNA Hub consortium to:
   - establish a technology transfer hub (technology donor) for mRNA COVID-19 vaccines development at Afrigen in South Africa;
   - transfer the technology (first-generation) developed by the hub to the first spoke (technology recipient), Biovac, that will also scale-up and validate the process and the analytical methods received from Afrigen;
   - develop an improved second-generation mRNA technology and a pipeline of novel mRNA vaccine candidates for SARS-CoV2 and other diseases through a collaboration established by SAMRC with other institutions.

2. **Bio-manufacturing training – [redacted]**

   The WHO Bio-manufacturing training hub Programme, based on quotations received from different training providers, has estimated an average cost of 10.000 USD/person for a 2-week GxP biomanufacturing training course (virtual or in-person).

GAC - Released under the Access to Informations Act/
AMC - Divulgué en vertu de la loi sur l'accès à l'information

WHO, based on a high-level assessment of the spokes, has defined that, among the DFATD preferred countries, Bangladesh has less training needs with respect to the other countries due to their overall level of experience in vaccine manufacturing. Hence, training for 10 persons ([redacted] has been budgeted for Bangladesh and training for 20 persons ([redacted] has been budgeted for Nigeria and Kenya. Partial training costs have been allocated for Senegal ([redacted] as training will be covered also by other funding sources.
Based on need, additional training costs for the DFATD preferred countries and for the other countries might be covered by [redacted]) in the "All spokes" budget line and/or by other funding sources.

In addition, the Programme is negotiating the possibility for placements in Biotech companies (~60K USD/person). Costs for placements (still not allocated to countries) is also included in the [redacted] to "All spokes".

NOTE: Building capability through training is a critical factor towards the success of this project, therefore the training needs will be continuously monitored throughout the project as well as equitable and gender balanced access to training.

3. Equipment – [redacted]

A variable amount, up to [redacted] has been allocated to 4 countries (amounts based on preferences expressed by DFATD and other fundings available) to procure critical equipment for manufacturing at R&D (Technology Transfer Package1) and/or at commercial scale (Technology Transfer Packages 2/3) of mRNA-based vaccines.
Possible standard equipment to be procured (individual spokes needs assessment to be performed) could include:

- For Active Product Ingredient (API):
    - Bioreactors (for *E. coli* growth for pDNA production)
    - Chromatographers (mRNA purification)
    - Equipment for analytical testing of the pDNA and mRNA
- For nanoparticle formulations:
    - Homogenizer
    - Stirring tanks
    - Tangential Flow Filtration system for high volumes
    - Equipment for analytical testing of the finished product.

NOTE: as a reference,

a) [redacted]

b)

GAC - Released under the Access to Informations Act/
AMC - Divulgué en vertu de la loi sur l'accès à l'information

s.15(1) - International

s.21(1)(b)

medicines
patent
pool

ANNEX2:
FUNDING SCENARIO B breakdown and activities overview

The contribution of [redacted] would enable MPP to fund South African mRNA Hub consortium activities and spokes as depicted in **Table 2** below.

Differential amounts have been allocated to activities and countries considering preferences expressed by the DFATD and other available funds.

Table 2. Proposed funds allocation by country/spoke (lines) and activity (columns). Values are expressed in million CAD.

[redacted]

Description of the activities:

1. **SA consortium: 1st generation technology development, scale-up and validation and R&D on 2nd generation technology –** [redacted]

   To partially cover the expenses of the South African mRNA Hub consortium to:
   - establish a technology transfer hub (technology donor) for mRNA COVID-19 vaccines development at Afrigen in South Africa;
   - transfer the technology (first-generation) developed by the hub to the first spoke (technology recipient), Biovac, that will also scale-up and validate the process and the analytical methods received from Afrigen;
   - develop an improved second-generation mRNA technology and a pipeline of novel mRNA vaccine candidates for SARS-CoV2 and other diseases through a collaboration established by SAMRC with other institutions.

2. **Bio-manufacturing training –** [redacted]

   The WHO Bio-manufacturing training hub Programme, based on quotations received from different training providers, has estimated an average cost of 10.000 USD/person for a 2-week GxP biomanufacturing training course (virtual or in-person).

   WHO, based on a high-level assessment of the spokes, has defined that, among the DFATD preferred countries, Bangladesh has less training needs with respect to the other countries due to their overall level of experience in vaccine manufacturing. Hence, training for 10 persons [redacted] has been budgeted for Bangladesh and

GAC - Released under the Access to Informations Act/
AMC - Divulgué en vertu de la loi sur l'accès à l'information

s.15(1) - International

s.21(1)(b) s.20(1)(c)

training for 20 persons [redacted] has been budgeted for Nigeria and Kenya. Partial training costs have been allocated for Senegal ([redacted] as training will be covered also by other funding sources.
Based on need, additional training costs for the DFATD preferred countries and for the other countries might be covered by [redacted] in the "All spokes" budget line and/or by other funding sources.

In addition, the Programme is negotiating the possibility for placements in Biotech companies (~60K USD/person). Costs for placements (still not allocated to countries) is also included in the [redacted] to "All spokes".

NOTE: Building capability through training is a critical factor towards the success of this project, therefore the training needs will be continuously monitored throughout the project as well as equitable and gender balanced access to training.

3. Equipment – [redacted]

A variable amount, up to [redacted] has been allocated to 4 countries (amounts based on preferences expressed by DFATD and other fundings available) to procure critical equipment for manufacturing at R&D (Technology Transfer Package1) and/or at commercial scale (Technology Transfer Packages 2/3) of mRNA-based vaccines.
Possible standard equipment to be procured (individual spokes needs assessment to be performed) could include:

- For Active Product Ingredient (API):
  - Bioreactors (for *E. coli* growth for pDNA production)
  - Chromatographers (mRNA purification)
  - Equipment for analytical testing of the pDNA and mRNA
- For nanoparticle formulations:
  - Homogenizer
  - Stirring tanks
  - Tangential Flow Filtration system for high volumes
  - Equipment for analytical testing of the finished product.

NOTE: as a reference,

c) [redacted]

d)

Pages 112 to / à 113

are withheld pursuant to sections

sont retenues en vertu des articles

15(1) - International, 21(1)(b)

of the Access to Information Act

de la Loi sur l’accès à l’information

s.15(1) - International

s.21(1)(b)

GAC - Released under the Access to Informations Act/
AMC - Divulgué en vertu de la loi sur l'accès à l'information

MPP Funding request Scenario A_Draft budget_08Nov2022

Draft budget

Indicative figures only

1- Continued support of technology transfer activities South African Hub (Afrigen) and spoke (Biovac)	
• GMP (R&D) COVID-19 mRNA vaccine manufacturing process and analytical methods development<br>• mRNA vaccine batch production and testing for a Phase I/II clinical trial	
• GMP COVID-19 mRNA vaccine manufacturing process scale-up and process and analytical methods validation<br>• mRNA vaccine batches production and testing for a Phase III clinical trial	
• Develop a second-generation mRNA vaccine technology and establish a pipeline of vaccine candidates for Low- Middle-Income Countries	
2- Additional support for technology transfer activities across the spoke network	
• Bio-manufacturing training (or similar) to empower local workforce and ensure staff readiness to receive the technology transfer	
• Procurement of critical equipment for the manufacturing of the mRNA vaccines	
Total Direct Costs	
Indirect Cost (overhead)	
Total estimated budget	

GAC - Released under the Access to Informations Act/
AMC - Divulgué en vertu de la loi sur l'accès à l'information

s.15(1) - International

s.21(1)(b)

MPP Funding request Scenario B_Draft budget_08Nov2022

Draft budget

Indicative figures only

1- Continued support of technology transfer activities South African Hub (Afrigen) and spoke (Biovac)	
• GMP (R&D) COVID-19 mRNA vaccine manufacturing process and analytical methods development<br>• mRNA vaccine batch production and testing for a Phase I/II clinical trial	
• GMP COVID-19 mRNA vaccine manufacturing process scale-up and process and analytical methods validation<br>• mRNA vaccine batches production and testing for a Phase III clinical trial	
• Develop a second-generation mRNA vaccine technology and establish a pipeline of vaccine candidates for Low- Middle-Income Countries	
2- Additional support for technology transfer activities across the spoke network	
• Bio-manufacturing training (or similar) to empower local workforce and ensure staff readiness to receive the technology transfer	
• Procurement of critical equipment for the manufacturing of the mRNA vaccines	
Total Direct Costs	
Indirect Cost (overhead)	
Total estimated budget	

GAC - Released under the Access to Informations Act/
AMC - Divulgué en vertu de la loi sur l'accès à l'information

s.15(1) - International

s.21(1)(b)

Martin, Christine -MNV [She,Her | Elle]

From: Prakash, Vivek -DHAKA -DA
Sent: December 3, 2022 10:41 PM
To: Martin, Christine -MNV [She,Her | Elle]; Nickerson, Elaine -OAK
Cc: Goodings, Joe -DHAKA -DA; Bellows, Savannah -OAK [She,Her | Elle]
Subject: RE: CanGIVE update / Mise à jour IMEVCan

Thanks, Christine.

From: Martin, Christine -MNV [She,Her | Elle] <Christine.Martin@international.gc.ca>
Sent: December 2, 2022 7:11 PM
To: Prakash, Vivek -DHAKA -DA <Vivek.Prakash@international.gc.ca>; Nickerson, Elaine -OAK <ELAINE.NICKERSON@INTERNATIONAL.gc.ca>
Cc: Goodings, Joe -DHAKA -DA <Joe.Goodings@international.gc.ca>; Bellows, Savannah -OAK [She,Her | Elle] <Savannah.Bellows@international.gc.ca>
Subject: RE: CanGIVE update / Mise à jour IMEVCan

Dear all,

Thank you for your patience. Here is the information I received on the selection process:

In November 2021, the WHO launched a call for expressions of interest from potential recipient manufacturing facilities. Facilities were selected through a rigorous process conducted by the Product Development for Vaccines Advisory Committee, an independent standing WHO committee of experts. The committee developed specific criteria for the selection of recipient facilities, conducted independent assessments, and provided recommendations of potential manufacturers in accordance with the criteria.

Please let me know if you have further questions. The MPP proposal does include a breakdown of the funding distribution to the Bangladesh spoke but if anything else is missing, do not hesitate to reach out.

Kindest,
Christine

From: Martin, Christine -MNV [She,Her | Elle]
Sent: November 29, 2022 9:24 AM
To: Prakash, Vivek -DHAKA -DA <Vivek.Prakash@international.gc.ca>; Nickerson, Elaine -OAK <ELAINE.NICKERSON@INTERNATIONAL.gc.ca>
Cc: Goodings, Joe -DHAKA -DA <Joe.Goodings@international.gc.ca>; Bellows, Savannah -OAK [She,Her | Elle] <Savannah.Bellows@international.gc.ca>
Subject: RE: CanGIVE update / Mise à jour IMEVCan

Hi Vivek,

Absolutely. I will return with some lines on how MPP selects spokes and how the funding was allocated. My understanding is that we presented the list of CanGIVE countries to MPP and they prioritized Canadian funding being allocated to spokes and the Hub in CanGIVE countries [redacted] I have asked the PTL for this information and will circle back once I have more to share.

GAC - Released under the Access to Informations Act/
AMC - Divulgué en vertu de la loi sur l'accès à l'information

In the meantime, please let me know if there is anything else I can provide.

Kindest,
Christine

From: Prakash, Vivek -DHAKA -DA <Vivek.Prakash@international.gc.ca>
Sent: November 27, 2022 1:18 AM
To: Nickerson, Elaine -OAK <ELAINE.NICKERSON@INTERNATIONAL.gc.ca>; Martin, Christine -MNV [She,Her | Elle] <Christine.Martin@international.gc.ca>
Cc: Schulz, Sara -MNV [She,Her | Elle] <Sara.Schulz@international.gc.ca>; Goodings, Joe -DHAKA -DA <Joe.Goodings@international.gc.ca>; Bellows, Savannah -OAK [She,Her | Elle] <Savannah.Bellows@international.gc.ca>
Subject: RE: CanGIVE update / Mise à jour IMEVCan

Thanks for this. It might also be helpful for us to have any off-the-shelf lines on how we made those choices. You may recall that Bangladesh was keen to manufacture mRNA vaccines domestically and does have in place most of the physical infrastructure. (More likely Joe might receive that particular question, given his bilateral program lead.)

Vivek Prakash
Head of Cooperation (Rohingya refugee response) | Chef de Coopération (réponse aux refugiés rohingyas)
High Commission of Canada, Dhaka, Bangladesh | Haut-commissariat du Canada, Dacca, Bangladesh
United Nations Road, Baridhara, Dhaka - 1212
Tel: +88 02 55668444 ext. 319-3460 | Mitnet: 319-3460
Mobile: +880 16 8810 3755

Vivek.Prakash@international.gc.ca

Government of Canada | Gouvernement du Canada

From: Nickerson, Elaine -OAK <ELAINE.NICKERSON@INTERNATIONAL.gc.ca>
Sent: November 23, 2022 5:41 AM
To: Martin, Christine -MNV [She,Her | Elle] <Christine.Martin@international.gc.ca>
Cc: Schulz, Sara -MNV [She,Her | Elle] <Sara.Schulz@international.gc.ca>; Goodings, Joe -DHAKA -DA <Joe.Goodings@international.gc.ca>; Prakash, Vivek -DHAKA -DA <Vivek.Prakash@international.gc.ca>; Bellows, Savannah -OAK [She,Her | Elle] <Savannah.Bellows@international.gc.ca>
Subject: FW: CanGIVE update / Mise à jour IMEVCan

Hello, Christine,

Would you be able to send the approval document for the approvals mentioned below. We have two new heads of cooperation in Dhaka; one for the Bangladesh program and one for the Rohingya program, and I would like to ensure they are briefed on the Can-Give initiatives in Bangladesh (they do have the draft action plan).

Thank you in advance.

Elaine

GAC - Released under the Access to Informations Act/
AMC - Divulgué en vertu de la loi sur l'accès à l'information

From: Schulz, Sara -MNV [She,Her | Elle] <Sara.Schulz@international.gc.ca>
Sent: November 9, 2022 1:17 PM
To: Morris, Phedra Moon -DHAKA -DA <PhedraMoon.Morris@international.gc.ca>; Yampolsky, Raya -DHAKA -GR <Raya.Yampolsky@international.gc.ca>; Sebhatu, Joseph -DHAKA -DA <Joseph.Sebhatu@international.gc.ca>; Nickerson, Elaine -OAK <ELAINE.NICKERSON@INTERNATIONAL.gc.ca>; Labelle, Chantal -MINE <Chantal.Labelle@international.gc.ca>; Munoz, Diana -BGOTA -DA [She/Her | Elle] <Diana.Munoz@international.gc.ca>; Dubé, Marie-Christine -NLA <Marie-Christine.Dube@international.gc.ca>; Timma-Toupé, Joëlle -NLA <Joelle.Timma-Toupe@international.gc.ca>; Bedoya, Ana María -BGOTA -DA <AnaMaria.Bedoya@international.gc.ca>; Monnard, Pierre-Yves -NDH <Pierre-Yves.Monnard@international.gc.ca>; Dorval, Omilty -PRNCE -DA <Omilty.Dorval@international.gc.ca>; Weber, David -NDE <David.Weber@international.gc.ca>; Dunkley Elliott, Emma -KNGTN -DA <Emma.DunkleyElliott@international.gc.ca>; Borgognoni, Anna -NDE [She,Her | Elle] <Anna.Borgognoni@international.gc.ca>; Myles, Jennifer -WWC <jennifer.myles@international.gc.ca>; Provost-Vallieres, Charlotte -WWC <charlotte.provostvallieres@international.gc.ca>; Hughes, Catherine -WWC <Catherine.Hughes@international.gc.ca>; Kane, Amadou -KNSHA -DA <Amadou.Kane@international.gc.ca>; Cosgrove, Erin -WWL <erin.cosgrove@international.gc.ca>; Robert, Roxanne -WWL <Roxanne.Robert@international.gc.ca>; Rousselle, Élodie -WWL [Elle | She,Her] <Elodie.Rousselle@international.gc.ca>; Gesnot, Nicole -ABDJN -DA [She,Her | Elle] <Nicole.Gesnot@international.gc.ca>; Flanagan, Shauna -ACCRA -DA [She,Her | Elle] <Shauna.Flanagan@international.gc.ca>; Loyer, Adam -ACCRA -DA <Adam.Loyer@international.gc.ca>; Trépanier, Erianne -WWG [She,Her | Elle] <Erianne.Trepanier@international.gc.ca>; Joshua, Rebecca -WWG <Rebecca.Joshua@international.gc.ca>; Byers, Meaghan -WEL [She,Her | Elle] <Meaghan.Byers@international.gc.ca>; Royal, Emily -WEL <Emily.Royal@international.gc.ca>; Hassanein, Ashraf -MPUTO -DA <Ashraf.Hassanein@international.gc.ca>; Myles, Jennifer -WWC <jennifer.myles@international.gc.ca>; Provost-Vallieres, Charlotte -WWC <charlotte.provostvallieres@international.gc.ca>; Lemieux, Alexia -WWC <Alexia.Lemieux@international.gc.ca>; Pistone, Renata -ABUJA -DA <Renata.Pistone@international.gc.ca>; Osubor, Martin - ABUJA -DA <Martin.Osubor@international.gc.ca>; Gagné, Karl -WWS <karl.gagne@international.gc.ca>; Landry, Claude -DAKAR -DA [She,Her | Elle] <Claude.Landry@international.gc.ca>; Pedneault, Sylvie -DAKAR -DA [She,Her | Elle] <Sylvie.Pedneault@international.gc.ca>; Steinhouse, Lara -WWS <Lara.Steinhouse@international.gc.ca>; Boutilier, Zoe -CFXL <Zoe.Boutilier@international.gc.ca>; Clark, Andrew -WEK [He,Him | Il] <Andrew.Clark@international.gc.ca>; Khawam, Carine -PRET -DA <Carine.Khawam@international.gc.ca>; Hindle, Laird -WER [He,Him | Il] <Laird.Hindle@international.gc.ca>; Oliver, Jessica -WEK <jessicac.oliver@international.gc.ca>; Legros, Guillaume -WEG <Guillaume.Legros@international.gc.ca>; Roberts, Tommie Eugène -WEG <TommieEugene.Roberts@international.gc.ca>; Brunet, Stephanie -DSLAM -DA <Stephanie.Brunet@international.gc.ca>; McDowell, Charlotte -PRMOAS -GR <Charlotte.McDowell@international.gc.ca>; Nickerson, Elaine -OAK <ELAINE.NICKERSON@INTERNATIONAL.gc.ca>; Nolet, Alain -WFO <alain.nolet@international.gc.ca>; Fitchett, Janice -POR <Janice.Fitchett@international.gc.ca>; Le Courtois, Sandra -OAZ [She,Her | Elle] <Sandra.LeCourtois@international.gc.ca>; Harvey, Marie-Claude -NDH <Marie-Claude.Harvey@international.gc.ca>; Guimond, Alexandre -NLG <Alexandre.Guimond@international.gc.ca>; Henderson, Jenifer -NDS [She,Her | Elle] <Jenifer.Henderson@international.gc.ca>; Maitland, Brett -NDS [He,Him | Il] <Brett.Maitland@international.gc.ca>; Seekings, David -NDS <David.Seekings@international.gc.ca>; Duchesneau-Custeau, Alyssa -NDS [She,Her | Elle] <Alyssa.Duchesneau-Custeau@international.gc.ca>; Barnes, Veronique -NDS [She,Her | Elle] <Veronique.Barnes@international.gc.ca>; Pineda, Cristina -NDS [She,Her | Elle] <Cristina.Pineda@international.gc.ca>; Napier, Vienna -WFO <Vienna.Napier@international.gc.ca>; Allman, Annika -WFO <Annika.Allman@international.gc.ca>; Bernadotte, Maeva -NLG <Maeva.Bernadotte@international.gc.ca>
Cc: Tarr, Michael -MNV [He,Him | Il] <Michael.Tarr@international.gc.ca>; Martin, Christine -MNV [She,Her | Elle] <Christine.Martin@international.gc.ca>; Hisko, Mellissa -MNV [She,Her | Elle] <mellissa.hisko@international.gc.ca>
Subject: CanGIVE update / Mise à jour IMEVCan

****le français suit****

Colleagues,

GAC - Released under the Access to Informations Act/
AMC - Divulgué en vertu de la loi sur l'accès à l'information

I'm pleased to share some new updates regarding Canada's Global Initiative for Vaccine Equity (CanGIVE).

As discussed during our August meeting, we encourage you to engage locally with partners to discuss the relevant CanGIVE projects happening in your countries of accreditation. It's been great to hear from many of you about various opportunities to meet with partners in the past few months. We remain available for any assistance around partner engagements or CanGIVE updates. On that note, I would like to introduce Christine Martin, who has taken over as the CanGIVE lead within the COVID-19 Team and will be your best point of contact moving forward. To assist with any local engagements, please find attached the CanGIVE Issue Brief, as well as the latest version of the placemat. The documents reflect the shifting vaccination landscape and integration of COVID-19 vaccination within routine immunization campaigns and broader health system strengthening efforts.

Since our last update, I am happy to report that all CanGIVE projects have received Ministerial approval. PTLs are finalizing grant arrangements with the different CanGIVE partners. UNICEF has already received funding to launch its COVID-19 vaccine delivery project. Other projects should launch by December or early January. We will inform everyone as arrangements are signed and funds are disbursed.

I am also thrilled to share that we are awaiting approval of an additional $117M funding allocation to CanGIVE from Canada's ACT-Accelerator commitment in Budget 2022. This new top-up funding will enable existing CanGIVE partners to expand activities related to COVID-19 vaccination and health systems strengthening, including enhancing routine immunization services. As we receive proposals from partners and have clarity on additional funding options for CanGIVE countries, we will connect with you to provide an update on the latest developments and solicit your input on the proposals. We anticipate proposed project increases will receive Ministerial approval over December.

As always, thank you for your collaboration to bring this initiative to fruition. Please do not hesitate to reach out to Christine or myself if you have questions.

Sincerely,
Sara

Sara Schulz
Deputy Director | Directrice adjointe
COVID-19 Global Health Response Task Force (MNV) | Groupe de travail de la réponse sanitaire mondiale de la COVID-19 (MNV)
Health and Nutrition Bureau (MND) | Direction générale de la Santé et de la Nutrition (MND)
sara.schulz@international.gc.ca
Tel. : 343-548-9938

***
Collègues,

Je suis heureuse de partager de nouvelles mises à jour concernant l'Initiative mondiale pour l'équité vaccinale du Canada (IMEVCan).

Comme discuté lors de notre réunion d'août, nous vous encourageons à engager les partenaires sur le terrain afin de discuter des projets de l'IMEVCan pertinents qui se déroulent dans vos pays d'accréditation. Il a été formidable d'entendre de plusieurs d'entre vous au cours des derniers mois concernant diverses opportunités pour engager ces partenaires. Nous restons disponibles pour toute assistance concernant les engagements des partenaires ou les mises à jour de l'IMEVCan. Sur cette note, j'aimerais vous présenter Christine Martin, qui a pris la tête de l'IMEVCan au sein de l'équipe COVID-19 et qui sera votre meilleur point de contact dorénavant. Pour vous aider dans vos engagements locaux, veuillez trouver ci-joint la note d'information de l'IMEVCan, ainsi que la dernière version du napperon. Les documents reflètent l'évolution du contexte de la vaccination et l'intégration de la vaccination contre le COVID-19 dans les campagnes de vaccination systématique, ainsi que les efforts plus larges de renforcement des systèmes de santé.

GAC - Released under the Access to Informations Act/
AMC - Divulgué en vertu de la loi sur l'accès à l'information

Depuis notre dernière mise à jour, je suis heureuse d'annoncer que tous les projets de l'IMEVCan ont reçu l'approbation ministérielle. Les chargés de projets finalisent les accords de subvention avec les différents partenaires de l'IMEVCan. L'UNICEF a déjà reçu des fonds pour lancer son projet de soutien pour la livraison de vaccins contre la COVID-19. Les autres projets devraient être lancés d'ici décembre ou début janvier. Nous vous informerons au fur et à mesure que les arrangements seront signés et que les fonds seront déboursés.

Je suis également ravie de partager que nous attendons l'approbation d'une allocation de financement supplémentaire de 117 millions de dollars à l'IMEVCan dans le cadre de l'engagement du Canada envers l'Accélérateur ACT dans le budget 2022. Ce nouveau financement complémentaire permettra au partenaires actuels de l'IMEVCan d'étendre leurs activités liées à la vaccination contre la COVID-19 et au renforcement des systèmes de santé, y compris l'amélioration des services de vaccination systématique. Au fur et à mesure que nous recevrons les propositions des partenaires et que nous aurons plus de détails sur les options de financement supplémentaires pour les pays de l'IMEVCan, nous vous contacterons pour partager une mise à jour sur les derniers développements et solliciter vos commentaires sur les propositions. Nous prévoyons que les augmentations de projet proposées recevront l'approbation ministérielle en décembre.

Comme toujours, merci de votre collaboration pour mener à bien cette initiative. N'hésitez pas à contacter Christine ou moi-même si vous avez des questions.

Sincèrement,
Sara

Sara Schulz
Deputy Director | Directrice adjointe
COVID-19 Global Health Response Task Force (MNV) | Groupe de travail de la réponse sanitaire mondiale de la COVID-19 (MNV)
Health and Nutrition Bureau (MND) | Direction générale de la Santé et de la Nutrition (MND)
sara.schulz@international.gc.ca
Tel. : 343-548-9938

GAC - Released under the Access to Informations Act/
AMC - Divulgué en vertu de la loi sur l'accès à l'information

s.19(1)

Tarr, Michael -MNV [He,Him | Il]

From: Tarr, Michael -MNV [He,Him | Il]
Sent: December 20, 2022 9:07 AM
To: 'Monica Moschioni'
Cc: Ike James; Gelise McCullough; Charles Gore
Subject: RE: Update
Attachments: Medicines Patent Pool PO 7438737 Grant Agreement SIGNED.PDF; Draft Annex A_MPP_November 2022_20 Dec.docx

Hi Monica,

Thanks kindly for the thorough and timely review and suggested edits. We are good with all changes – attached in clean document.

To answer your questions:

1. Yes – these will be removed. We will move forward with the high-level budget in this Annex.
2. Absolutely – within the program scope and outcomes there is budget flexibility across the activities. I have included a line below the budget to indicate this – let me know if this works for you.
3. Correct – this will be outlined in the main agreement. Duration will be 24 months (January 2023 – December 2024).

I will return shortly (today or tomorrow) with the full agreement for your review and signature. In the meantime, as requested please find the previous agreement signed by both parties.

Best,
Mike

From: Monica Moschioni [redacted]
Sent: December 19, 2022 12:05 PM
To: Tarr, Michael -MNV [He,Him | Il] <Michael.Tarr@international.gc.ca>
Cc: Ike James [redacted] Gelise McCullough [redacted];
Charles Gore [redacted]
Subject: RE: Update

Hi Michael,
PFA the Annex A with our proposed modifications and a proposed breakdown of the 45M CAD.
As you will see the modifications are mainly reflecting the evolution of Programme activities.

There are few explanations included in the comments as well.

Few additional questions/comments:

1. Would it be possible to remove annex B (individual budgets of Afrigen, Biovac and SAMRC) from the contract? These have been already amended and we expect additional amendments to happen in the next future along with the progression/better definition of the activities.
2. How much flexibility will there be across the "budget breakdown" lines? The figures presented here have been derived taking into account the money already disbursed and foreseen disbursements based on the current budgets. Obviously, these proportional allocations might change along with budgets modifications (see point 1).

s.19(1)

GAC - Released under the Access to Informations Act/
AMC - Divulgué en vertu de la loi sur l'accès à l'information

3. The years covered by the amended contract will need to be clarified along with the time by when MPP has to disburse the money to the Partners. We are happy in this being specified only in the body of the contract by keeping here only the total number of years (5 years, assuming the years to be 2022-2026).

Finally, can you share please the initial contract signed by your side? We only have a version signed by Charles and the auditors requested to see a fully signed version.

Happy to discuss with you all the proposed modifications and the next steps over a call as needed. I will be available the whole week.

Have a nice day

Monica

From: Charles Gore <[redacted]
Sent: Tuesday, 13 December 2022 18:13
To: Michael.Tarr@international.gc.ca
Cc: Monica Moschioni [redacted]; Ike James <[redacted] Gelise McCullough <[redacted]
Subject: Re: Update

Yes we're shutting the week of the 26th (everyone needs a break, including me!)

Very best

Charles

Charles Gore | Executive Director | Medicines Patent Pool
Rue de Varembé 7, 1202 Geneva, Switzerland
Tel: +41 22 533 5050 | Direct Tel : [redacted]
Mob: +[redacted]
E-mail: [redacted]
@CharlieGore
Website | Newsletter

From: "Michael.Tarr@international.gc.ca" <Michael.Tarr@international.gc.ca>
Date: Tuesday, 13 December 2022 at 14:01
To: Charles Gore [redacted]
Cc: Monica Moschioni <[redacted], Ike James [redacted], Gelise McCullough <[redacted]
Subject: RE: Update

Dear Charles,

Next Monday is fine – understand the bad timing. We can aim to move things quickly next week.

Just for planning – is there a window over the holidays that MPP is offline? I will be off Dec 26-27 but otherwise here to move the agreement along.

GAC - Released under the Access to Informations Act/
AMC - Divulgué en vertu de la loi sur l'accès à l'information

Best,
Mike

From: Charles Gore [redacted]
Sent: December 13, 2022 7:44 AM
To: Tarr, Michael -MNV [He,Him | Il] <Michael.Tarr@international.gc.ca>
Cc: Monica Moschioni <[redacted]; Ike James <[redacted]>; Gelise McCullough <[redacted]
Subject: Re: Update

Dear Mike,

Thanks so much for this. Unfortunately [redacted] so I may not be able to get this back to you until Monday close of business. Would you be OK with that? It's just a very unfortunate week

Very best

Charles

Charles Gore | Executive Director | Medicines Patent Pool
Rue de Varembé 7, 1202 Geneva, Switzerland
Tel: +41 22 533 5050 | Direct Tel : [redacted]
Mob: [redacted]
E-mail: [redacted]
@CharlieGore
Website | Newsletter

From: "Michael.Tarr@international.gc.ca" <Michael.Tarr@international.gc.ca>
Date: Monday, 12 December 2022 at 18:36
To: Charles Gore <[redacted]
Cc: Monica Moschioni <[redacted]>, Ike James [redacted] Gelise McCullough <[redacted]
Subject: RE: Update

Dear Charles,

Please find attached for your review the draft Annex for the grant agreement.

We have retained the framework/language of the previous Annex and added new text (highlighted in yellow) to reflect the additional funding. This new Annex will effectively replace/supersede the original one, becoming the agreement for Canada's overall funding. In this light, the budget will need to reflect the full amount ($45M).

Would be very grateful if you could send back with any comments/changes and the completed budget table at your earliest convenience. We will then add this Annex to the grant agreement and send back for your signature.

A huge thanks to you and the team for all the work to get us to this point. Look forward to connecting to the project in the new year – would very much welcome the opportunity to combine a site visit with the funders forum in April.

Best,
Mike

GAC - Released under the Access to Informations Act/
AMC - Divulgué en vertu de la loi sur l'accès à l'information

s.19(1)

From: Charles Gore <[redacted]
Sent: December 9, 2022 6:15 AM
To: Tarr, Michael -MNV [He,Him | Il] <Michael.Tarr@international.gc.ca>
Cc: Monica Moschioni <[redacted]; Ike James <[redacted]; Gelise McCullough <[redacted]
Subject: Re: Update

Dear Mike

That's terrific news. Thank you so much!

We can turn things around very fast so should be fine from our end.

We are planning to hold the funders forum in April in Cape Town. Might that work for you for a site visit?

Very best

Charles

Charles Gore
Executive Director
Medicines Patent Pool
Rue de Varembé 7
1202 Genève
[redacted]

From: Michael.Tarr@international.gc.ca <Michael.Tarr@international.gc.ca>
Sent: Friday, December 9, 2022 7:45:02 AM
To: Charles Gore <[redacted]
Cc: Monica Moschioni <[redacted]; Ike James <[redacted] Gelise McCullough <[redacted]
Subject: Update

Dear Charles

Hope you are doing well.

To follow up on the G20 announcement - below is the news release highlighting a package of funding, including that to MPP and PAHO as part of Canada's overall investment in regional manufacturing.

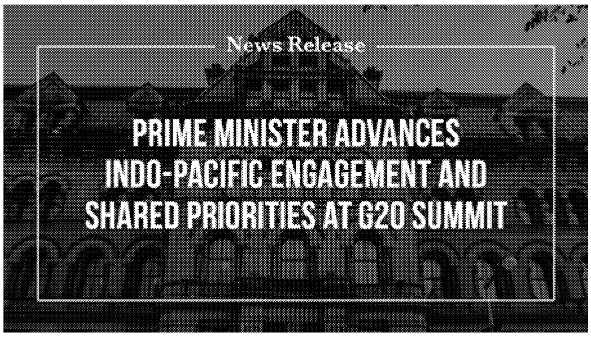

GAC - Released under the Access to Informations Act/
AMC - Divulgué en vertu de la loi sur l'accès à l'information

Prime Minister advances Indo-Pacific engagement and shared priorities at G20 Summit
pm.gc.ca

The amount announced by our PM was $15M, however there is good news to share on funding scenario B that you prepared for us. I am currently waiting to receive the draft grant agreement from our contracts team before sending to you for review.

We are looking to have the agreement signed by end of calendar year, which I understand is a short turnaround at a busy time. I am hoping to be able to send you on Monday.

Once this piece is finalized we can work together with Gelise on the comms front - on the website and other opportunities as well. We would also be very interested in a site visit at some point in the new year.

Best,
Mike

Sent from my iPhone

GAC - Released under the Access to Informations Act/
AMC - Divulgué en vertu de la loi sur l'accès à l'information

Grant Agreement

Amendment 1

Project no: P011103
Agreement no.: 7438737

The Grant Agreement made in duplicate on March 18, 2022

BETWEEN ***Her Majesty the Queen in right of Canada*** ("Her Majesty"), represented by the Minister for International Development, acting through the Department of Foreign Affairs, Trade and Development ("DFATD")

AND ***Medicines Patent Pool*** ("the Organization"), a legal entity having its head office/principal place of business at:

Rue de Varembé 7, fifth floor, 1202 Geneva, Switzerland

WHEREAS, DFATD provides a Grant to Medicines Patent Pool to support the Project entitled "**COVID-19 mRNA Technology Transfer and Manufacturing Hub**",

is hereby amended as follows:

Amendment 1: This amendment n°1 increases the financial limitation of the Agreement by thirty million Canadian dollars (CDN $ 30,000,000) for a new total value of forty-five million Canadian dollars (CDN $ 45,000,000); extends the duration of the Agreement by nine (9) months and provides for certain changes to the articles 2 and 7, the Annex A (Project Description) and Annex B (Estimated Budget) of the Grant Agreement.

THEREFORE, DFATD and the Organization agree on the following:

1. In Article 2, **Terms of Payment**, **DELETE** the sub-article 2.1 in its entirety and **REPLACE** with the following:

   "2.1. Upon signature of this Agreement by both parties, DFATD shall make a total payment of an amount of forty-five million Canadian Dollars (CDN $ 45,000,000) based on the following instalment schedule for the purpose of the Project described in Annex A under DFATD's fiscal years 2021-2022 / 2022-2023 (the "Grant"):

	Payment, no later than	Amount	DFATD Fiscal Year
1.	March 31, 2022	CDN $ 15,000,000	2021-2022
2.	March 31, 2023	CDN $ 30,000,000	2022-2023

s.19(1)

GAC - Released under the Access to Informations Act/
AMC - Divulgué en vertu de la loi sur l'accès à l'information

"

2. In Article 7 - **Duration**, **DELETE** the sub-article 7.1 in its entirety and **REPLACE** with the following:

   "7.1. This Agreement shall come into effect upon the date of the last signature and shall expire on December 31, 2024."

3. **Annex A, Project Description**, is **AMENDED** in its entirety and **REPLACED** with the attached revised **Annex A, Project Description**.

4. **Annex B – Estimated Budget** for the Project is **AMENDED** in its entirety and **REPLACED** with the attached revised **Annex B – Estimated Budget for the Project**.

5. All other terms of the Grant Agreement remain unchanged.

This Amendment will become effective on the date of the last signature.

Done in the English language in two (2) copies,

For the Medicines Patent Pool	For the Department of Foreign Affairs, Trade and Development
Signature [redacted]	Signature Tabah, Joshua B — Digitally signed by Tabah, Joshua B Date: 2022.12.22 08:54:26 -05'00'
Name Charles Gore	Name
Title Executive Director	Title
Date 22nd December 2022	Date

GAC - Released under the Access to Informations Act/
AMC - Divulgué en vertu de la loi sur l'accès à l'information

Annex A - PROJECT DESCRIPTION

1.1 Canada is providing an additional $30 million, for a total of CAD $45 million, to the Medicines Patent Pool (MPP) to support the mRNA Technology Transfer Hub Programme, sitting under the COVAX umbrella and convened by MPP and the World Health Organization (WHO).

1.2 Medicines Patent Pool, on behalf of the mRNA Technology Transfer Hub Programme, is responsible to sign agreements with the different partners participating in the Programme. Working through Medicines Patent Pool, this funding supports the Programme partners to: i) establish a technology transfer hub for mRNA vaccines in South Africa, ii) develop and validate an mRNA based technology platform suitable for vaccines manufacturing (using COVID-19 as proof of concept) at the South African Hub; iii) facilitate technology transfer to recipient manufacturing facility/facilities selected by WHO; iv) support the recipient facility/facilities in establishing mRNA vaccine production capabilities, and v) conduct research activities aiming at developing a second-generation mRNA vaccine technology and a pipeline of vaccine candidates relevant to Low- Middle-Income Countries (LMICs). This project is part of Canada's commitment to the ACT-Accelerator.

1.3 The full scope of the five-year initiative (total budget of roughly CAD$158 million) is to establish voluntary technology transfer to several recipient facilities in Africa, Latin America and Asia. Canada's funding will support: the establishment of the technology transfer hub itself (Afrigen Biologics); the development of a COVID-19 mRNA vaccine manufacturing platform; the production and testing of the COVID-19 vaccine in a Phase I/II clinical trial; the transfer of the mRNA technology platform to the first recipient facility (Biovac); the scale up and validation of the technology platform at Biovac; the production of material for a Phase III clinical trial (Biovac); the transfer of the technology platform to a network of recipient facilities; research activities to develop a second-generation mRNA vaccine technology (South African Medical Research Council). Exploration into the application of the developed mRNA technology beyond COVID-19 for other infectious diseases relevant to LMICs, including HIV and tuberculosis, will be conducted in partnership with the South African Medical Research Council. Technical and financial support will also be delivered, based on identified needs, to technology recipients, including the priority countries of Senegal, Nigeria, Kenya and Bangladesh.

2. EXPECTED RESULTS

2.1 The overall aim of this project is to contribute to equitable access to mRNA vaccines by increasing the distribution of sustainable manufacturing capacity across LMICs, enhancing regional and inter-regional collaborations, as well as developing and empowering a local workforce. This goal will be achieved by supporting the development of the technology platform, the multilateral transfer of the technology platform to technology recipients and by providing corresponding support to technology recipient "spoke" facilities to enable technology transfer.

GAC - Released under the Access to Informations Act/
AMC - Divulgué en vertu de la loi sur l'accès à l'information

2.2 The project will support increased regional availability of mRNA vaccines for COVID-19 and explore the broader application of the developed mRNA-based technology for vaccines manufacturing towards additional infectious diseases relevant to LMICs. The goal is to achieve ultimate production at a first facility capable of reaching 50-100[1] million doses per year for the southern African region.

2.3 Intermediate Outcomes

- Established or enhanced sustainable mRNA vaccine manufacturing capacity in regions with limited or no vaccine manufacturing capacity or where vaccine manufacturing capacity exists but with different technologies.
- Increased skilled human capital, particularly amongst women, in biomanufacturing, technology transfer and regulatory capabilities.

2.4 Reporting will be provided annually and produced in coordination with the World Health Organization. Regular updates will also be provided via the mRNA Technology Transfer Hub Programme Funders Forum quarterly meetings and monthly updates. Annual reports will include:

- Summary of activities conducted at the South African hub consortium and spokes facilities;
- Key progress indicators;
- Gender-disaggregated data for human capital employed on Programme activities at the hub and participating to “spokes” training;
- Risks and mitigation with respect to the development and implementation of the project;
- Financial statements.

3. PROJECT ACTIVITIES

3.1 Canada’s funding will primarily support the establishment of the mRNA Technology platform, through activities conducted by the South African Consortium such as:

- Preparation and start-up of the Hub facilities;
- Training of staff and technical assistance at the Hub facility;
- Development of the mRNA vaccine technology (process and analytical methods) by using COVID-19 as Proof of Concept at the Hub;

[1] To be further refined as additional facilities in the mRNA network begin production, demand for COVID-19 vaccines becomes clearer and mRNA vaccine development for additional diseases is tested.

GAC - Released under the Access to Informations Act/
AMC - Divulgué en vertu de la loi sur l'accès à l'information

- Continued analysis and review of intellectual property landscape and regulatory processes;
- Research and development activities, including preclinical and clinical studies;
- Design of the technology transfer programme;
- Maintenance of the Hub facilities;
- Scale-up and validation of the mRNA vaccine manufacturing platform at the first spoke (Biovac);
- Research to develop a second-generation improved mRNA technology and to apply the mRNA technology to different target diseases relevant to LMICs; and
- Other miscellaneous activities, as required.

3.2 Canada's funding will also support identified needs of recipient manufacturing facilities, including in the priority countries of Senegal, Nigeria, Kenya and Bangladesh, with activities including, but not limited to:

- Training of staff on good quality practice biomanufacturing, specifically on the mRNA technology;
- Preparation of facilities, including procurement of critical equipment, to receive mRNA technology;
- Transfer of the technology;
- Other miscellaneous activities, as required.

GAC - Released under the Access to Informations Act/
AMC - Divulgué en vertu de la loi sur l'accès à l'information

ANNEX B – Estimated Budget for the Project

**1- Continued support for technology transfer activities to the South African Hub (Afrigen) and spoke (Biovac)**	
• Preparation and start up of the hub facility<br>• GMP (R&D) COVID-19 mRNA vaccine manufacturing process and analytical methods development<br>• mRNA vaccine batch production and testing for a Phase I/II clinical trial<br>• Pre-clinical and clinical Phase I/II studies	
• GMP COVID-19 mRNA vaccine manufacturing process scale-up and process and analytical methods validation<br>• mRNA vaccine production and testing for a Phase III clinical trial	
• Research activities to develop a second-generation mRNA vaccine technology and establish a pipeline of vaccine candidates for Low- Middle-Income Countries	
**2- Additional support for technology transfer activities across the spoke network***	
• Bio-manufacturing training (or similar) to empower local workforce and ensure staff readiness to receive the technology transfer	
• Procurement of critical equipment for the manufacturing of the mRNA vaccines	
**Total Direct Costs**	**CAD$ 43.15 M**
**Indirect Costs (overhead)**	**CAD$ 1.85M**
**Total estimated budget**	**CAD $ 45M**

*Funding will be provided across the network of recipient facilities, including the priority countries of Nigeria, Senegal, Kenya, and Bangladesh, based on needs identified by Medicines Patent Pool.

The program will respond to partner-driven needs to achieve increased manufacturing capacity of mRNA vaccines, maintaining the required budget flexibility to ensure all critical activities can be completed.

GAC - Released under the Access to Informations Act/
AMC - Divulgué en vertu de la loi sur l'accès à l'information

ANNEX A: COVID-19 Technology Transfer and Manufacturing Hub Implementation Plan

1. PROJECT DESCRIPTION

1.1 Canada is providing an additional $30 million, for a total of CAD $45 million, to the Medicines Patent Pool (MPP) to support the mRNA Technology Transfer Hub Programme, sitting under the COVAX umbrella and convened by MPP and the World Health Organization (WHO).

1.2 Medicines Patent Pool, on behalf of the mRNA technology Transfer Hub Programme is responsible to sign agreements with the different partners participating the Programme. Working through Medicines Patent Pool, this funding supports the Programme partners to: i) establish a technology transfer hub for mRNA vaccines in South Africa, ii) develop and validate an mRNA based technology platform suitable for vaccines manufacturing (using COVID-19 as proof of concept) at the South African Hub; iii) facilitate technology transfer to recipient manufacturing facility/facilities selected by WHO; iv) support the recipient facility/facilities in establishing mRNA vaccine production capabilities, and v) conduct research activities aiming at developing a second-generation mRNA vaccine technology and a pipeline of vaccine candidates relevant to Low- Middle-Income Countries (LMICs). This project is part of Canada's commitment to the ACT-Accelerator.

1.3 The full scope of the five-year initiative (total budget of roughly CAD$158 million) is to establish voluntary technology transfer to several recipient facilities in Africa, Latin America and Asia. Canada's funding will support: the establishment of the technology transfer hub itself (Afrigen Biologics); the development of a COVID-19 mRNA vaccine manufacturing platform; the production and testing of the COVID-19 vaccine in a Phase I/II clinical trial; the transfer of the mRNA technology platform to the first recipient facility (Biovac); the scale up and validation of the technology platform at Biovac; the production of material for a Phase III clinical trial (Biovac); the transfer of the technology platform to a network of recipient facilities; research activities to develop a second-generation mRNA vaccine technology (South African Medical Research Council). Exploration into the application of the developed mRNA technology beyond COVID-19 for other infectious diseases relevant to LMICs, including HIV and tuberculosis, will be conducted in partnership with the South African Medical Research Council. Technical and financial support will also be delivered, based on identified needs, to technology recipients, including the priority countries of Senegal, Nigeria, Kenya and Bangladesh.

2. EXPECTED RESULTS

2.1 The overall aim of this project is to contribute to equitable access to mRNA vaccines by increasing the distribution of sustainable manufacturing capacity across LMICs, enhancing regional and inter-regional collaborations, as well as developing and empowering a local workforce. This goal will be achieved by supporting the development of the technology platform, the multilateral transfer of the technology platform to technology recipients and by providing corresponding support to technology recipient "spoke" facilities to enable technology transfer.

GAC - Released under the Access to Informations Act/
AMC - Divulgué en vertu de la loi sur l'accès à l'information

2.2 The project will support increased regional availability of mRNA vaccines for COVID-19 and explore the broader application of the developed mRNA-based technology for vaccines manufacturing towards additional infectious diseases relevant to LMICs. The goal is to achieve ultimate production at a first facility capable of reaching 50-100[1] million doses per year for the southern African region.

2.3 Intermediate Outcomes

- Established or enhanced sustainable mRNA vaccine manufacturing capacity in regions with limited or no vaccine manufacturing capacity or where vaccine manufacturing capacity exists but with different technologies.
- Increased skilled human capital, particularly amongst women, in biomanufacturing, technology transfer and regulatory capabilities.

2.4 Reporting will be provided annually and produced in coordination of the World Health Organization. Regular updates will also be provided via the mRNA Technology Transfer Hub Programme Funders Forum quarterly meetings and monthly updates. Annual reports will include:

- Summary of activities conducted at the South African hub consortium and spokes facilities;
- Key progress indicators;
- Gender-disaggregated data for human capital employed on Programme activities at the hub and participating to "spokes" training;
- Risks and mitigation with respect to the development and implementation of the project;
- Financial statements.

3. PROJECT ACTIVITIES

3.1 Canada's funding will primarily support the establishment of the mRNA Technology platform, through activities conducted by the South African Consortium such as:

- Preparation and start-up of the Hub facilities;
- Training of staff and technical assistance at the Hub facility;
- Development of the mRNA vaccine technology (process and analytical methods) by using COVID-19 as Proof of Concept at the Hub;
- Continued analysis and review of intellectual property landscape and regulatory processes;
- Research and development activities, including preclinical and clinical studies;
- Design of the technology transfer programme;
- Maintenance of the Hub facilities;

[1] To be further refined as additional facilities in the mRNA network begin production, demand for COVID-19 vaccines becomes clearer and mRNA vaccine development for additional diseases is tested.

GAC - Released under the Access to Informations Act/
AMC - Divulgué en vertu de la loi sur l'accès à l'information

- Scale-up and validation of the mRNA vaccine manufacturing platform at the first spoke (Biovac);
- Research to develop a second-generation improved mRNA technology and to apply the mRNA technology to different target diseases relevant to LMICs; and
- Other miscellaneous activities, as required.

3.2 Canada's funding will also support identified needs of recipient manufacturing facilities, including in the priority countries of Senegal, Nigeria, Kenya and Bangladesh, with activities including, but not limited to:

- Training of staff on good quality practice biomanufacturing, specifically on the mRNA technology;
- Preparation of facilities, including procurement of critical equipment, to receive mRNA technology;
- Transfer of the technology;
- Other miscellaneous activities, as required.

s.15(1) - International

s.21(1)(b)

GAC - Released under the Access to Informations Act/
AMC - Divulgué en vertu de la loi sur l'accès à l'information

BUDGET

**1- Continued support for technology transfer activities to the South African Hub (Afrigen) and spoke (Biovac)**	[redacted]
• Preparation and start up of the hub facility<br>• GMP (R&D) COVID-19 mRNA vaccine manufacturing process and analytical methods development<br>• mRNA vaccine batch production and testing for a Phase I/II clinical trial<br>• Pre-clinical and clinical Phase I/II studies	
• GMP COVID-19 mRNA vaccine manufacturing process scale-up and process and analytical methods validation<br>• mRNA vaccine production and testing for a Phase III clinical trial	
• Research activities to develop a second-generation mRNA vaccine technology and establish a pipeline of vaccine candidates for Low- Middle-Income Countries	
**2- Additional support for technology transfer activities across the spoke network***	
• Bio-manufacturing training (or similar) to empower local workforce and ensure staff readiness to receive the technology transfer	
• Procurement of critical equipment for the manufacturing of the mRNA vaccines	
**Total Direct Costs**	**CAD$ 43.15 M**
**Indirect Costs (overhead)**	**CAD$ 1.85M**
**Total estimated budget**	**CAD $ 45M**

*Funding will be provided across the network of recipient facilities, including the priority countries of Nigeria, Senegal, Kenya, and Bangladesh, based on needs identified by Medicines Patent Pool.

The program will respond to partner-driven needs to achieve increased manufacturing capacity of mRNA vaccines, maintaining the required budget flexibility to ensure all critical activities can be completed.

s.15(1) - International

s.21(1)(b)

GAC - Released under the Access to Informations Act/
AMC - Divulgué en vertu de la loi sur l'accès à l'information

Martin, Christine -MNV [She,Her | Elle]

From: Tarr, Michael -MNV [He,Him | Il]
Sent: January 25, 2023 5:01 PM
To: Khawam, Carine -PRET -DA; Martin, Christine -MNV [She,Her | Elle]; Acharya, Nina -MNV [She,Her | Elle]
Cc: Trachsel, Susanne -WEK; Clark, Andrew -WEK [He,Him | Il]; Hindle, Laird -WER [He,Him | Il]; Oliver, Jessica -WEK; Gcali, Andisiwe -PRET -DA; Mahmood, Sadia -WER; Schulz, Sara -MNV [She,Her | Elle]
Subject: RE: Country Action Plan: South Africa

Hi Carine,

Thanks for connecting and for the heads up on potential MINT-MINE trip to South Africa. This would indeed be a good opportunity to announce Canada's additional funding to the project.

Please find below the supplementary information requested. Let me know if I've missed anything – happy to provide more.

GAC funding:

- $45M total has been approved and disbursed (as of December 31)
- $30M has been announced; $15M is unannounced
- [redacted] has been allocated to the Hub (South Africa); [redacted] has been allocated to the Spokes ([redacted]

Budget:

- [redacted]
- [redacted]
- [redacted]

Briefing materials:

- MPP is putting together a comprehensive pack with high-level project and technical information (Q&A, etc.). This should be ready by end of February – I will share when it arrives.
- In the meantime, I will share updated internal project and issue briefs once approved (likely next week).

Appreciate the offer to support and facilitate connections – indeed now that funding is out the door we are aiming to engage further at country and regional levels to track discussions/developments and the broader vaccine manufacturing landscape.

Best,
Mike

From: Khawam, Carine -PRET -DA <Carine.Khawam@international.gc.ca>
Sent: January 25, 2023 7:30 AM
To: Martin, Christine -MNV [She,Her | Elle] <Christine.Martin@international.gc.ca>; Acharya, Nina -MNV [She,Her | Elle] <Nina.Acharya@international.gc.ca>
Cc: Trachsel, Susanne -WEK <Susanne.Trachsel@international.gc.ca>; Clark, Andrew -WEK [He,Him | Il] <Andrew.Clark@international.gc.ca>; Hindle, Laird -WER [He,Him | Il] <Laird.Hindle@international.gc.ca>; Oliver, Jessica -WEK <jessicac.oliver@international.gc.ca>; Gcali, Andisiwe -PRET -DA <Andisiwe.Gcali@international.gc.ca>;

GAC - Released under the Access to Informations Act/
AMC - Divulgué en vertu de la loi sur l'accès à l'information

Mahmood, Sadia -WER <Sadia.Mahmood@international.gc.ca>; Tarr, Michael -MNV [He,Him | Il] <Michael.Tarr@international.gc.ca>; Schulz, Sara -MNV [She,Her | Elle] <Sara.Schulz@international.gc.ca>
Subject: RE: Country Action Plan: South Africa

Dear Nina (as per Christine's out of office)

Responding to the message which was sent to us. We do not have any concerns to flag, as you may have heard he have been asked to prepare recommendation for a possible MINT-MINE trip to Sub-Saharan Africa, which would include South Africa. This is very much still in flux but if it goes ahead than it will certainly be an opportunity for a local announcement and site visit. I've updated the engagement to reflect this possibility and also to include other partners meetings where we are present. I had also shared last week a white paper completed by the Clinton initiative which reviewed the African vaccine manufacturing supply landscape and expansion – the findings are very much relevant, on-par, and important considerations when looking at vaccine production in SA. Our dev team in the field is small, however, if there are specific questions or concerns you'd like us to answer specific to South Africa, I'd be reach out to a few partners/key govt rep and can share any analysis / reflections coming from those discussions, but I believe they would be very much inline which the finding found in the white paper.

On the proposal itself, I would like to seek clarification on planned funding that MNV is currently considering, when I last spoke with Michael in Oct– he provided a heads-up of the PM announcement would be made at the G20, for the first of possible two top-ups with the breakdown below

- [redacted]
- [redacted]

[redacted]

From a field perspective, it would be good to know how we rank with other funders to the Hub, and also is there any briefing materials available on the hub itself, I know your team had share the Can-Give package, but didn't have specific on the mRna hub itself, if there is such a product always good for us to have as a reference

cheers
Carine

Carine Khawam
Counsellor, Head of Cooperation | Conseillère, Cheffe de la coopération
High Commission of Canada in South Africa | Haut-commissariat du Canada en Afrique du Sud
1103 Arcadia Street, Hatfield, 0083
carine.khawam@international.gc.ca
Tel: +27(0)12-422-3042 | Mobile: +27(0)82-688-3330
Follow us: | | Suivez nous: | |

Global Affairs Canada | Affaires mondiales Canada
Government of Canada | Gouvernement du Canada

Global Affairs Canada Affaires mondiales Canada

GAC - Released under the Access to Informations Act/
AMC - Divulgué en vertu de la loi sur l'accès à l'information

From: Martin, Christine -MNV [She,Her | Elle] <Christine.Martin@international.gc.ca>
Sent: January 6, 2023 4:38 PM
To: Trachsel, Susanne -WEK <Susanne.Trachsel@international.gc.ca>; Clark, Andrew -WEK [He,Him | Il] <Andrew.Clark@international.gc.ca>; Khawam, Carine -PRET -DA <Carine.Khawam@international.gc.ca>; Khawam, Carine -PRET -DA <Carine.Khawam@international.gc.ca>; Hindle, Laird -WER [He,Him | Il] <Laird.Hindle@international.gc.ca>; Oliver, Jessica -WEK <jessicac.oliver@international.gc.ca>; Fungurani, Tsitsi -PRET -DA <Tsitsi.Fungurani@international.gc.ca>; Gcali, Andisiwe -PRET -DA <Andisiwe.Gcali@international.gc.ca>
Subject: RE: Country Action Plan: South Africa

Dear colleagues,

I hope this message finds you well. I am kindly following up on the previous exchange regarding CanGIVE to ensure any comments or concerns you may have about the Action Plan or project proposals are addressed. Thank you in advance for your time, I appreciate your time reviewing the attached documents and updating the advocacy section of the Action Plan as needed. Please share any input by the end of next week, on January 13 COB.

Do not hesitate to reach out if you have questions about the initiative or the projects.

With tremendous gratitude,
Christine

From: Martin, Christine -MNV [She,Her | Elle]
Sent: December 14, 2022 11:28 AM
To: Trachsel, Susanne -WEK <Susanne.Trachsel@international.gc.ca>; Clark, Andrew -WEK [He,Him | Il] <Andrew.Clark@international.gc.ca>; Khawam, Carine -PRET -DA <Carine.Khawam@international.gc.ca>; Khawam, Carine -PRET -DA <Carine.Khawam@international.gc.ca>; Hindle, Laird -WER [He,Him | Il] <Laird.Hindle@international.gc.ca>; Oliver, Jessica -WEK <jessicac.oliver@international.gc.ca>; Fungurani, Tsitsi -PRET -DA <Tsitsi.Fungurani@international.gc.ca>; Gcali, Andisiwe -PRET -DA <Andisiwe.Gcali@international.gc.ca>
Subject: Country Action Plan: South Africa

Dear colleagues,

I am pleased to share the latest country action plan and MPP project proposal with you. In the country plan, you will notice that the amount to be allocated by MPP in-country has been updated to reflect the latest proposal/budget. Kindly let me know if you have comments or questions about the activities proposed by MPP. If you can also input into the advocacy plans by December 22 COB, it would be much appreciated.

I am also happy to report that MINE approved $117M in additional funding to ACT-A partners for CanGIVE programming earlier this month. We are now finalizing grant arrangements with partners and expect the funding to be disbursed in the coming weeks so all projects can begin soon.

Simultaneously, we are also exploring moments in Q4 to announce this additional funding and will connect with you as plans get finalized.

GAC - Released under the Access to Informations Act/
AMC - Divulgué en vertu de la loi sur l'accès à l'information

In the meantime, please do not hesitate to reach our if you have any questions. Thank you in advance for making time to provide input into the country plan.

Kindest regards,
Christine

Christine Martin (she/her/hers)
Analyst | Analyste
Global immunization and Health systems (MNV) | Vaccination mondiale et systèmes de santé (MNV)
Health and Nutrition Bureau (MND) | Direction générale de la Santé et de la Nutrition (MND)
: christine.martin@international.gc.ca
: 343-543-7913
: chat with me | clavardez avec moi

111 Promenade Sussex/ Promenade Sussex, Ottawa, Ontario, K1N 1J1
Global Affairs Canada | Affaires mondiales Canada
Government of Canada | Gouvernement du Canada

s.15(1) - International

GAC - Released under the Access to Informations Act/
AMC - Divulgué en vertu de la loi sur l'accès à l'information

Canada's Global Initiative for Vaccine Equity (CanGIVE)
Supporting COVID-19 Vaccine Roll-Out

Nigeria Country Action Plan

Canada is responding to the call for catalytic surge capacity to support COVID-19 vaccine roll-out and increase demand in countries with high vaccine-related needs. Canada's Global Initiative for Vaccine Equity (CanGIVE) is a signature, whole-of-department initiative to support country-led efforts that enhance COVID-19 vaccine delivery, demand and production, while also strengthening broader health systems and integrating COVID-19 response into routine health services. Informed by country level analysis in the 12 CanGIVE priority countries, this document outlines the proposed programming options in Nigeria. Canada's programming will provide earmarked support through ACT-Accelerator partners and will contribute to meeting Canada's ACT-Accelerator burden share consistent with Budget 2022. CanGIVE will reinforce these investments for maximum impact through strategic country-level advocacy and political engagement led by Canada's missions. This engagement will ensure Canadian visibility, while also reinforcing a "one plan" approach at country level with key COVID-19 Vaccine Delivery Partnership (CoVDP) partners.

CanGIVE NIGERIA PROGRAMMATIC PORTFOLIO

CanGIVE efforts in Nigeria will focus primarily on vaccine delivery and demand, with a secondary emphasis on increasing local vaccine production through the following projects over 24 months (2022-2024)[1].

- **UNICEF -** Global Affairs Canada will provide approximately [redacted] to UNICEF to:
  - Accelerate the implementation of the national COVID-19 vaccination strategy and advance progress towards the national target of 70% coverage by December 2022. This will include institutional capacity building to manage cold chain equipment, and additional service delivery points to provide bundled COVID-19 vaccination campaigns in hard-to-reach areas, including humanitarian contexts in the Northwest and Northeast regions.
  - Support social behaviour change interventions to reach populations targeted for vaccination campaigns, co-create and implement tailored local solutions alongside communities, the private sector and civil society and community health workers.
  - Integrate COVID-19 vaccination into routine immunization in prioritized States, including support for WASH and infection prevention and control supplies, training and management.
  - Conduct integrated campaigns with bundled services (COVID-19 vaccination, routine immunization, measles outbreak response, fIPV2 vaccination, birth registration and Vitamin A supplementation, in states under the Humanitarian Appeal for Children (Borno, Yobe, Adamawa, Zamfara, Sokoto, Kebbi and Benue states) and in outbreak-prone areas, as well as areas with high numbers of zero-dose children.
  - Expand access to Primary Health Care services for internally displaced people and host communities, including ensuring continuity of immunization services and COVID-19 vaccination during public heath outbreaks.

[1] CanGIVE programs will bolster existing bilateral COVID-19 and health systems strengthening programming that may also enhance vaccine delivery and demand generation.

s.15(1) - International

s.21(1)(b)

GAC - Released under the Access to Informations Act/
AMC - Divulgué en vertu de la loi sur l'accès à l'information

- **World Health Organization (WHO) –** Global Affairs Canada will provide approximately [redacted] to the WHO to:
    - Provide coordination and logistics support, in collaboration with UNICEF and partners, to increase COVID-19 vaccine uptake through deployment of mobile teams to vaccinate priority groups (older adults, health workers, people with comorbidities).
    - Undertake outreach to increase demand using gender-responsive communications and community engagement strategies that address barriers to vaccine uptake.
    - Integrate COVID-19 vaccination services into primary health care, including at clinics for patients living with HIV, tuberculosis, cancer, diabetes, and cardiovascular diseases to reinforce health system capacity.
    - Strengthen Integrated Disease Surveillance Reporting (IDSR) by improving data management and monitoring capacity.

- **Medicines Patent Pool (MPP) –** Global Affairs Canada will provide [redacted] to:
    - Support mRNA Vaccine Technology Transfer Hub efforts to facilitate training, technical support and technology transfer to Biovaccines Nigeria Ltd. in Nigeria. Efforts will aim to establish a sustainable production model and ensure successful implementation of mRNA technology.

CanGIVE ADVOCACY ENGAGEMENT STRATEGY

Canada will engage in national, regional and global advocacy to support Nigeria in their efforts to increase vaccination coverage towards national targets, enhance vaccine confidence and scale-up vaccine production for longer-term pandemic preparedness. Advocacy will align with existing country-led coordination mechanisms, including the COVID-19 Vaccine Delivery Partnership 'One Plan' and bilateral efforts from key donors.

Engagement Opportunity	Date
Ministerial Engagement<br><br>• **MINE** to participate in site visit and/or meet with partners during Ministerial travel to the region.	TBD
Canadian Mission and Senior Official Engagement<br><br>• **Head of Cooperation** to participate in regular CoVDP meetings to engage on financing prioritizing and operational vaccine delivery efforts being facilitated by the CoVDP, country government and donors.<br><br>• **Head of Cooperation** to participate in the Development Partners Heads of Agencies meeting, including key bilateral donors such as UNICEF and WHO, as well as the One UN COVID-19 Basket Fund steering committee.<br><br>• **Head of Mission/Head of Cooperation** bilateral meeting with Minister of Health to discuss COVID-19 response and integration within primary health care/routine immunization.	Monthly<br><br>Monthly<br><br>As required/ biannual

GAC - Released under the Access to Informations Act/
AMC - Divulgué en vertu de la loi sur l'accès à l'information

• **Canadian Mission** to participate in technical working groups to including the Development Partnership Group for Health.<br>• **HQ** participation in Senior Officials level meeting and site visit (e.g. Gavi annual high-level missions).	Monthly<br><br>Annual

s.15(1) - International

GAC - Released under the Access to Informations Act/
AMC - Divulgué en vertu de la loi sur l'accès à l'information

Canada's Global Initiative for Vaccine Equity (CanGIVE)
Supporting COVID-19 Vaccine Roll-Out

Senegal Country Action Plan

Canada is responding to the call for catalytic surge capacity to support COVID-19 vaccine roll-out and increase demand in countries with high vaccine-related needs. Canada's Global Initiative for Vaccine Equity (CanGIVE) is a signature, whole-of-department initiative to support country-led efforts that enhance COVID-19 vaccine delivery, demand and production, while also strengthening broader health systems and integrating COVID-19 response into routine health services. Informed by country level analysis in the 12 CanGIVE priority countries, this document outlines the proposed programming options in Senegal. Canada's programming will provide earmarked support through ACT-Accelerator partners and will contribute to meeting Canada's ACT-Accelerator burden share consistent with Budget 2022. CanGIVE will reinforce these investments for maximum impact through strategic country-level advocacy and political engagement led by Canada's missions. This engagement will ensure Canadian visibility, while also reinforcing a "one plan" approach at country level with key COVID-19 Vaccine Delivery Partnership (CoVDP) partners.

CanGIVE SENEGAL PROGRAMMATIC PORTFOLIO

CanGIVE efforts in Senegal will focus primarily on vaccine delivery and demand generation with a secondary emphasis on increasing local vaccine production through the following projects over 24 months (2022-2024).[1]

- **UNICEF** – Global Affairs Canada will provide approximately [redacted] to UNICEF to:
    - Support operational costs and provide technical assistance for integrated COVID-19 and routine immunization delivery, including enhanced logistics and equipment, cold chain storage, vaccine and waste management, health workforce training and outreach activities.
    - increase demand for COVID-19 vaccination amongst healthcare workers, the elderly and people with co-morbidities through risk communication and community engagement, mass and digital communications, partnerships with traditional and religious leaders, youth and community-based organizations.
    - Support the upgrade of Infection Prevention and Control and WASH facilities, training of health staff, provision of supplies and strengthening of vaccine delivery monitoring and evaluation systems.

- **World Health Organization (WHO)** – Global Affairs Canada will provide approximately Global Affairs Canada will provide approximately [redacted] to the WHO to:
    - Provide coordination and logistics support, in collaboration with UNICEF and partners, to increase COVID-19 vaccine uptake through deployment of mobile teams to vaccinate priority groups (older adults, health workers, people with comorbidities).
    - Undertake outreach to increase demand using gender-responsive communications and community engagement strategies that address barriers to vaccine uptake.

[1] CanGIVE programs will bolster and complement existing bilateral COVID-19 and health systems strengthening programming that may also enhance vaccine delivery and demand generation.

s.15(1) - International

s.21(1)(b)

GAC - Released under the Access to Informations Act/
AMC - Divulgué en vertu de la loi sur l'accès à l'information

  - Integrate COVID-19 vaccination services into primary health care, including at clinics for patients living with HIV, tuberculosis, cancer, diabetes, and cardiovascular diseases to reinforce health system capacity.
  - Strengthen Integrated Disease Surveillance Reporting (IDSR) by improving data management and monitoring capacity.

- **Medicines Patent Pool (MPP) –** Global Affairs Canada will provide [redacted] to:
  - Support mRNA Vaccine Technology Transfer Hub efforts to facilitate training, technical support and technology transfer to the Institut Pasteur in Dakar, Senegal. Efforts will aim to establish a sustainable production model and ensure successful implementation of mRNA technology.

CanGIVE ADVOCACY ENGAGEMENT STRATEGY

Canada will engage in national, regional and global advocacy to support in Senegal in their efforts to increase vaccination coverage towards national targets, enhance vaccine confidence and/or scale-up vaccine production for longer-term pandemic preparedness. Advocacy will align with existing country-led coordination mechanisms, including the COVID-19 Vaccine Delivery Partnership 'One Plan' and bilateral efforts from key donors. Canada's CanGIVE contributions are welcome by the Government of Senegal, noting that the investment space includes funding from the EU, World Bank, US, and Japan.

Engagement Opportunity	Date
Ministerial Engagement<br><br>• **MINE** to participate in site visit and/or meet with partners during Ministerial travel to the region.	TBD
Canadian Mission and Senior Official Engagement<br><br>• **Senegal Program** to participate in regular CoVDP meetings to engage on financing, prioritization and operations of vaccine delivery efforts being facilitated by the CoVDP, country government and donors; provide visibility to Canadian funding.<br>• **Senegal Program** to participate in Financial and Technical Partner Coordination group on Vaccination, highlighting continued need for increased COVID-19 vaccine roll-out and demand generation efforts, including strengthening existing health care systems to support delivery.<br>• **Head of Cooperation** bilateral meeting with Ministry of Health (Minister of Secretary General) and key CanGIVE programming partners to discuss COVID-19 response and integration within primary health care/routine immunization. Opportunity to provide visibility to Canadian funding.	Bi-monthly<br><br>Bi-monthly<br><br>Q3/annually or bi-annually

s.15(1) - International

GAC - Released under the Access to Informations Act/
AMC - Divulgué en vertu de la loi sur l'accès à l'information

Canada's Global Initiative for Vaccine Equity (CanGIVE) Supporting COVID-19 Vaccine Roll-Out

Bangladesh Country Action Plan

Canada is responding to the call for catalytic surge capacity to support COVID-19 vaccine roll-out and increase demand in countries with high vaccine-related needs. Canada's Global Initiative for Vaccine Equity (CanGIVE) is a signature, whole-of-department initiative to support country-led efforts that enhance COVID-19 vaccine delivery, demand and production, while also strengthening broader health systems and integrating COVID-19 response into routine health services. Informed by country level analysis in the 12 CanGIVE priority countries, this document outlines the proposed programming options in Bangladesh. Canada's programming will provide earmarked support through ACT-Accelerator partners and will contribute to meeting Canada's ACT-Accelerator burden share consistent with Budget 2022. CanGIVE partners will undertake a collaborative approach with Canadian missions to leverage these investments for maximum impact, ensuring Canadian visibility, while also reinforcing a "one plan" approach at country level with key COVAX and humanitarian partners.

CanGIVE BANGLADESH PROGRAMMATIC PORTFOLIO

CanGIVE efforts in Bangladesh will focus on scaling up vaccination delivery, primary health care and increasing vaccine confidence amongst humanitarian and at-risk populations with a complementary emphasis on sustainable local vaccine production through the following projects over 24 months (2022-2024).[1]

- **UNICEF** – Global Affairs Canada will provide [redacted] to UNICEF to:
    - Increase COVID-19 vaccination coverage, especially among the Rohingya refugees, the elderly, at-risk and climate vulnerable populations among Bangladesh' population. This will include activities such as booster dose vaccination campaigns, social behaviour change communication to promote COVID-19 vaccination, support for the upgrading, installation and maintenance of cold chain equipment and transportation alongside capacity building for cold chain service providers, and support for quality data collection and monitoring.
    - Support essential gender responsive MNCAH services and infection prevention and control among Rohingya Refugees and Bangladesh' population including primary health care for refugees and outbreak response, community capacity building for gender responsive emergency obstetric and neo-natal care, gender responsive MNCAH community engagement and supporting government capacity for tracking maternal and newborn data.
    - Strengthen health system resilience and essential service delivery in flood-affected areas. This will include logistics support for mobile medical teams, procurement of medicines to support disease outbreaks (especially acute watery diarrhoea), training of health care workers on management of acute watery diarrhoea, referrals of pregnant mothers in hard-to-reach locations, scale-up of routine immunization and COVID-19 vaccination activities, and assessment of damage to health facilities and subsequent renovation of facilities.

[1] CanGIVE programs will bolster existing bilateral COVID-19 and health systems strengthening programming that may also enhance vaccine delivery and demand generation.

s.15(1) - International

s.21(1)(b)

GAC - Released under the Access to Informations Act/
AMC - Divulgué en vertu de la loi sur l'accès à l'information

- **Medicines Patent Pool (MPP) –** Global Affairs Canada will provide [redacted] to MPP to:
    - Support mRNA Vaccine Technology Transfer Hub efforts to facilitate training, technical support and technology transfer to Incepta Vaccine Ltd in Bangladesh. Efforts will aim to establish a sustainable production model and ensure successful implementation of mRNA technology.

CanGIVE ADVOCACY ENGAGEMENT STRATEGY

Canada will engage in national, regional and global advocacy to support Bangladesh in their efforts to increase vaccination coverage amongst refugee and at-risk populations. Advocacy will align with Canadian priorities, existing country-led humanitarian coordination mechanisms and bilateral efforts from key donors.

Engagement Opportunity	Date
Ministerial Engagement<br>• **MINE** to participate in site visit and meet with partners during Ministerial travel.	TBD
Canadian Mission and Senior Official Engagement<br>• **Dhaka-DA** to participate in meetings as part of the Health Development Partners Local Consultative Group to engage on financing, prioritization and operational vaccine delivery efforts being facilitated by UN agencies, country government and donors.<br>• **Head of Mission/Head of Cooperation** bilateral meeting with UNICEF and WHO Country Representatives and, as possible, the Secretary for the Ministry of Health to discuss COVID-19 response and integration within primary health care/routine immunization, especially for humanitarian populations.<br>• Continued Canadian participation at a **technical level** in the Health Population Nutrition DP Consortium and Bangladesh Preparedness and Response Plan working group<br>• Potential for high-level site visit TBD	Monthly<br>As required<br>Ad Hoc<br>TBD
