# Supplementary material for: ‘Our project, your problem?’ A case study of the WHO’s mRNA technology transfer programme in South Africa
Source: PLOS Glob Public Health. 2024 Sep 23;4(9):e0003173. doi: 10.1371/journal.pgph.0003173 (PMC11419367; doi:10.1371/journal.pgph.0003173)
Supplement: S1 Table — (DOCX) [file pgph.0003173.s003.docx]

|  | **Agreement Particulars** | | | | | **Key IP Provisions** | | | | | **Other Equitable Access Commitments** | | | |
| --- | --- | --- | --- | --- | --- | --- | --- | --- | --- | --- | --- | --- | --- | --- |
|  | *Party/Parties*  *(Other than MPP)* | *Type of Agreement* | *Date of Execution (MM/DD/YY)* | *Key Terms of the Agreement* | *Principal Consideration Provided (Other than IP)* | *Royalty-free License to Consortium IP from MPP?* | *Royalty-free, worldwide Grant-Back Clause?* | *“Reasonable Efforts” to Secure License to Third-Party IP?* | *MPP to Provide “IP Analysis”?* | *Warranty of Non-Infringement for use of IP?* | *“Cost-Plus” for PHEIC Products?* | *Pricing Clause for non-PHEIC Products?* | *Allocate up to 10% of Products targeting a PHEIC?* | *Seek WHO Pre-Qualification?* |
| **Architects** | World Health Organization | MoU* | 01/01/24 | The MoU is non-legally binding. No financial responsibilities. | 7,361,072 EUR† (7,719,299 USD) | N/A | N/A | N/A | 🗸# | N/A# | N/A | N/A | N/A | N/A |
| **Consortium** | Afrigen | Grant | 01/21/22 | Grant is for “non-commercial purposes only” | 39 Million EUR (40,897,938 USD) | N/A | 🗸 | 🗴 | 🗴 | 🗴% | 🗴 | 🗴 | 🗴 | 🗴 |
|  | Biovac | Grant & Technology Transfer | 08/04/22 | IP License from MPP Limited to LMICs | 303 Million ZAR (15,264,965 USD) | N/A | 🗸 | 🗸 | 🗸# | 🗴 | 🗸 | 🗴 | 🗸 | 🗸 |
|  | SAMRC | Grant | 10/14/22 | Grant is for “non-commercial purposes only” | 26.6 Million ZAR (1,340,093 USD) | N/A | 🗴‡ | 🗴 | 🗴 | 🗴 | 🗴 | 🗴 | 🗴 | 🗴 |
| P**artners/Spokes** | Incepta  (Bangladesh) | Technology Transfer | 08/22/22 | IP License from MPP Limited to LMICs | Technology Transfer Packages 1-3 | 🗸 | 🗸 | 🗸 | 🗸 | 🗴 | 🗸 | 🗴 | 🗸 | 🗸 |
|  | Torlak Institute  (Serbia) | Technology Transfer | 08/23/22 | IP License from MPP Limited to LMICs | Technology Transfer Packages 1-3 | 🗸 | 🗸 | 🗸 | 🗸 | 🗴 | 🗸 | 🗴 | 🗸 | 🗸 |
|  | BioGeneric Pharma  (Egypt) | Technology Transfer | 08/29/22 | IP License from MPP Limited to LMICs | Technology Transfer Packages 1-3 | 🗸 | 🗸 | 🗸 | 🗸 | 🗴 | 🗸 | 🗴 | 🗸 | 🗸 |
|  | Biological E.  (India) | Technology Transfer | 08/30/22 | IP License from MPP Limited to LMICs | Technology Transfer Packages 1-3 | 🗸 | 🗸 | 🗸 | 🗸 | 🗴 | 🗸 | 🗴 | 🗸 | 🗸 |
|  | Institut Pasteur de Tunis  (Tunisia) | Technology Transfer | 09/22/22 | IP License from MPP Limited to LMICs | Technology Transfer Packages 1-3 | 🗸 | 🗸 | 🗸 | 🗸 | 🗴 | 🗸 | 🗴 | 🗸 | 🗸 |
|  | Darnytsia  (Ukraine) | Technology Transfer | 09/28/22 | IP License from MPP Limited to LMICs | Technology Transfer Packages 1-3 | 🗸 | 🗸 | 🗸 | 🗸 | 🗴 | 🗸 | 🗴 | 🗸 | 🗸 |
|  | Polyvac  (Viet Nam) | Technology Transfer | 09/29/22 | IP License from MPP Limited to LMICs | Technology Transfer Packages 1-3 | 🗸 | 🗸 | 🗸 | 🗸 | 🗴 | 🗸 | 🗴 | 🗸 | 🗸 |
|  | Biovaccines Nigeria Ltd  (Nigeria) | Technology Transfer | 10/26/22 | IP License from MPP Limited to LMICs | Technology Transfer Packages 1-3 | 🗸 | 🗸 | 🗸 | 🗸 | 🗴 | 🗸 | 🗴 | 🗸 | 🗸 |
|  | Sinergium Biotech  (Argentina) | Technology Transfer | 01/24/23 | IP License from MPP Limited to LMICs | Technology Transfer Packages 1-3 | 🗸 | 🗸 | 🗸 | 🗸 | 🗴 | 🗸 | 🗴 | 🗸 | 🗸 |
|  | Institut Pasteur Dakar  (Senegal) | Technology Transfer | 02/07/23 | IP License from MPP Limited to LMICs | Technology Transfer Packages 1-3 | 🗸 | 🗸 | 🗸 | 🗸 | 🗴 | 🗸 | 🗴 | 🗸 | 🗸 |
|  | PT Bio Farma  (Indonesia) | Technology Transfer | 03/03/23 | IP License from MPP is “worldwide” | Technology Transfer Packages 1-3 | 🗸 | 🗸 | 🗸 | 🗸 | 🗴 | 🗸 | 🗴 | 🗸 | 🗸 |
|  | NIH Pakistan  (Pakistan) | Technology Transfer | 9/01/23 | IP License from MPP Limited to LMICs | Technology Transfer Packages 1-3 | 🗸 | 🗸 | 🗸 | 🗸 | 🗴 | 🗸 | 🗴 | 🗸 | 🗸 |

* The Memorandum of Understanding (MoU) is, in contrast to the other programme agreements, a “non-legally binding” document, which “represents the framework for future discussions between and activities by the Parties in relation to the Collaboration.” It sets out roles and responsibilities that are shared between WHO and MPP, as well as roles and responsibilities that are specific to each organization.

† This figure encompasses sums conveyed under three “Grant Letter of Agreements,” in the amounts of 1,130,072, 1,000,000, and 5,231,000.

‡The MPP-SAMRC Grant Agreement provides for a “non-exclusive, transferable, sublicensable, irrevocable, worldwide, license to practice and have practiced the data and Inventions for the purposes of fulfilling its mission to facilitate the development and affordable and equitable access of mRNA technologies in low- and middle-income countries (as defined by the World Bank), *which license may include a royalty sacrifice*.” (emphasis added)

#The MoU between WHO and MPP is one of the only two documents underpinning the programme, which explicitly mentions “freedom to operate.” Specifically, among the responsibilities assigned exclusively to MPP is: “Provide IP analysis and *commission freedom to operate assessments for the Partners, as necessary*.” (emphasis added) In contrast, MPP’s technology transfer agreements with partners (with the exception of the Biovac agreement) stipulate that MPP will “[p]rovide IP analysis on the Technology, as practicable and appropriate.” The Biovac technology transfer agreement obligates MPP to “[p]rovide IP analysis on the Technology, as practicable and appropriate *and endeavor to provide better visibility on freedom to operate analyses in the Territory.*” (emphasis added) Given that the MoU is a non-legally binding document whereas the partner agreements are legally-binding contracts, MPP is not obligated, strictly speaking, to commission freedom to operate analyses to participating manufacturers. Further, the technology transfer agreements also include a provision, which states that “nothing in this Agreement shall be construed as a warranty that [a partner’s] use of the Technology, Afrigen Rights or Biovac Rights [*i.e.*, the IP that the partners will receive by virtue of taking part in the programme] will not infringe any patent rights or other IP rights of any Third Party.”

%While neither Afrigen nor MPP makes any warranties regarding IP infringement in the Grant Agreement, Afrigen is required to “immediately give notice to MPP if Afrigen…becomes aware of, or if Afrigen receives notice from any third party on: (a) any infringement of the background intellectual property and/or Invention; or (b) any claim by a third party that an action carried out under the Project infringes the intellectual property or other rights of any third party.”
