# Supplementary material for: ‘Our project, your problem?’ A case study of the WHO’s mRNA technology transfer programme in South Africa
Source: PLOS Glob Public Health. 2024 Sep 23;4(9):e0003173. doi: 10.1371/journal.pgph.0003173 (PMC11419367; doi:10.1371/journal.pgph.0003173)
Supplement: S2 Table — (DOCX) [file pgph.0003173.s004.docx]

**S4 Table.** mRNA Related Patent Filings, including filed, rejected, withdrawn, and granted patents: January 2006 – December 2022.

| **Year** | **AR** | **BD** | **BR** | **EG** | **ID** | **IN** | **KE** | **NG** | **PK** | **SN** | **RS** | **ZA** | **TN** | **UA** | **VN** | **Total** |
| --- | --- | --- | --- | --- | --- | --- | --- | --- | --- | --- | --- | --- | --- | --- | --- | --- |
| 2006 |  |  |  |  | 1 |  |  |  |  |  | 9 |  |  |  |  | 10 |
| 2007 |  |  |  |  |  |  |  |  |  |  |  |  |  |  |  | 0 |
| 2008 |  |  |  |  |  |  |  |  |  |  |  |  |  |  |  | 0 |
| 2009 |  |  |  |  | 2 |  |  |  |  |  | 2 |  |  |  |  | 4 |
| 2010 |  |  |  |  |  |  |  |  |  |  |  |  |  |  |  | 0 |
| 2011 |  |  | 2 |  | 1 |  |  |  |  |  | 9 |  |  |  |  | 12 |
| 2012 |  |  | 1 |  | 1 |  |  |  |  |  | 7 | 1 |  |  |  | 10 |
| 2013 |  |  | 5 |  | 4 |  |  |  |  |  | 11 | 1 |  |  |  | 21 |
| 2014 |  |  |  |  | 2 |  |  |  |  |  | 1 | 4 |  |  |  | 7 |
| 2015 |  |  | 1 |  | 1 |  |  |  |  |  | 11 | 1 |  |  |  | 14 |
| 2016 | 1 |  |  |  |  |  |  |  |  |  | 16 |  |  |  |  | 17 |
| 2017 |  |  | 1 |  |  |  |  |  |  |  | 1 | 1 |  |  |  | 3 |
| 2018 |  |  |  |  |  |  |  |  |  |  |  |  |  |  |  | 0 |
| 2019 |  |  |  |  | 1 |  |  |  |  |  | 2 |  | 2 |  |  | 5 |
| 2020 |  |  | 3 | 3 | 3 | 3 |  |  |  |  | 3 | 3 | 3 | 3 | 3 | 27 |
| 2021 | 2 |  | 4 | 1 | 3 |  |  |  |  |  | 6 | 4 | 2 |  | 1 | 23 |
| 2022 |  |  | 1 |  |  |  |  |  |  |  | 3 |  | 2 |  |  | 6 |
| **Total** | 3 | 0 | 18 | 4 | 19 | 3 | 0 | 0 | 0 | 0 | 81 | 15 | 9 | 3 | 4 | **159** |

*Notes:* (1) The columns are organized by country taking part in the mRNA programme and the two-letter codes (used by the patent system) correspond, respectively, to Argentina, Bangladesh, Brazil, Egypt, Indonesia, India, Kenya, Nigeria, Pakistan, Senegal, Serbia, South Africa, Tunisia, Ukraine, and Vietnam. (2) This data was abstracted from MPP’s VaxPal database, with Research Assistance provided by Morris Odeh.
