# Supplementary material for: ‘Our project, your problem?’ A case study of the WHO’s mRNA technology transfer programme in South Africa
Source: PLOS Glob Public Health. 2024 Sep 23;4(9):e0003173. doi: 10.1371/journal.pgph.0003173 (PMC11419367; doi:10.1371/journal.pgph.0003173)
Supplement: Attachment — Submitted filename: PGPH-D-24-00260 - Response Letter - June2.24.docx [file pgph.0003173.s005.docx]

Journal Requirements:

1. Please review your reference list to ensure that it is complete and correct. If you have cited papers that have been retracted, please include the rationale for doing so in the manuscript text, or remove these references and replace them with relevant current references. Any changes to the reference list should be mentioned in the rebuttal letter that accompanies your revised manuscript. If you need to cite a retracted article, indicate the article’s retracted status in the References list and also include a citation and full reference for the retraction notice.

***We have reviewed our reference list and to the best of our knowledge it does not contain any retracted papers.***

2. Please amend your detailed Financial Disclosure statement. This is published with the article. It must therefore be completed in full sentences and contain the exact wording you wish to be published.

a. State the initials, alongside each funding source, of each author to receive each grant.
b. If any authors received a salary from any of your funders, please state which authors and which funders.

***We have revised our Financial Disclosure statement to read as follows:***

***One author (MH) holds a Chair in Applied Public Health, funded by the Canadian Institutes of Health Research (CIHR) and Public Health Agency of Canada (PHAC). This Chair carries a salary award as well as funding for research activities. However, neither CIHR nor PHAC played any role whatsoever in the design of the present study, data collection, analysis or writing process.***

3. We have noticed that you have uploaded Supporting Information files, but you have not included a list of legends. Please add a full list of legends for your Supporting Information files after the references list.

***We have added a list of Supplementary Information files following our conclusion and added these four files to our revised submission. As noted below, if one of the tables is in your view better placed within the body of the manuscript as opposed to a supplementary file, we are happy to defer to the editors’ preference.***

4. In the online submission form, you indicated that "While many of the interview participants took part in our study 'on the record', we did not ask if we could publicly share interview transcripts in their entirety. The data collected through semi-structured interviews with human participants will therefore only be made available upon request and subject to the approval of the university's research ethics board. All PLOS journals now require all data underlying the findings described in their manuscript to be freely available to other researchers, either 1. In a public repository, 2. Within the manuscript itself, or 3. Uploaded as supplementary information.
This policy applies to all data except where public deposition would breach compliance with the protocol approved by your research ethics board. If your data cannot be made publicly available for ethical or legal reasons (e.g., public availability would compromise patient privacy), please explain your reasons by return email and your exemption request will be escalated to the editor for approval. Your exemption request will be handled independently and will not hold up the peer review process, but will need to be resolved should your manuscript be accepted for publication. One of the Editorial team will then be in touch if there are any issues.

***Thank you for this information. We did not contemplate sharing interview data and transcripts in their entirety as part of a publication when we sought approval from our respective ethics boards to conduct this qualitative study. Accordingly, we did not ask participants for their permission to publicly share interview transcripts. To do so, we would need to amend our protocol, submit it to our respective ethics boards for review, and contact our participants for consent to share interview transcripts.***

***This would not only consume a significant amount of time, but also potentially undermine the integrity of our findings. By way of background, our participants are generally in positions of relative power. When they gave consent to participate in an interview, they were given a time-limited window (two weeks) within which to withdraw their participation from the study. In our experience, such a limited window for withdrawal is appropriate for participants in positions of relative power; otherwise, there is a significant risk that participants may withdraw several weeks after participating, in turn, undermining our data analysis and creating a risk that the contributions that other participants (who chose to remain in the study) have made to the knowledge generation process will not be publishable due to participant withdrawal many weeks after data collection has been completed. Indeed, the research ethics board reviewed this time-limitation for withdrawal in our protocol and agreed that it was an appropriate way of managing the risks and benefits related to participating in our research study.***

***Accordingly, we would respectfully ask that the Editor grant an exemption for sharing interview transcripts. Further, sharing transcripts would also risk breaching the privacy of the participants who agreed to take part in our study provided their identities were kept confidential. For this reason, too, we believe an exemption is warranted.***

Reviewers' comments:

Reviewer's Responses to Questions

**Comments to the Author**

1. Does this manuscript meet PLOS Global Public Health’s [publication criteria](https://journals.plos.org/globalpublichealth/s/criteria-for-publication)? Is the manuscript technically sound, and do the data support the conclusions? The manuscript must describe methodologically and ethically rigorous research with conclusions that are appropriately drawn based on the data presented.

Reviewer #1: Yes

Reviewer #2: Yes

2. Has the statistical analysis been performed appropriately and rigorously?

Reviewer #1: N/A

Reviewer #2: N/A

3. Have the authors made all data underlying the findings in their manuscript fully available (please refer to the Data Availability Statement at the start of the manuscript PDF file)?

The [PLOS Data policy](https://journals.plos.org/globalpublichealth/s/data-availability) requires authors to make all data underlying the findings described in their manuscript fully available without restriction, with rare exception. The data should be provided as part of the manuscript or its supporting information, or deposited to a public repository. For example, in addition to summary statistics, the data points behind means, medians and variance measures should be available. If there are restrictions on publicly sharing data—e.g. participant privacy or use of data from a third party—those must be specified.

Reviewer #1: Yes

Reviewer #2: Yes

4. Is the manuscript presented in an intelligible fashion and written in standard English?

PLOS Global Public Health does not copyedit accepted manuscripts, so the language in submitted articles must be clear, correct, and unambiguous. Any typographical or grammatical errors should be corrected at revision, so please note any specific errors here.

Reviewer #1: Yes

Reviewer #2: Yes

5. Review Comments to the Author

Please use the space provided to explain your answers to the questions above. You may also include additional comments for the author, including concerns about dual publication, research ethics, or publication ethics. (Please upload your review as an attachment if it exceeds 20,000 characters)

Reviewer #1: This is an important paper that describes the attempt to circumvent the control that the large pharmaceutical companies currently have over R&D and access with respect to new technologies especially those based on mRNA. The paper does an in-depth evaluation of the potential and limitations of the Afrigen model and hopefully the analysis contained in this paper will lead to a re-evaluation of that model.

There are a number of places where I would like to see more detail provided or an expansion of the issues raised.

1. Was there an interview guide and if so, was it pilot tested? If there was an interview guide it should be included as an appendix.

***We did use an interview guide throughout our research. Consistent with a situational analysis approach, however, we modified and added to our interview guide in response to new lines of inquiry and topics that arose as the research unfolded. Through the process of mapping our findings from interviews with documentary sources, we sought to corroborate and triangulate key themes as they emerged. Given the dynamic nature of our data collection process, which, again, is envisaged by situational analysis, we did not pilot test our interview guide because we expected that it would evolve over the course of data collection. That said, if the editors deem it necessary, we are happy to provide our *initial* interview guide as an appendix or supplementary file.***

2. Page 3: Is Afrigen a for-profit company? The authors answer this question in Figure 1 but I think that it should be mentioned at the start of the paper.

***We have edited the first line of the paper to make this clear. The line now reads:*** “In June 2021 Afrigen Biologics, a for-profit company based in Cape Town, South Africa set out to change the global landscape of biopharmaceutical production.”

3. Was the WHO involved in helping to issue voluntary licenses, e.g., the AstraZeneca license to India's Serum Institute?

***It is our understanding that the WHO was indeed involved in setting up the license between AstraZeneca and the Serum Institute of India. However, other types of vaccines were not part of our core focus on the mRNA programme. We therefore do not think it’s appropriate to add this information.***

4. The authors might want to put some of their analysis about governance colonialism into the context of the governance of other similar initiatives and point out the prominence of organizations from the Global North - see Tucker et al. Science 2008;320:1016-1017.

***Thank you for this suggestion and reference. As we note in response to a comment by Reviewer 2 below, we have tried to show—through our findings—the ways in which countries in the global North (through their measured funding of the mRNA programme) and institutions in Geneva (especially MPP) raise this concern of colonialism. That said, the mRNA programme remains in flux and the very recent effort to build R&D Consortia holds promise (as we note in the conclusion) in terms of empowering LMIC based manufacturers to work together. For this reason, it is our strong preference to phrase the concern around colonialism gently. That’s why we note that the “decolonial aspirations” of the programme are, as yet, undetermined. Subject to further input from the editors, we hope this strikes the right balance. We have, consistent with Reviewer 2’s suggestions, expanded the discussion section of the paper, where we cite Tucker et al., among other sources, to underscore the need to empower LMIC manufacturers taking part in the programme.***

5. Page 5: How did the authors get access to the documents that they analyzed? Did they have to sign some kind of confidentiality agreement? How many people extracted information from the documents? If more than one how were discrepancies resolved and if only one person what steps were taken to guard against biases?

***All of the documents that we analysed as part of the study were either publicly available on a website (e.g., Medicine Patent Pool’s website) or obtained by request under Canada’s Access to Information (ATI) Act, which makes “records” held by the government accessible. As detailed in our supplementary materials (which we have further clarified in this revised submission by adding legends and an explanatory note about the process), we filed an ATI request in April 2023 and later received a number of government records, which we analysed. We did not sign a confidentiality agreement in respect of any of the documents that we analysed as, by virtue of their disclosure, these documents can now be considered part of the public domain. (In previously published research, we have similarly made documents obtained through an ATI request publicly available as a supplementary file accompanying a published research article. See, for e.g.,*** [***https://academic.oup.com/jlb/article/7/1/lsz019/5706941#supplementary-data***](https://academic.oup.com/jlb/article/7/1/lsz019/5706941#supplementary-data)***)***

***Both researchers involved directly in the project analysed all of the documents and differences in interpretation (all minor in nature) were resolved through discussion between the two. We have added a sentence to this effect to the paragraph in question on p. 5.***

***In addition, after submission to PLOS GPH we became aware that the technology transfer agreements signed by each individual partner in the mRNA programme have now been posted on MPP’s website. We have since analysed those individual agreements and made some changes to our findings as a result of this added documentary data. This necessitated some additional changes to the paragraph under the heading ‘Document Analysis’ on pp. 5-6 as well as some other changes (which we explain below) in other sections of the paper.***

***The revised paragraph on pp. 5-6 now reads: “***Multiple types of documents were analyzed by both researchers; a few minor inconsistencies in interpretation occurred but were resolved through discussion. The first type of document was a range of legal documents that codify the relationships between different actors in the mRNA programme, which the WHO tasked MPP with drafting and implementing. These “programme agreements”(20) are in place between MPP and the three principal members of the South African “consortium”, that is, Afrigen, another Cape Town-based vaccine manufacturer called Biovac, and the South African Medical Research Council (SAMRC). The “technology transfer template agreement,” which ~~is available on MPP’s website and~~ served as the basis for negotiations with LMIC manufacturer partners to the hub ~~was also analyzed. According to MPP’s website~~, as well as the finalized agreements between MPP and thirteen of the fifteen programme “partners” that have signed a technology transfer agreement to date, all of which are publicly available from MPP’s website (accessed: March 30, 2024), were also analyzed. (Only Bio-Manguinhos (Brazil) and BiovaX (Kenya) have not signed such an agreement.) ~~(The finalized technology transfer agreements between MPP and the 13 partners are not publicly available.)~~ Additionally, research agreements shared by interview participants were analyzed, including a funding agreement between scientists at the University of Cape Town and the SAMRC, a research collaboration agreement between the United States’ (US) National Institute of Allergy and Infectious Diseases (NIAID, a component of the National Institutes of Health (NIH)) and Afrigen, as well as sample clauses from Afrigen’s collaboration agreements with entities outside the programme. Powerpoint presentations and other information shared at the programme’s inaugural meeting held in Cape Town, April 17-21, 2023, as well as a regional meeting in Bangkok, Thailand, October 31 – November 1, 2023 were also incorporated into the study. To gain insight into the relationship between countries sponsoring the programme and WHO/MPP, an access to information (ATI) request was filed with the Canadian government, which is the second highest funder of the mRNA programme. Our ATI request yielded 153 pages of correspondence, agreements, and other documentation that we incorporated into our analysis. (*see* S1_Letter and S2_Document for further details about our request and the corresponding disclosure package) Finally, a dataset of patent applications as well as withdrawn and granted patents, compiled and made publicly available by MPP (21) was analyzed to understand the evolving patent landscape related to mRNA technologies in South Africa and other LMICs tied to the programme.”

6. Page 5: What role is the Canadian government playing in Afrigen's initiative? The authors have not mentioned Canadian involvement previously.

***Thank you for noting this omission. We have added a clause to the sentence in question to explain that the Canadian government is the second largest funder of the mRNA programme. (The revised sentence is highlighted in the paragraph above in relation to the previous comment from this reviewer.) We also note this fact later in the manuscript.***

7. Page 11: Was there also opposition to C-TAP from HICs or just indifference?

***There were reports of opposition from industry towards C-TAP, so we have added a reference to bolster this claim.***

***The sentence on p. 11 now reads: “***However, as industry opposition to C-TAP grew because of the threat that it posed to IP-holders’ control over COVID-19 interventions,(27) then-WHO assistant director general (ADG) of access to medicines and health products,…***”***

8. Page 18: I'm confused here about programme partners' access to technology royalty free. In the preceding few paragraphs the authors only mention technology transfer to LMIC partners and from partners to MPP. Where do “programme partners” fit in? Are these the spokes?

***We appreciate the reviewer’s confusion about the wording of the sentence, especially given the number of different actors involved. In this specific instance, we intended to refer exclusively spokes/partners in other LMICs that are part of the mRNA programme. We have revised the sentence in question to make this more clear. It now reads (on p. 19): “***His colleague leading Bio-Manguinhos’ mRNA vaccine project, Patricia Neves, also intimates that the idea that technology developed by Bio-Manguinhos, using funding from the Brazillian government (as opposed to funding from the mRNA programme) would flow to manufacturers from participating LMICs, which in some cases, are for-profit commercial entities, without anything in return is an “injustice.”(PN)***”***

***In addition to this change, we have also created a new Table that summarizes many of the important features of the various legal agreements underpinning the programme. This Table is large in size due to the sheer number of agreements and salient features they contain. We defer to the editors as to whether such a Table would be better included as another supplementary file given its size or incorporated directly into the paper. For the time being, we have added it as a supplementary file (S3_Table) to our revised submission while also noting in several places where the table supports a claim or point that we make about the details of the programme related to IP, product pricing, and other issues, which form part of our analysis. On pp. 17-21, for example, we have added several sentences to explain some of the details and incongruities that are evident in the programme’s underlying legal agreements and refer the reader to the table in the supplementary information.***

9. Page 19: I'd like the authors to expand on their explanation about pricing. As I understand it, the South African hub has to provide any product at an affordable price to people in LMICs but the spokes in LMICs have no obligation to price products at an affordable price. Is my understanding correct? If so why would governments buy from the spokes if they can get the product at a lower price from the hub?

***We agree that this was not clearly explained in our manuscript. We have revised the paragraph to make these details more clear. First, there is a pricing clause in the technology transfer agreements between MPP and the spokes/partners, which stipulate that products that target a Public Health Emergency of International Concern (PHEIC) must be no more than the cost of production plus a 20 percent mark-up. Products that target conditions or diseases that are NOT designated as a PHEIC have no such pricing clause. Second, the “affordability” clause that applies to South African researchers only extends to those who receive funding from the SAMRC as part of the consortium, such as academic researchers at Wits or UCT. The hub, i.e., Afrigen, may not be subject to this commitment unless it receives funding directly from SAMRC, which, to our knowledge, it has not. Instead, all of its funding related to the mRNA programme comes from MPP and there is no pricing clause in that Grant Agreement. These incongruities are highlighted more clearly in the revised paper.***

***The revised paragraph(s) on pp. 20-21 now reads: “***Notwithstanding the leverage the programme’s funding conferred, MPP also stopped short of requiring that resulting mRNA products be priced affordably for populations in need outside of a “Public Health Emergency of International Concern” (PHEIC). If an mRNA product developed by one or more LMIC partners targets a PHEIC, they cannot charge more than the cost of production plus a twenty-percent mark-up.(20) (*see* S3 Table) However, none of the pathogens being targeted by the programme’s partners—from TB to RSV, malaria, and other infectious diseases—are currently designated as a PHEIC. Thus, consistent with industry norms, the mRNA programme does not contractually constrain partners’ pricing decisions. Rather, the assumption is that by targeting LMIC markets, the price of the final product will, of necessity, be affordable; otherwise, LMIC governments will simply not pay for it. “Traditionally,” Charles Gore recalls, “MPP has not interfered in pricing. Our model is based on competition, and clearly we are potentially *giving this* to 15 companies around the world.”(CG, emphasis added)

In contrast, researchers in South Africa who receive funding through the SAMRC must, under the terms of the funding, ensure that any “resulting products”—regardless of whether they target a PHEIC or not—will “be made available and accessible at an affordable price to people most in need within [LMICs]” Revealing differential treatment among participants in the programme, SAMRC-funded researchers and partners with products targeting a PHEIC have agreed to some form of pricing constraint whereas Afrigen has no such obligation unless and until it is in receipt of funding from SAMRC.”

10. Page 31: The authors raise an interesting point that I'd like to see more discussion of. If it is known that Afrigen is interested in developing a vaccine for a disease will that discourage other companies from doing R&D in the same area? Will other companies only compete with Afrigen in financially lucrative disease markets?

***Thank you for the invitation to expand upon this point. It is unclear, at this stage, whether Afrigen will discourage other parts of the programme from pursuing particular lines of R&D, whether for more lucrative disease markets or to address LMIC health needs. Part of the critique we develop in the section about ‘market-based governance’ is that the programme has, until very recently, taken a hands-off approach to decide what R&D to pursue and prioritize. In effect, this means market considerations are likely to remain the main driver of those decisions.***

***In light of this comment, though, we have added a paragraph that highlights the fact that any IP that partners/spokes in the programme gain access to cannot be used, sold, exported or imported to high-income countries (HICs). Instead, under the terms of the technology transfer agreements—with the lone exception of Indonesia’s BioFarma—spokes/partners are only allowed to use, sell, etc. the IP they acquire from MPP in LMICs. Importantly, this doesn’t mean that a partner can’t technically compete with other manufacturers in HIC settings. They just can’t do so using any IP that they’ve acquired from the programme.***

***This new paragraph added at p. 20 reads:*** “A second IP-related incongruity in the programme’s legal architecture concerns the territorial limitations imposed upon IP licenses among different actors involved in the mRNA programme. As the central intermediary, MPP is granted a “worldwide” license to IP that is generated by both members of the South African consortium as well as LMIC partners. In turn, partners (with the exception of Indonesia’s BioFarma) are entitled to receive IP via MPP but only for use, sale, export or import within LMICs. For its part, BioFarma managed to negotiate a “worldwide, non-sublicensable” license to the IP it receives from MPP; it can therefore use, sell, and export such IP globally, but it cannot sub-license it to other entities in LMICs or HICs. That flexibility of licensing IP they generated to companies based in HICs only extends to members of the South African consortium (excluding Biovac as one of the partners). (*see* S3_Table).”

***Drawing upon the details of the Grant Agreement between MPP and Afrigen, we also underscore how the architects of the programme do not expect Afrigen to make its own mRNA products. If they had anticipated that outcome, they likely would have attached similar commitments that were imposed upon spokes/partners through the various technology transfer agreements. The revised paragraph on p. 35 reads in part: “***The architects of the programme appear to have accepted Afrigen’s precarity from the start, anticipating that the Cape Town firm might not survive in the hyper-competitive mRNA marketplace. The absence of pricing commitments parallel to those imposed upon programme partners or a commitment to allocate up to ten percent of “real-time production” capacity in the event of a PHEIC within the four corners of Afrigen’s Grant Agreement with MPP(20)(*see also* S3_Table) could be interpreted as an incentive for Afrigen to commercialize its technology. Read in conjunction with the views of the programme’s architects, the omission of these terms from the Grant Agreement likely suggests that the architects did not contemplate that Afrigen would ever generate mRNA products of its own. Recalling that some of the companies that took part in the influenza hub later shut down production, WHO’s Friede estimates that if a handful LMIC manufacturers manage to make mRNA vaccines, the newer programme will be an overall success.(MF)”…

Minor points

1. There are a number of minor copy-editing problems scattered throughout the manuscript that need to be corrected.

2. Page 19: One of the quotes in the last paragraph is not closed.

***Thank you for catching this. We have now closed the quote in question.***

3. Page 21: Why isn’t Pfizer included in the list of HIC-based mRNA companies?

***We have added Pfizer to this list. The revised sentence now reads:*** *“*However, none of the HIC-based mRNA companies—Pfizer, BioNTech, Moderna, or CureVac—were interested in sharing technology with the global South…*”*

4. Page 29, second line from the bottom: There should be two years named but only one is named.

***We have added “2021” to the statement in question.***

Reviewer #2: This is a useful paper, and will make a helpful contribution to the literature once published, with some relatively minor changes.

This is quite dense material. Some specific recommendations follow, but more broadly, it would be very helpful to have another diagram/figure (perhaps an organigram) outlining the relationships between the parties, and the governance structures.

***Thank you for this suggestion. We have added a new Figure (Figure 2, p. 38) to the paper try and clarify the different relationships and governance structures related to the mRNA programme. We have also attempted to emphasize the differences between how the programme has been described publicly (as a “hub and spoke” model) versus how, based on our findings, the programme actually operates in practice. A detailed note accompanying the figure should also help to clarify a number of key details represented in Figure 2.***

- It is not always clear what conclusions are directly pulled from the interviews, and which are extrapolations derived by the authors. The first full paragraph on p.32 is one example of where this should be made clearer; the ref. to Danaher (flagged in more detail later) is another.

***We appreciate this point and have revised the paragraph in question to make it clear that the concept of a more inclusive and decentralized governance structure derives from the published literature, not our interviews per se (although several participants did raise concerns about the lack of involvement in the programme’s governance, which we also emphasize in the paragraph following the one highlighted by this reviewer).***

***The revised paragraphs now read as follows on p. 36: “***The near inevitability of Afrigen’s exit in the eyes of those who designed the programme speaks to an underlying failure of imagination concerning how the mRNA programme is governed. During the pandemic, calls for more “inclusive and decentralized” governance structures have grown (88) in order to shield initiatives such as the mRNA programme from the risks and constraints posed by dominant market actors. At a minimum, a more inclusive and decentralized structure would involve allowing representatives from participating LMICs capable of steering the programme’s R&D towards local population needs to be directly involved in the programme’s overall governance and day-to-day decision-making, and naming multiple actors to serve as regional mRNA hubs—as originally planned—in order to mitigate the risk that one organization’s failure (or acquisition by an outside actor) might compromise the programme as a whole.

Instead, WHO and MPP internalized programme decision-making within two hand-picked committees, leaned on private actors like Afrigen to play crucial roles, preserved their discretion about what projects and partnerships to pursue, and limited input from LMIC governments and civil society during the programme’s first two-plus years of operation. Members of the South African consortium, including scientists funded by the mRNA programme, also registered concerns about the lack of transparency surrounding the allocation of resources inside the programme. (ALW,ER)”

- The current uniform nomenclature for all anonymized sources is unhelpful. In truth, it may indeed matter whether the XX being quoted on a particular point is, say, a LMIC partner directly involved in this project, or an American academic whose involvement is unknown. I would defer to the authors/the editors on the precise format, but differentiating between all the anonymous voices (XX1, XX2 etc.) would be very helpful. I would consider this an important, and an obligatory, change.

***Thank you for raising this point. We have modified the letter combinations used for our anonymous participants in order to distinguish the type of participant without compromising confidentiality.***

- On the issue of anonymization, some of the descriptors are quite vague, particularly in the final category of global north scientists and other outside experts (e.g., “University researcher”). Appreciating that some discretion may be needed to preserve anonymity, slightly more information would be helpful in evaluating their contributions, especially when balanced against specific individuals whose credentials can be assessed by the reader comparatively easily. For instance, “University researcher” in what field?

***We appreciate the reviewer’s request for a little more context about these participants. However, the mRNA programme has attracted an increasing amount of journalistic inquiry and press coverage. This increases the risk that participants in our study who did so on the condition that we would be careful to protect their confidentiality could be identified when our findings are read alongside the media coverage of the mRNA programme, not to mention by those who are directly involved in the programme. For this reason, we think it is best to provide no further details about the individuals involved in our study beyond the information contained in Table 1. We hope this will be satisfactory.***

- Given the (partial) reliance on Canadian government documents, a bit more discussion of both the disclosed material itself, and its broader implications (e.g., are there conditions govt funders could impose to address some of the concerns the authors raise?) would be welcome.

***For reasons of length, we have chosen not to incorporate more information from the disclosed documents into our paper. The documents in question mainly served to corroborate a number of points that we had been told during interviews, such as funding countries expressing preferences for allocating their funding to specific parts of the programme. However, by making the disclosure package available as a supplementary file, we hope that other researchers may examine the documentation and generate new research questions and insights.***

- A little more discussion of the origins of the “four paradigmatic factors” the authors have “abstracted from a review of literature” would be welcome. A reader seeking to use a similar framework would benefit from a stronger understanding of how it was derived. (If that derivation has already been published elsewhere, such a reference would probably suffice, although the reader could be directed there more clearly).

***Despite many articles talking about the need to change the status quo, to the best of our knowledge, no previous paper has attempted to define factors (or features) that are characteristic of the status quo approach within the biopharmaceutical industry. Yet, a substantial body of scholarly research and evidence about the real-world practices of that industry exists. As such, in order to compare the mRNA programme with those practices, we reviewed relevant literature and evidence and identified four main factors that were described in multiple sources. At the outset of each subsection within our Findings (following a preliminary subsection in which the origins of the mRNA programme are explained), we explain one of the four features, with references to studies that support our claim that the feature in question is indeed part of the status quo. We hope, in turn, that the identification of these four features will be one of the main contributions that our paper makes to the literature.***

***We have modified the opening paragraph of our Findings to make it clear that we derived the features from our own reading of literature and evidence. The revised sentence on p. 9 now reads: “***We first examine the mRNA programme’s origins (2020-2021), and then compare its design to the four paradigmatic features of biopharmaceutical production, which we abstracted from a review of literature and evidence from numerous scholarly disciplines: namely,[…]***”***

- On p.10, reference to “vaccine donation” from the global north could be clarified, as the difference between money to purchase doses and actual physical doses has proven relevant elsewhere in dissecting the COVID-19 response.

***We use the term “vaccine donations” to distinguish COVAX from C-TAP and, in turn, the mRNA programme. In contrast to COVAX, neither C-TAP or the mRNA programme is predicated on donations from the global North (whether actual physical doses or funds to purchase doses). Given that this is simply the background to the mRNA programme, we do not think additional detail is needed here.***

- The same is true for reference to funding “secured” and “committed” on p.15 (perhaps something more tangible like “delivered” as appropriate?).

***On p. 16 we have modified the wording so that the distinction between the commitments they have secured versus the funds that the programme has actually received is more clear. The revised sentences read: “***Charged with the responsibility of fundraising, MPP has secured financial commitments from France, the European Commission, Germany, Norway, Belgium, and Canada alongside the government of South Africa and the African Union.(45) To date, these donors have committed USD 117 million to the programme (with USD 89 million received so far (CN), the majority (73%) of which has been allocated to the consortium, including Afrigen, with the remainder (27%) supporting LMIC partners.***”***

- On p.11, it would be helpful to have further explanation and/or a reference for the mention of growing industry opposition to C-TAP (*appreciating that this mention is within the context of an interview).”

***To clarify, that specific statement about opposition to C-TAP is not based solely upon an interview. It is also supported in journalistic reporting. We have added a reference to a key source and revised the statement to make the link between industry opposition and C-TAP’s approach of redistributing IP more clear. The passage in question on p. 11 now reads: “***Any new IP generated by the hub or its spokes would in turn become part of C-TAP’s pool, thus distributing control to LMIC-based manufacturers as their productive capacity increased. However, as industry opposition to C-TAP grew because of the threat that it posed to IP-holders control over COVID-19 interventions,(27) then-WHO assistant director general (ADG) of access to medicines and health products, Dr. Mariângela Simão, and then-WHO Chief Scientist Dr. Soumya Swaminathan opted to “move the mRNA [programme] away from C-TAP to the ACT-[Accelerator]”—an outcome that MPP’s Kieny also favoured.(EDL)***”***

- On page 16, some clarity is needed regarding Canada’s stipulation that funding be allocated to 4 countries. In numerous public statements, Canada has repeatedly made reference to providing funding to the Hub in Cape Town, with no reference to other spokes/countries. Particularly if, as the current wording suggests, no funding has actually flowed to South Africa, this deserves further explanation. Otherwise, more clarity would be helpful.

***Thank you for noting this. The government records we obtained through our ATI request reveal this selective focus on four countries. However, our statement did not convey that this selection was *in addition to* funding for the hub in Cape Town. We have therefore revised the statement on p. 17 to read as follows: “***The government records obtained through an access to information request reveal that Canada, the second largest donor country, has stipulated that its funding be allocated to the hub in Cape Town and four select countries hosting manufacturers participating in the programme: Senegal, Nigeria, Kenya, and Bangladesh.(47)***”***

- A few minor typos throughout (e.g., a reference to “progamme” on p.28, a word missing in “a shared set commitments” on p.17, or “Médicins” rather than Médecins for MSF on p.8.)

***Thank you for catching these typos. We have corrected these errors in the revised paper.***

- On p.19, I am curious about the authors’ insertion of “[had to]” within a quotation from an interview subject. It is not entirely clear to the reader that this insertion does not change the content or intention of the original speaker’s statement. It would be best if the authors checked their records and confirmed the speaker’s original meaning is indeed accurately conveyed (to be clear, it is possible that it may be, but it reads strangely).

***Thank you for the close attention to our quotations. We double-checked our data and made a minor correction to capture precisely what the participant said even though it’s grammatically incorrect. (That’s why we had modified it slightly in our submission; please note: this is the only instance where we did so). The revised sentence on p. 22 now reads:*** *“*Although they have included such clauses for “many, many years,” one South African government official emphasizes, “it’s really an aspirational clause” because “we haven’t had to yet really test that.”

- The text is particularly dense circa p.15-19, and this reader had to read this portion more than once. As noted above, a figure/diagram might help clarify the relevant relationships in a more readily comprehensible way.

***Thank you for raising this concern. We have revised these pages of the manuscript extensively in an effort to improve clarity. These changes are reproduced in response to various comments above. We also wish to acknowledge that part of the challenge is the highly technical nature of the underlying legal agreements, which, together with our interview findings, we are comparing with the key features of the standard approach to biopharmaceutical production. In an effort to improve clarity without sacrificing important technical details, we have added both a new Figure to the paper and also created a table, which highlights a number of important provisions contained in the various legal agreements that underpin the mRNA programme. Subject to the editors’ views about where this table is best placed, for the time being we have included it as a supplementary file (S3_Table) to the paper due to its large size.***

- On p.22, it is unclear whether the speculation around Afrigen’s behaviour being influenced by Danaher’s actions around TB diagnostics stems from the interview subjects, or is purely that of the authors. This should be clarified.

***Thank you for catching this ambiguity. We have clarified that the acquisition by Danaher was noted by the participant, however, it was not clear that that acquisition informed their decision to switch to another microfluidic device. The revised sentence on p. 24 now reads: “***While Fenner noted that Precision NanoSystems was acquired by Danaher Corporation after Afrigen began using its PNI machine, it was not clear whether Danaher’s record of acquiring products and increasing prices, including for a TB diagnostic test, (60,61) factored into Afrigen’s decision to shift to another microfluidic device.***”***

- The last paragraph of p.25 seems a bit vague and speculative. The subsequent discussion flows better; it may be worth revisiting whatever point the authors are trying to make in this paragraph to better integrate it into the paper.

***Thank you for this feedback. We have revised the section on ‘financialization’ significantly to improve its overall clarity. The revised paragraph referred to by the reviewer (now on pp. 27-28) has been divided into two paragraphs, which read:***

“There is no indication that any of the mRNA programme’s direct participants mirror the financialization that is evident among more established biopharmaceutical companies. Neither Afrigen, nor its primary shareholder Avacare, are publicly traded companies. The same is true of the other private companies partnering with the programme. None of the companies involved are using the tools of finance such as share buybacks as a means to generate returns. While some inputs into the manufacturing process have been secured from outside entities, Afrigen, Biovac, and programme partners in other LMICs are invested in developing their R&D and manufacturing capacities in-house in an effort to increase local production in LMICs. The Belgium company Quantoom, which has locked in partnerships with most LMIC manufacturers in the programme, may be amassing an interest in each partner’s IP. But Quantoom’s machines, which automate the in vitro transcription step of mRNA production, seem to contribute meaningfully to the manufacturing process.

While MPP’s position within the programme is not a symptom of financialization, the role that the foundation plays is analogous to the intermediaries that link together biopharmaceutical R&D and production supply chains. Like the industry’s many intermediaries, MPP’s presence simultaneously adds value to, and imposes a drain upon, the mRNA programme.”

- On p.17, the authors refer to a PHEIC being declared in March 2020; the PHEIC was declared in January 2020, while March was the (more legally nebulous) pandemic declaration. It is not clear which the authors actually intend to use as their reference point.

***We think the statement the reviewer is referring to appears on what is now p. 29 of the manuscript. We have corrected it to refer to January 2020 as opposed to March 2020.***

- The discussion of MPP funding at the bottom of p.29 is a bit unclear. For instance, there is a reference to two figures, “respectively”, but only one year for those figures. It might also be useful to compare MPP funding pre COVID, given the pandemic impacted MPP’s activities even before the mRNA project, rather than solely pre/post mRNA.

***Thank you for noting this. Reviewer 1 also noted the missing reference to a second year. We have corrected the sentence so it’s clear that we are referring to the years 2021 and 2022. Also, we have revised the sentence to make it clear that the 5-6 million Swiss francs was the annual budget prior to the pandemic, which increased markedly thereafter. The sentence on p. 33 now reads:*** “In the five years leading up to the pandemic, MPP’s annual budget was in the range of CHF 5-6 million.(75) Since the pandemic began and the mRNA programme was launched, MPP’s annual income has increased roughly fourfold (CHF 23 million and CHF 19 million in 2021 and 2022, respectively), the number of staff has jumped from 25 to ~~approximately~~ 44, and their budget for external consultants has doubled.(75)***”***

- The discussion of different committees on p.32 is another example where a diagram might help illustrate governance.

***Thank you. The two committees are incorporated into the new Figure 2, which we have added to the paper.***

- The last paragraph before the conclusion (p.33) needed to be read twice. Probably ok, but worth revisiting for clarity just in case.

***We have revised the paragraph in order to improve its clarity. The paragraph (on p. 37) now reads:*** “Collectively, these choices reflect the programme’s alignment with the dominant, market-drive approach to biopharmaceutical production. By design, the programme cannot stop Afrigen or other private companies embedded in the programme (e.g., Quantoom) from capitalizing upon the IP they amass through the programme or, if needed to satisfy their shareholders, sell their stake when the right offer materializes. Importantly, such a transaction would not mean that LMIC partners would be deprived of access to IP generated by Afrigen or other parts of the South African consortium. Similarly, a partner that patents an improvement of an mRNA technology that they receive from the programme cannot claim exclusive ownership over that new IP. Rather, the terms of the various agreements underpinning the programme pre-empt those possibilities. “[I]t’s a self-executing grant-back obligation,” MPP’s head lawyer Park stresses, so “if a spoke partner invents something, patents it, the license automatically comes back to us.”(CP) Notwithstanding this safeguard, however, the prospect of Afrigen exiting the space altogether or other parts of the consortium selling (on a non-exclusive basis) its IP assets to an entity based in an HIC, is in tension with the programme’s stated goals of building local mRNA manufacturing capacity in the global South.”

- The abstract and the set-up reference colonial approaches, but until literally the last sentence of the paper, the direct link to the mRNA hub is not made clear. If this is really intended to be a key takeaway of the paper, it would withstand more discussion.

***We are sympathetic to the reviewer’s point here. We have endeavoured in the paper to emphasize the control over the programme remains rooted in the global North, in terms of the countries that are funding it and the strings they have attached, as well as through MPP’s oversight and design of the programme. The latter has, of course, worked in the service of improving access to medicines in the global South for several years. However, its general approach of voluntary licensing, which it has carried through the mRNA programme, does little to disturb established HIC-based companies’ control over many technologies, including mRNA. In effect, this maintains existing power imbalances between HICs and LMICs. We have not described this as evidence of colonialism, in part, because the programme remains a work in progress. But we hope that our attention to how the programme works, how decisions are made (e.g., the decision to do the non-inferiority study in Marseille, France) and so on conveys a worry that this might be the end result. We have revised the Discussion section of the paper significantly in order to tease out the issue of empowerment as well as some potential ways to reform the mRNA programme. We hope this strengthens our overall analysis.***

- As an aside… while perhaps not relevant to the outcome of the study itself, this reader is a bit curious how the ethics committee felt, in the era of Zoom and of climate change, about conducting 16 interviews on four different continents in person, rather than conducting them all virtually. If there was a clear justification for this bifurcation of interview formats, it may be worth including it.

***We appreciate the reviewer’s concern about climate change. In the lead author’s experience, in-person interviews are often essential at least at the start of a qualitative study in order to build rapport and trust with participants. Especially in a case study where many of the participants are in positions of relative power (e.g., company CEOs; senior government officials), recruiting participants for a virtual interview can be very challenging. It was therefore critical that several of the interviews that were conducted for this project occurred in person, in the span of an extended trip, in Cape Town (where MH travelled on two occasions), Geneva (where MH travelled once), and Bangkok (where MH travelled once). The research ethics boards did not raise any concern with this approach.***

- This reader thinks there is occasionally some strange and/or excessive comma usage, but defers to the journal editors in that regard.

***We revised the manuscript with this comment in mind.***

6. PLOS authors have the option to publish the peer review history of their article ([what does this mean?](https://journals.plos.org/globalpublichealth/s/editorial-and-peer-review-process#loc-peer-review-history)). If published, this will include your full peer review and any attached files.


**Do you want your identity to be public for this peer review?** If you choose “no”, your identity will remain anonymous but your review may still be made public.


For information about this choice, including consent withdrawal, please see our [Privacy Policy](https://www.plos.org/privacy-policy).

Reviewer #1: **Yes:** Joel Lexchin

Reviewer #2: No

[NOTE: If reviewer comments were submitted as an attachment file, they will be attached to this email and accessible via the submission site. Please log into your account, locate the manuscript record, and check for the action link "View Attachments". If this link does not appear, there are no attachment files.]

While revising your submission, please upload your figure files to the Preflight Analysis and Conversion Engine (PACE) digital diagnostic tool, <https://pacev2.apexcovantage.com/>. PACE helps ensure that figures meet PLOS requirements. To use PACE, you must first register as a user. Registration is free. Then, login and navigate to the UPLOAD tab, where you will find detailed instructions on how to use the tool. If you encounter any issues or have any questions when using PACE, please email PLOS at figures@plos.org. Please note that Supporting Information files do not need this step.

*In compliance with data protection regulations, you may request that we remove your personal registration details at any time.* [*(Remove my information/details)*](https://www.editorialmanager.com/pgph/login.asp?a=r)*. Please contact the publication office if you have any questions.*

Attendee panel closed
