# Supplementary material for: ‘Our project, your problem?’ A case study of the WHO’s mRNA technology transfer programme in South Africa
Source: PLOS Glob Public Health. 2024 Sep 23;4(9):e0003173. doi: 10.1371/journal.pgph.0003173 (PMC11419367; doi:10.1371/journal.pgph.0003173)
Supplement: Attachment — Submitted filename: PLOS GPH - Final Rebuttal letter.docx [file pgph.0003173.s006.docx]

Journal Requirements:

Please review your reference list to ensure that it is complete and correct. If you have cited papers that have been retracted, please include the rationale for doing so in the manuscript text, or remove these references and replace them with relevant current references. Any changes to the reference list should be mentioned in the rebuttal letter that accompanies your revised manuscript. If you need to cite a retracted article, indicate the article’s retracted status in the References list and also include a citation and full reference for the retraction notice.

[Note: HTML markup is below. Please do not edit.]

Reviewers' comments:

Reviewer's Responses to Questions

**Comments to the Author**

1. If the authors have adequately addressed your comments raised in a previous round of review and you feel that this manuscript is now acceptable for publication, you may indicate that here to bypass the “Comments to the Author” section, enter your conflict of interest statement in the “Confidential to Editor” section, and submit your "Accept" recommendation.

Reviewer #1: (No Response)

Reviewer #2: All comments have been addressed

2. Does this manuscript meet PLOS Global Public Health’s [publication criteria](https://journals.plos.org/globalpublichealth/s/criteria-for-publication)? Is the manuscript technically sound, and do the data support the conclusions? The manuscript must describe methodologically and ethically rigorous research with conclusions that are appropriately drawn based on the data presented.

Reviewer #1: Yes

Reviewer #2: Yes

3. Has the statistical analysis been performed appropriately and rigorously?

Reviewer #1: N/A

Reviewer #2: N/A

4. Have the authors made all data underlying the findings in their manuscript fully available (please refer to the Data Availability Statement at the start of the manuscript PDF file)?

The [PLOS Data policy](https://journals.plos.org/globalpublichealth/s/data-availability) requires authors to make all data underlying the findings described in their manuscript fully available without restriction, with rare exception. The data should be provided as part of the manuscript or its supporting information, or deposited to a public repository. For example, in addition to summary statistics, the data points behind means, medians and variance measures should be available. If there are restrictions on publicly sharing data—e.g. participant privacy or use of data from a third party—those must be specified.

Reviewer #1: Yes

Reviewer #2: Yes

5. Is the manuscript presented in an intelligible fashion and written in standard English?

PLOS Global Public Health does not copyedit accepted manuscripts, so the language in submitted articles must be clear, correct, and unambiguous. Any typographical or grammatical errors should be corrected at revision, so please note any specific errors here.

Reviewer #1: Yes

Reviewer #2: Yes

6. Review Comments to the Author

Please use the space provided to explain your answers to the questions above. You may also include additional comments for the author, including concerns about dual publication, research ethics, or publication ethics. (Please upload your review as an attachment if it exceeds 20,000 characters)

Reviewer #1: The authors’ revisions have dealt with my initial concerns. I have two further suggestions for areas to comment on (although these are not essential to include) and there are a number of minor issues.

Issues to consider:
Page 27: Do the authors want to talk about how financialization results in turning patents and IP into assets to be bought and sold thereby increasing the price of any eventual product?

***Thank you for the suggestion. Given that the paper is lengthy and the fact that we significantly added to the discussion on financialization in the previous round of review and revision, we have decided not to add this element to our analysis.***

Page 33: Do the authors want to get into a discussion of negative aspects of involving the BMGF in funding vaccine projects - see The Bill Gates Problem by Tim Schwab?

***As the BMGF was not a core focus within our study, we think it would be inappropriate to expand the discussion at this stage. We have, however, cited to literature, including Schwab’s book, concerning BMGF.***

Minor issues:

Page 10: The authors should make it clear that contributions to C-TAP were to be made on a voluntary basis.

***We agree with this suggestion and have revised the section in question to read as follows: “***In contrast to ACT-A’s charity-based approach, C-TAP sought to distribute control of the intellectual property (IP), data, and knowledge related to COVID-19 interventions. Pooling a variety of technologies through voluntary licenses, vaccine and other product manufacturers could in-license technologies to address population needs in LMICs (30) rather than depending on vaccine donations from HICs—a move applauded by civil society but fiercely contested by industry, its allies, and the Gates Foundation.(31,32)***”***

Page 15: It should be "definition" not "defining".

***Thank you for catching this typo. However, the issue was that the word ‘feature’ was missing after ‘defining’. We have now corrected this.***

Page 16, second paragraph: Are the authors talking about all phases of biopharmaceutical R&D or just basic research being publicly funded?

***Good point. We have added the qualifying phrase ‘early stage’ to this statement for accuracy and clarity.***

Page 25, line 5: It should be "member" not "member's".

***Thank you. We have corrected this typo.***

Page 34: Is it Afrigen or Afrigen's principal shareholder Avacare that has been having trouble securing additional funding?

***As we state in the manuscript, it is Afrigen that has applied for, but not secured, additional funding. Avacare’s financial status was not a primary focus in the interviews we conducted for this paper. No changes were therefore made in response to this comment.***

Page 35, 8th line from bottom: Insert "of" between "handful" and "LMIC".

***We have corrected this typo.***

Page 36: The sentence starting "At a minimum..." is very long and difficult to follow. The authors should break it up.

***Point taken. We have broken down the sentence into three sentences, which read as follows: “***At a minimum, a more inclusive and decentralized structure would entail two key changes to the programme. First, representatives from participating LMICs capable of steering the programme’s R&D towards local population would need to be directly involved in the programme’s overall governance and day-to-day decision-making. Second, multiple actors would need to serve as regional mRNA hubs—as originally planned—in order to mitigate the risk that one organization’s failure (or acquisition by an outside actor) might compromise the programme as a whole.***”***

Reviewer #2: Good paper, all major concerns addressed. Two exceedingly minor comments. First, the section "The Sustainability Question: Market-Based Governance" in particular should be re-read with an eye to grammatical typos, e.g., "Friede estimates that if a handful [of] LMIC manufacturers"; "to validate the use [of] funds from one source"; "in the service of the public health" etc. Second, Figure 2, especially the second portion, is very difficult to read at 100% size; if it's anticipated anyone would ever read it without being able to zoom a PDF, it might be worth making it larger.

***Thank you for these comments. We have re-read that section with a view to correcting typos. We have also uploaded Figure 2 to the PACE tool online and believe it meets the journal’s technical requirements. As the article moves toward publication, we trust that the journal will help format the figure as a PDF that can be enlarged for ease of viewing by readers.***

7. PLOS authors have the option to publish the peer review history of their article ([what does this mean?](https://journals.plos.org/globalpublichealth/s/editorial-and-peer-review-process#loc-peer-review-history)). If published, this will include your full peer review and any attached files.


**Do you want your identity to be public for this peer review?** If you choose “no”, your identity will remain anonymous but your review may still be made public.


For information about this choice, including consent withdrawal, please see our [Privacy Policy](https://www.plos.org/privacy-policy).

Reviewer #1: **Yes:** Joel Lexchin

Reviewer #2: No

While revising your submission, please upload your figure files to the Preflight Analysis and Conversion Engine (PACE) digital diagnostic tool, https://pacev2.apexcovantage.com/. PACE helps ensure that figures meet PLOS requirements. To use PACE, you must first register as a user. Registration is free. Then, login and navigate to the UPLOAD tab, where you will find detailed instructions on how to use the tool. If you encounter any issues or have any questions when using PACE, please email PLOS at figures@plos.org. Please note that Supporting Information files do not need this step.
